# Sedative-Hypnotic Effect and Mechanism of Carbon Nanofiber Loaded with Essential Oils of *Ligusticum chuanxiong* (*Ligusticum chuanxiong* Hort.) and Finger Citron (*Citrus medica* L. var. *sarcodactylis*) on Mice Models of Insomnia

**DOI:** 10.3390/biom14091102

**Published:** 2024-09-02

**Authors:** Yue Hu, Xiaofang He, Yuanyuan Wu, Wenjie Zhang, Huiyi Feng, Haolin Liu, Qianqian Wu, Leying Gao, Yu Long, Xiaoqiu Li, Jie Deng, Yin Ma, Nan Li

**Affiliations:** State Key Laboratory of Southwestern Chinese Medicine Resources, Chengdu University of Traditional Chinese Medicine, Chengdu 610075, China; huyue@stu.cdutcm.edu.cn (Y.H.); hexiaofang@stu.cdutcm.edu.cn (X.H.); wuyuanyuan@stu.cdutcm.edu.cn (Y.W.); huyue1@stu.cdutcm.edu.cn (W.Z.); fenghuiyi@stu.cdutcm.edu.cn (H.F.); liuhaolin@stu.cdutcm.edu.cn (H.L.); wuqianqian@stu.cdutcm.edu.cn (Q.W.); leyinggao@stu.cdutcm.edu.cn (L.G.); longyu@stu.cdutcm.edu.cn (Y.L.); lixiaoqiu@stu.cdutcm.edu.cn (X.L.); dengjie@stu.cdutcm.edu.cn (J.D.); mayin@stu.cdutcm.edu.cn (Y.M.)

**Keywords:** sedative -hypnotic effects, *Ligusticum chuanxiong* Hort essential oil, *Citrus medica* L. var. sarcodactylis essential oil, carbon nanofiber, inhalation

## Abstract

(1) Background: Insomnia is a neurological illness that poses a significant threat to both physical and mental health. It results in the activation of neuroglial cells, heightened neuroinflammation, oxidative stress, and disruptions in the Hypothalamic–Pituitary–Adrenal (HPA) axis. *Ligusticum Chuanxiong* (CX) and Finger citron (FC) are frequently utilized botanicals for addressing sleeplessness. Both herbs possess notable anti-inflammatory properties in their volatile oils. However, their effectiveness is hindered by the nasal mucosal irritation and instability they exhibit. (2) Methods: This study involved the preparation of a nanofiber composite system using carbon nanofiber (CNF) suspensions containing essential oils of *Ligusticum chuanxiong*–Finger citron (CXEO-FCEO-CNF). The effects and mechanisms of these essential oils in improving insomnia were investigated using an insomnia mouse model after encapsulation. (3) Results: The CXEO-FCEO-CNF had an average particle size of 103.19 ± 1.64 nm. The encapsulation rates of essential oils of *Ligusticum chuanxiong* (CXEO) and essential oils of Finger citron (FCEO) were 44.50% and 46.15%, respectively. This resulted in a considerable improvement in the stability of the essential oils over a period of 30 days. The essential oils effectively decreased the irritation of the nasal mucosa following encapsulation. Furthermore, CXEO-FCEO-CNF enhanced voluntary activity and sleep in mice with insomnia, notably boosted the activity of superoxide dismutase (SOD), reduced the concentration of lipoxidized malondialdehyde (MDA), decreased the levels of hormones associated with the HPA axis, and regulated the levels of neurotransmitters, resulting in a beneficial therapeutic outcome. CXEO-FCEO-CNF contains a total of 23 active ingredients, such as alpha-Asarone, (E)-methyl isoeugenol, and Senkyunolide. These ingredients primarily work by modulating the Janus kinase-signal transducer and activator of transcription (JAK-STAT) signaling system to decrease oxidative stress and inflammatory reactions. (4) Conclusions: This study presented initial evidence that the combination of CXEO and FCEO in nanofiber formulations effectively reduces the nasal mucosal irritation and instability of essential oils. Furthermore, it demonstrated the potential anti-neuroinflammatory and therapeutic effects of these formulations in treating insomnia. Overall, this study provides a theoretical foundation for developing new essential oil formulations derived from herbs.

## 1. Introduction

Insomnia is a neurological problem that is characterized by sensations of worry and depression. This condition can lead to cardiovascular damage, neurodegenerative diseases, and diabetes [1,2,3,4,5]. According to World Health Organization estimates, the worldwide occurrence of insomnia is as high as 27% [6,7]. As a pervasive and enduring illness of worldwide significance, it profoundly impacts the physical and emotional well-being of billions of individuals. Nevertheless, medicines like diazepam (DZP) and triazolam, frequently employed in clinical settings, are plagued by the therapeutic disadvantages of dependence and addiction. Herbal medicines have gained recognition as potential treatments for cancer, cardiovascular diseases, and neurological disorders. This is because they contain a variety of chemicals that may have combined therapeutic effects on diseases through multiple pathways. They also offer possibilities and resources for the development of neurotherapeutic agents. Valerian plant extracts have been found to affect the gamma-aminobutyric acid (GABA)-ergic system, which leads to a decrease in nervous system excitability and produces a sedative-hypnotic effect [8]. Furthermore, certain research has indicated that the substance sour jujube nut soup has sedative-hypnotic effects by altering 5-hydroxytryptamine receptor 1A and HPA axis homeostasis [9]. Hence, it is crucial to investigate the bioactive compounds derived from medicinal plants for the management of neurological disorders.

Ligusticum chuanxiong Hort., belonging to the Umbelliferae family, and Finger citron (*Citrus medica* L. var. sarcodactylis Swingle), belonging to the Rutaceae family, are the primary herbs used in the sedative-hypnotic formula. The combination of these two herbs may produce synergistic effects. The primary active components of Ligusticum chuanxiong and Finger citron are their volatile oils, which are abundant in monoterpenes and sesquiterpenes. The essential oils derived from Ligusticum chuanxiong and Finger citron possess notable sedative and hypnotic properties. Studies have shown that CXEO effectively suppresses the release of substances that cause inflammation. Additionally, it exhibits notable anti-inflammatory and antidepressant properties [10,11]. FCEO has the ability to decrease the release of interleukin-1 beta (IL-1β), interleukin-6 (IL-6), and tumor necrosis factor-alpha (TNF-α) [12,13]. Specifically, linalool and linalyl acetate, two components of FCEO, can enhance the activity of SOD, decrease the infiltration of inflammatory cells, and mitigate the inflammatory damage to brain tissue [14]. Inhaling essential oils through the nose offers a distinct benefit when it comes to treating neurological problems. Essential oils have the ability to bypass the blood–brain barrier and directly affect brain tissues by using the nasoencephalic channel. This pathway allows the oils to reach specific areas of the brain, including the cerebral cortex, hypothalamus, and limbic system [15].

Nevertheless, the use of essential oils is restricted due to their limited stability and potential to cause irritation to the skin and mucous membranes. Utilizing nanofiber technology, medications can be delivered to the brain through the nasal passage, resulting in enhanced drug use. Nanofibers exhibit exceptional biocompatibility [16]. Their significant surface area and ability to adhere to biological surfaces can extend the duration of the medication’s presence in the nasal cavity, interact with the nasal cilia, and promote the gradual release of the drug [17,18]. According to reports, a novel kind of nasal drops containing short fibers has been shown to enhance the nasal mucosa’s capacity to administer medications for the treatment of central nervous system disorders [19]. Nanofiber compositions offer benefits in enhancing the stability and reducing the skin mucosal irritation of essential oils delivered intranasally. Nanofiber emulsion systems including essential oils may offer a novel approach for treating insomnia by nasal administration.

The aim of this study is to assess the effectiveness of nanofibers in enhancing the stability and delivery efficiency of essential oils from Ligusticum chuanxiong and Finger citron in a mouse insomnia model. Additionally, the chemical composition of CXEO-FCEO will be analyzed using gas chromatography–mass spectrometry (GC-MS) to predict the targets and pathways of action of the main components of the essential oils through network pharmacology. The findings will be validated using Enzyme-linked immunosorbent assay (ELISA) and Western blot (WB) methods. Ultimately, this research aims to provide a theoretical basis for the use of CXEO-FCEO-CNF inhalation in improving insomnia.

## 2. Materials and Methods

### 2.1. Chemicals and Reagents

CX was acquired from the Chinese herbal medicine market located in Hehuachi, Chengdu, China. FC was acquired from Taiping Town Plantation Farm, located in Leshan City, China. The purchase of diazepam was made from Jining Ankang Pharmaceutical Co. (Jining, China). DL-4-Chlorophenylalanine (PCPA) was acquired from Shanghai Yuanye Bio-technology Co. (Shanghai, China). The acquisition of pentobarbital sodium was made from Sichuan Huaxia Chemical Reagent Co., Ltd. (Chengdu, China). The SOD activity assay kit and Lipid Peroxidation MDA assay kit were acquired from Shanghai Biyuntian Biotechnology Co., Ltd. (Shanghai, China). The 5-Hydroxytryptamine (5-HT) ELISA kit was acquired from Elabscience Biotechnology Co., Ltd. (Wuhan, China). The Norepinephrine (NE) ELISA kit, Dopamine (DA) ELISA kit, and GABA ELISA kit were purchased from Quanzhou Ruixin Biotechnology Co., Ltd. (Quanzhou, China). The Corticotropin-Releasing Hormone (CRH) ELISA kit, Adrenocorticotropic Hormone (ACTH) ELISA kit, and Corticosterone (CORT) ELISA kit were purchased from Quanzhou Ruixin Biotechnology Co., Ltd. The Janus Kinase 2 (JAK2) antibody was acquired from Cell Signaling Technology, while the Signal transducer and activator of transcription 3 (STAT3) antibody was obtained from Chengdu Zhengneng Biotechnology Co., Ltd. (Chengdu, China). The Phospho-JAK2 (P-JAK2) antibody, Phospho-STAT3 (P-STAT3) antibody, and Suppressor of Cytokine Signaling 3 (SOCS3) antibody were purchased from Shenzhen Youpin Biotechnology Co., Ltd. (Shenzhen, China).

### 2.2. Animals

Male ICR mice weighing between 25 and 35 g were acquired from Sibeifu (Beijing, China) Bio-technology Co., Ltd. The animals (SCXK (Jing) 2024–0001) were kept in a controlled environment with a constant room temperature of 25 ± 2 °C and a humidity of 50 ± 5%. They were exposed to 12 h of light followed by 12 h of darkness in a daily cycle. They were provided with food and water for a period of 7 days in a manner that was suited to their needs. The animal experiments we conducted were approved by the animal and ethics review committee of Chengdu University of Traditional Chinese Medicine (China, approval No. 2020–0312) and adhered to the “Laboratory Animal Care and Use Guide” (NIH Publication # 85–23, revised 1996) published by the National Institutes of Health.

### 2.3. Investigation of the CXEO-FCEO-CNF Preparation Process

#### 2.3.1. Preparation of CXEO-FCEO-CNF

**Figure 1:**
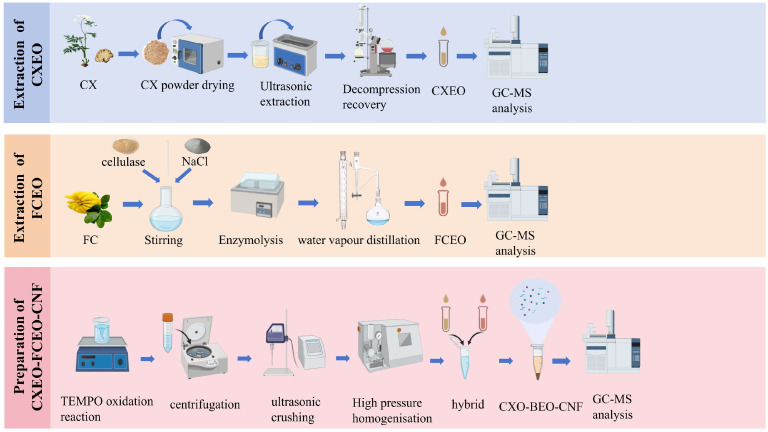
Preparation of CXEO-FCEO-CNF.

The nanofiber suspension was created through the process of chemical oxidation and high-pressure homogenization [20]. A solution of 0.5 M sodium hydroxide was produced beforehand for adjusting the pH. A quantity of 1 g of microcrystalline cellulose was measured and mixed with 100 mL of ultrapure water at room temperature for 10 min. Then, 0.04 g of 2,2,6,6-tetramethylpiperidine-1-oxyl radical (TEMPO) and 0.64 g of NaBr were added to the cellulose suspension. Following the complete dissolution of TEMPO, sodium hypochlorite was incrementally added to commence the oxidation process. The pH of the control system was modified to a range of 10 to 11 by adding a 0.5 mol/L NaOH solution during the procedure. The oxidation of TEMPO was synergized using ultrasound as the method of processing. The system was thereafter positioned on a magnetic stirrer, and the reaction was permitted to undergo continuous stirring for a duration of 26 h at a temperature of 25 degrees Celsius and a speed of 300 revolutions per minute. Ultimately, the process was halted by adding 3 mL of anhydrous ethanol. CNF gel was obtained using centrifugation at 8000 revolutions per minute for 10 min. It was then mixed with 10 milliliters (mL) of water and subsequently subjected to dialysis using a dialysis bag with a molecular cut-off of 6000–8000. The dialysis process lasted for 3 days, during which no precipitation was observed. The lack of chloride ions was confirmed by testing with silver nitrate. A 10 mL suspension of CNF was subjected to sonication for 10 min using a cell crusher operating at a temperature of 10 °C and a power of 450 W at 70% intensity. The clear nanofibrillar solution, obtained after sonication, underwent high-pressure homogenization at a temperature of 4 °C for 30 min. The homogenization process involved applying a pressure of 1800 Bar for 30 cycles. Throughout the homogenization process, the temperature was maintained at 4 °C by circulating cooling water. The result was a stable, transparent, and uniform nanofibrillar suspension. This suspension was then stored at 4 °C for future use. The loading technique of essential oils is a reference to the preceding procedure [21,22]. The nanofiber suspension was continuously stirred for 24 h to achieve a homogeneous distribution. CXEO and FCEO were then added to the suspension in a 1:1 ratio, aided by a certain amount of Tween-80 to enhance solubility. The mixture was further sonicated using an ultrasonic machine in an ice bath for 5 min to obtain the composite essential oil nanofiber system. The preparation of CXEO-FCEO-CNF is shown in Figure 1.

#### 2.3.2. Determination of Encapsulation Efficiency (EE)

The determination of the encapsulation rate of essential oils by the nanofiber system was conducted using the organic solvent extraction method. Take 0.5 mL of the prepared CXEO-FCEO-CNF solution in a test tube. Add 2 mL of petroleum ether for three consecutive extractions. Each time, properly mix the extraction and let it stand for 45 min. After completing the extraction process, remove the upper layer of the petroleum ether solution and retain the lower layer. Introduce methanol through ultrasonication to disrupt the internal structure of the system. Determine the concentration of the encapsulated essential oils using a UV spectrophotometer (W_B_). The wavelength of CXEO detection was 338 nm, while that of FCEO was identified at 234 nm. The absorbance value of the unextracted CXEO-FCEO-CNF methanol breakage solution was determined using the same procedure. The total essential oil content (W_T_) in the system was then computed, and the EE was calculated using the following equation.

$$EE\%=\frac{W_{B}}{W_{T}}\times 100\%$$

#### 2.3.3. Optimization of CXEO-FCEO-CNF Preparation by Response Surface Box–Behnken Design (RS-BBD)

An investigation was conducted to study the impact of different factors, namely, NaClO dosage, TEMPO dosage, NaBr dosage, ultrasonic power, and essential-oil-to-nanofiber ratio, on the particle size of CXEO-FCEO-CNF. Correlation analyses were carried out using IBM SPSS Statistics 25. The one-way test findings revealed the ultrasonic power, NaClO dosage, and essential-oil-to-nanofiber ratio as the three primary parameters that influence particle size. Each of the three criteria was assigned to three distinct levels (Table 1). The response variables utilized to evaluate CXEO-FCEO-CNF were particle size, EE1 (encapsulation rate of CXEO), and EE2 (encapsulation rate of FCEO).

#### 2.3.4. Characterization of CXEO-FCEO-CNF

The morphology of CXEO-FCEO-CNF was assessed using transmission electron microscopy (TEM) with a JEOL JEM-1400FLASH TEM instrument manufactured by Shimadzu in Japan. The samples were treated with a 1% (*w*/*v*) solution of phosphotungstic acid (pH = 7.0) for 1–2 min at room temperature. After drying, they were imaged using TEM. Furthermore, the nanoparticle size meter (Anton Paar, Litesizer 500, Graz, Steiermark, Austria) was utilized to assess the average particle size, particle size distribution, and polydispersity index (PDI) of CXEO-FCEO-CNF. The infrared spectral analysis of CXEO, FCEO, CXEO-FCEO-CNF, and CNF was conducted using a Nicolet 6700 Fourier transform infrared spectroscopy (FT-IR) spectrometer instrument. Approximately 2 mg of each sample (CXEO, FCEO, CXEO-FCEO-CNF, and CNF) and 100 mg of potassium bromide were homogeneously ground together. The resulting mixture was then pressurized to 20 MPa for tabletting. The samples were subsequently analyzed for infrared testing.

#### 2.3.5. In Vitro Release and Stability Study of CXEO-FCEO-CNF

The extent of nanofiber release in vitro was measured using the dialysis technique. The release of CXEO-FCEO-CNF was conducted in vitro utilizing the dialysis bag method and an intelligent dissolving device (Tianjin Tianda Tianfa Science and Technology Co., Ltd., ZRS—8 G, Tianjin, China). The release medium used was Phosphate-Buffered Saline (PBS) with a pH of 6.5. The produced CXEO-FCEO-CNF was examined in a simulated nasal mucus environment at a temperature of 37 °C and a pH of 6.5 to study its release behavior. The dialysis bag, which contained 2 mL of CXEO-FCEO-CNF, was immersed in 400 mL of PBS and released by gently stirring at a speed of 60 revolutions per minute at a temperature of 37 ± 0.5 degrees Celsius. At intervals of 0.5, 2, 4, 6, 8, 12, 24, 36, 48, 60, and 72 h, 4 mL of the dissolution was extracted and substituted with an equivalent volume of fresh PBS. Ultimately, the extent of drug release was assessed using UV spectrophotometry. Meanwhile, the CXEO solution and FCEO solution were produced and dialyzed according to the previously mentioned instructions. Furthermore, CXEO-FCEO-CNF was synthesized using the refined optimum technique, and its stability was assessed by measuring the particle size and PDI after storing it at 4 °C and 25 °C for 0, 5, 10, 15, 20, and 30 days, respectively.

### 2.4. In Vivo Pharmacodynamic Study

#### 2.4.1. Establishment of Mouse Insomnia Model and Group Administration of Drugs

**Figure 2:**
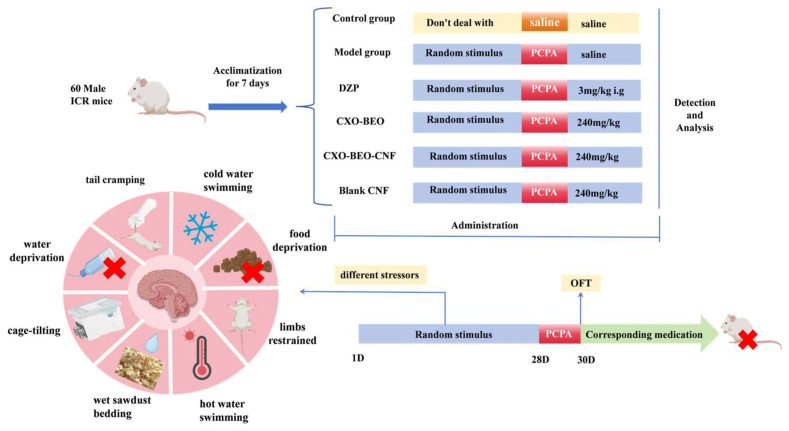
Animal experimental protocol of this study. PCPA, DL-4-Chlorophenylalanine. DZP, diazepam. CXO-BEO, Essential Oils of *Ligusticum Chuanxiong* (*Ligusticum chuanxiong* Hort.) and Finger Citron (*Citrus medica* L. var. sarcodactylis). CXO-BEO-CNF, carbon nanofiber suspensions containing essential oils of *Ligusticum chuanxiong*–Finger citron. CNF, carbon nanofiber suspensions. OFT, Open-Field Test.

Figure 2 demonstrates the random division of all male ICR mice into six groups, each consisting of 10 mice (*n* = 10). The groups included in the study were the control group, model group, DZP group (administered at a dose of 3 mg/kg, Diazepam), CXEO-FCEO group (pure essential oil group), CXEO-FCEO-CNF group (essential oil nanofiber group), and CNF group (blank nanofiber group). This work utilized the chronic unpredictable mild stress (CUMS) approach paired with the intraperitoneal injection of PCPA to create an insomnia mouse model that accurately simulates the effects of excessive mental stress in humans [9,23]. In order to ensure the unpredictability of the stressors, the mice were subjected to chronic stressors in a random manner, with the exception of the control group. The stressors consisted of tail spasms lasting for 1 min, swimming in cold water at a temperature of 4 °C for 5 min, being deprived of food for 24 h, being deprived of water for 24 h, experiencing a reversal of the circadian rhythm for 24 h, being exposed to wet woodchip pads for 24 h, being subjected to cage tilting at a temperature of 45 °C for 24 h, and being restrained in the limbs for 24 h. The mice were subjected to one stressor per day for a total of 28 days, ensuring that the same stressor was not repeated on consecutive days. The mice were administered an intraperitoneal injection of a suspension of PCPA (30 mg/mL) at a dosage of 300 mg/kg at 13:00 on days 29–30. The model exhibited physiological traits of irritation and heightened voluntary activity following effective modeling [24]. During the modeling process, both the control group and the model group of mice were given the same amount of saline (1 mL/100 g) through oral administration. The remaining mice were given the corresponding medication through inhalation in an aromatherapy room once a day for 28 consecutive days. Subsequently, blood was extracted via the orbital cavity. Following the execution of the mice, their brain tissues were gathered and preserved in a refrigerator at a temperature of −80 °C.

#### 2.4.2. Assessment of Body Weight, Food Consumption, Body Temperature, and Grip Strength in Mice

Observations were made on the cognitive and behavioral patterns of mice in each group following the modeling and treatment process. Additionally, the body weights of mice were collected both before the modeling process and after the final administration of the drug. The diurnal and nocturnal food consumption of mice in each group was documented throughout the final week of medication treatment. The food intake was from 9:30 a.m. to 21:30 p.m., and the night food intake time period was from 21:30 to 9:30 (next day). Food consumption was quantified by weighing and documenting the quantity of unconsumed food in each group. The body temperature of mice in each group was assessed. Furthermore, a grip strength assessment was conducted on every group of mice.

#### 2.4.3. Behavioral Tests

##### Open-Field Test, OFT

An OFT was conducted on the second day prior to the completion of the dosage [25]. After 30 min of administration, mice from each group were exposed to an open field test. The mice were introduced to the laboratory environment for a period of 5 min to adapt and become accustomed to their surroundings. The experimental configuration consisted of a black square cage measuring 50 cm in length, 50 cm in width, and 40 cm in height. At the start of the measurement, every mouse was positioned in the middle of the apparatus, and at the completion of the measurement, the device was cleansed with alcohol. The computer-aided picture processing system recorded their free exploration for 5 min after 1 min of instruction. The system’s single-object tracking mode was used to measure the overall distance traveled, resting time, and average velocity of each mouse.

##### Forced Swimming Test, FST

Each mouse was placed in a cylindrical glass container of 25 cm in height and 10 cm in diameter. The container was filled with 20 cm of water. The mice were carefully placed in the water containers at a temperature of 24 ± 1 °C, ensuring that their tails did not touch the bottom. Following a 2 min period of acclimatization, the mice’s resting time was measured for the subsequent 4 min. The resting time was determined as the duration during which the mice remained buoyant in the water without making any significant efforts, just performing the essential movements to maintain their heads above the water surface.

##### Tail Suspension Test, TST

During the experiment, a strip of tape was placed 2.5 cm away from the tail tip of the mice. The mice were then suspended around 40 cm above the ground for a duration of 6 min. Following a 2 min period of acclimation, the resting time of the mice was measured for the subsequent 4 min. The resting time was determined as the period during which the mice ceased their efforts and exhibited minimal body movement.

#### 2.4.4. Pentobarbital Sodium-Induced Sleep Test

The pentobarbital sodium-induced sleep test in mice is a widely employed behavioral technique for evaluating the sedative-hypnotic properties of a medication [26]. The sleep test caused by sodium pentobarbital was conducted one day prior to the completion of drug administration. After a period of thirty minutes, sodium pentobarbital was administered via intraperitoneal injection. The dosage used was 55 mg/kg pentobarbital, which had been pre-tested and determined to be a suprathreshold dose. The medication was provided just prior to usage. The mice experienced a loss of righting reflex at the beginning of sleep that lasted for over 1 min. The duration of this loss and the time it took for the righting reflex to recover, known as sleep latency and sleep duration, were measured for each mouse. Sleep latency (unit: s) was defined as the duration between the administration of pentobarbital sodium and the loss of the righting reflex. The sleep duration (unit: s) was utilized as the temporal gap between the cessation and restoration of the righting reflex.

#### 2.4.5. Measurement of SOD Activity and MDA Content

Mouse brain tissue was obtained and mixed with ice saline in a 1:9 ratio. The mixture was then subjected to centrifugation at 8000 r·min^−1^ and 4 °C for 10 min. The resulting supernatant was collected, and the activity of SOD and the content of MDA were measured using the methods provided in the kit manufacturer’s instruction manual.

#### 2.4.6. Analysis of Serum Biochemical

Following the experiment’s conclusion, 28 days following the treatment, all ICR mice underwent a 12 h fasting period. To obtain serum, blood samples were taken and subjected to centrifugation in a high-speed centrifuge at 4 °C and 3500 rpm/min for 10 min. ELISA kits were used to measure the serum levels of CRH, ACTH, and CORT, following the instructions provided by the manufacturer.

#### 2.4.7. Measurement of Neurotransmitters

Upon the completion of the experiment, the hypothalamus tissue was promptly and cautiously extracted and preserved at a temperature of −80 °C. A mixture of hypothalamic tissue was generated by combining PBS with the tissue at a ratio of 1:9. The tissue homogenate was subjected to centrifugation at a speed of 3500 revolutions per minute for a duration of 10 min in order to extract the supernatant. The concentrations of 5-HT, GABA, DA, and NE in the hypothalamus were measured using an ELISA kit, following the instructions provided by the manufacturer.

#### 2.4.8. Evaluation of Safety in Nasal Drug Administration

The mice used in the experiment were separated into four groups (*n* = 3) in a random manner: a group receiving saline, a group receiving a 1% sodium deoxycholate solution, a group receiving a CXEO-FCEO essential oil solution, and a group receiving a CXEO-FCEO-CNF solution. Each group of mice received the appropriate medications on a daily basis for a continuous period of 28 days. The mice were euthanized 24 h after the final drug administration. The skin was removed to expose the maxilla and detach it from the skull. A cut was made along the midline of the nose to expose the nasal diaphragm and both nasal cavities. The anterior-middle nasal diaphragm was then removed and promptly fixed in 4% neutral paraformaldehyde for 24 h to facilitate subsequent pathological analysis. The thoracic and abdominal cavities of mice were dissected, and the heart, liver, spleen, lung, and kidney tissues were extracted. The fascia and other debris were meticulously eliminated, and the tissue surfaces were rinsed with chilled saline solution to remove any remaining blood. Subsequently, the surfaces were dried using filter paper, and the tissues were promptly preserved in 4% neutral paraformaldehyde for 24 h to facilitate subsequent pathological analysis.

### 2.5. Analysis of the Chemical Composition of Essential Oil

CXEO was extracted by an extraction method. CX crude powder was sieved through a 20-mesh sieve and then dried at 55 °C for 3 h. Then, it was mixed with 1:10 (*w*/*v*) ether and placed in an ultrasonic bath for 40 min. The ether extract was collected by filtration. The ether in the extract was removed by rotary evaporation under reduced pressure until there was no odor of ether, and the residual CXO was collected. The GC-MS settings used for analyzing CXEO were as follows: The solution was diluted to a volume of 1 mL using ethyl acetate and then passed over a microporous membrane with a pore size of 0.45 μm. The Agilent HP-5 column is composed of 5%-phenyl)-methylpolysiloxane and has dimensions of 30 m × 250 μm × 0.25 μm. The carrier gas used is helium (He), with a flow rate of 1 mL/min. The planned temperature increase was configured as follows: The column temperature was initially set at 50 °C and kept for 3 min. It was then increased to 150 °C at a rate of 5 °C/min, followed by a rise to 250 °C at a rate of 10 °C/min. The temperature was then increased to 260 °C at a rate of 25 °C/min and held for 5 min. The shunt ratio is 100:1 and the injection volume is 1 μL. The mass spectrometry was performed under the following conditions: electron ionization (EI) with an electron energy of 70 eV, ion source temperature of 220 °C, and the complete scanning mode.

The FCEO was obtained through the process of steam distillation. The recently harvested outer layer of the fruit was sliced, dehydrated, and crushed using a sieve with 60 evenly spaced holes per square inch to produce the powdered outer layer. By measuring 45 g of the powder and placing it in a 2000 mL round-bottomed flask, adding 2% NaCl, adding 1300 mL of ultrapure water in a 1:4 ratio, and then adding 0.7% cellulase enzyme, the enzyme temperature was set to 52 °C. The enzyme pH was adjusted to 5.2, and the mixture was stirred using a glass rod. The flask was then sealed and placed in a water bath to carry out the enzyme digestion at a predetermined temperature and time. Once the enzymatic treatment concluded, a few grains of zeolite were introduced into the round-bottomed flask. Subsequently, the essential oil extractor and reflux condenser tube were attached, and the distillation apparatus was assembled. To obtain the upper layer of essential oil, follow these steps: gradually heat an electric heating jacket until it reaches the boiling point, maintain a slight boil for approximately 3 h, and cease heating and allow it to stand for 1 h. Collect the condensate and let it stand for a specific duration. Under the conditions of 6000 revolutions per minute, centrifuge the mixture for 4 min at a temperature of 25 °C to separate the oil and water. Once the oil and water have been separated, retrieve the upper layer, which contains the essential oil. A suitable quantity of anhydrous sodium sulfate was applied to eliminate a minor quantity of water. The GC-MS conditions for FCEO were as follows: essential oil was diluted to a volume of 1 mL using ethyl acetate and then passed through a microporous membrane with a pore size of 0.45 μm. The Agilent HP-5 column is made of 5%-phenyl)-methylpolysiloxane and has dimensions of 30 m × 250 μm × 0.25 μm. The carrier gas used is helium (He), with a flow rate of 1 mL/min. The planned temperature increase was configured as follows: The column temperature was initially set at 40 °C and held for 5 min. It was then increased to 160 °C at a rate of 10 °C/min and maintained for 2 min. Afterward, the temperature was further increased to 200 °C at a rate of 5 °C/min and held for 2 min. Finally, the partitioning rate was raised to 240 °C at a rate of 2 °C/min and maintained for 5 min. The split ratio was 100: 1. The injection volume was 1 μL. The mass spectrometry parameters included EI ionization with an electron energy of 70 eV and an ion source temperature of 220 °C, and the scanning mode was set to full-scanning.

The compounds CXEO and FCEO were combined in equal proportions and subjected to analysis using GC-MS. The GC-MS conditions for the combined essential oil of CX and FC require diluting it to a volume of 1 mL using ethyl acetate and subsequently filtering it through a microporous membrane with a pore size of 0.45 μm. The Agilent HP-5 column consists of 5%-phenyl)-methylpolysiloxane and has a length of 30 m, a diameter of 250 μm, and a film thickness of 0.25 μm. The carrier gas employed is helium (He), and it flows at a rate of 1 mL/min. The specified ramp-up settings were as follows: The column temperature was originally adjusted to 40 °C and held constant for a duration of 1 min. The temperature was initially raised to 180 °C at a rate of 5 °C/min. It was then maintained at this temperature for 1 min before being further increased to 220 °C at a rate of 2 °C/min. The final temperature was then sustained for 5 min. The splitting ratio employed was 100: 1, with an injection volume of 1 μL. The mass spectrometry settings consisted of electron ionization (EI) with an electron energy of 70 eV. The ion source temperature was set at 220 °C, and the scanning mode employed was full-scanning.

Identification method: The retention time (RT) of each compound was converted to a retention index (RI) for reference using GC-MS. The identification of the composition of the observed chemicals was accomplished by comparing them with the published NIST 14 spectral library.

### 2.6. Network Pharmacology Research

The GC-MS measurement and analysis results were used to retrieve the components’ structures from the Pubchem database. The structures were saved as sdf files and imported into the SwissTargetPrediction and Pharmmapper databases to predict the targets. The predicted results from the SwissTargetPrediction database were considered as the targets, based on their probability. The targets in the SwissTargetPrediction database and the target retrieval results in the Pharmmapper database, with a probability greater than 0, were adjusted using the Uniprot database. The database retrieval results for each component were then merged and any duplicate targets were eliminated, resulting in the final target for each component. We conducted a keyword search using “insomnia” in the GeneCards, TTD, and OMIM databases to identify disease targets. Subsequently, we compiled and summarized the database results, eliminating any duplicate entries. The findings were condensed, and the duplicate entries were eliminated. The Wayne diagrams of chemical composition targets and disease targets were generated using VENNY 2.1.0, and the overlapping targets were identified. We generated “Network” and “Type” files based on the components, intersection targets, and diseases. Using Cytoscape 3.9.1, we constructed a network diagram representing the relationship between constituents and intersection targets. We then performed topological analysis to extract important data from the network diagram.

The STRING database was used to analyze the protein interactions of overlapping targets. To obscure the unbound targets, the interaction factor was adjusted to 0.4. Subsequently, the network information was exported to a file in the “TSV” format. Open the “TSV” file using Cytoscape 3.9.1 to create the protein–protein interaction (PPI) network. Perform topology analysis to obtain data related to the network diagram. The David database was utilized for conducting enrichment analysis on overlapping targets. GO functional analysis primarily served to characterize the functionalities of gene targets, encompassing biological processes, cellular components, and molecular functions. KEGG enrichment analysis identifies the important pathways that are enriched by the targets for the pharmacological therapy of insomnia.

### 2.7. Western Blot Assay

The mouse brain tissues were collected and subjected to homogenization and lysis. Total protein was extracted from the tissues, and the protein concentration was determined using the bicinchoninic acid method. Each group of proteins was then separated using 10% sodium dodecyl sulfate–polyacrylamide gel electrophoresis electrophoresis. After 30 min of electrotransfer to the PVDF membrane, remove the membrane and rinse it in Tris-buffered saline with tween-20 (TBS-T) for 5 min, and then block with blocking solution for 1 h (TBS-T buffer containing 5% skimmed milk powder). After incubation at room temperature, the blocking solution was discarded, and the primary antibodies JAK2, P-JAK2, STAT3, P-STAT3, and SOCS3 (1:1000) were added. After incubation at 4 °C overnight, TBS-T was washed three times for 5 min each. Add the secondary antibody (1:1000) and incubate it for 1 h, and then wash TBST three times for 5 min each time. The results were visualized by the chemiluminescence (ECL) Western Blotting Detection System. Original figures can be found in Supplementary Materials. 

### 2.8. Statistical Analysis

The results were described as the mean ± standard deviation (SD). SPSS 21.0 software was used for statistical analysis, the single-factor ANOVA method was used for analysis, the LSD method was used for homogeneity of variance, the GamesHowell method was used for heterogeneity of variance, and *p* < 0.05 was taken as the level of the test.

## 3. Results

### 3.1. Optimization of the Preparation Conditions for CXEO-FCEO-CNF

Three key factors that affect the particle size (nm), EE1 (%), and EE2 (%) were examined through one-way experiments involving ultrasonic power (w), the NaClO dosage (mL), and the ratio of essential oils to nanofibers (%). EE1 represents the encapsulation rate for CXEO, while EE2 represents the encapsulation rate for FCEO. Seventeen experimental optimizations were conducted using a central composite design to minimize the particle size (Y1, measured in nanometers) of CXEO-FCEO-CNF while maximizing the EE1 (Y2, measured in percentage) and EE2 (Y3, measured in percentage) responses. Multiple regression analysis was then used to obtain second-order polynomial equations for the response and test variables. The model equations for the particle size (Y1), EE1 (Y2), and EE2 (Y3) are as follows:

Y1 = 106.12 − 6.94 × A + 1.04 × B + 4.52 × C − 7.33 × AB − 1.44 × AC + 0.3825 × BC − 21.15 × A^2^ − 12.70 × B^2^ − 9.99 × C^2^ (1)

Y2 = 42.88 + 3.36 × A + 4.13 × B + 2.85 × C − 2.10 × AB + 2.51 × AC + 0.2275 × BC − 7.76 × A^2^ − 5.85 × B^2^ − 5.26 × C^2^ (2)

Y3 = 42.78 + 6.32 × A + 5.17 × B + 2.32 × C − 3.18 × AB + 0.8275 × AC − 1.08 × BC − 5.14 × A^2^ − 4.36 × B^2^ − 3.92 × C^2^ (3)

**Figure 3:**
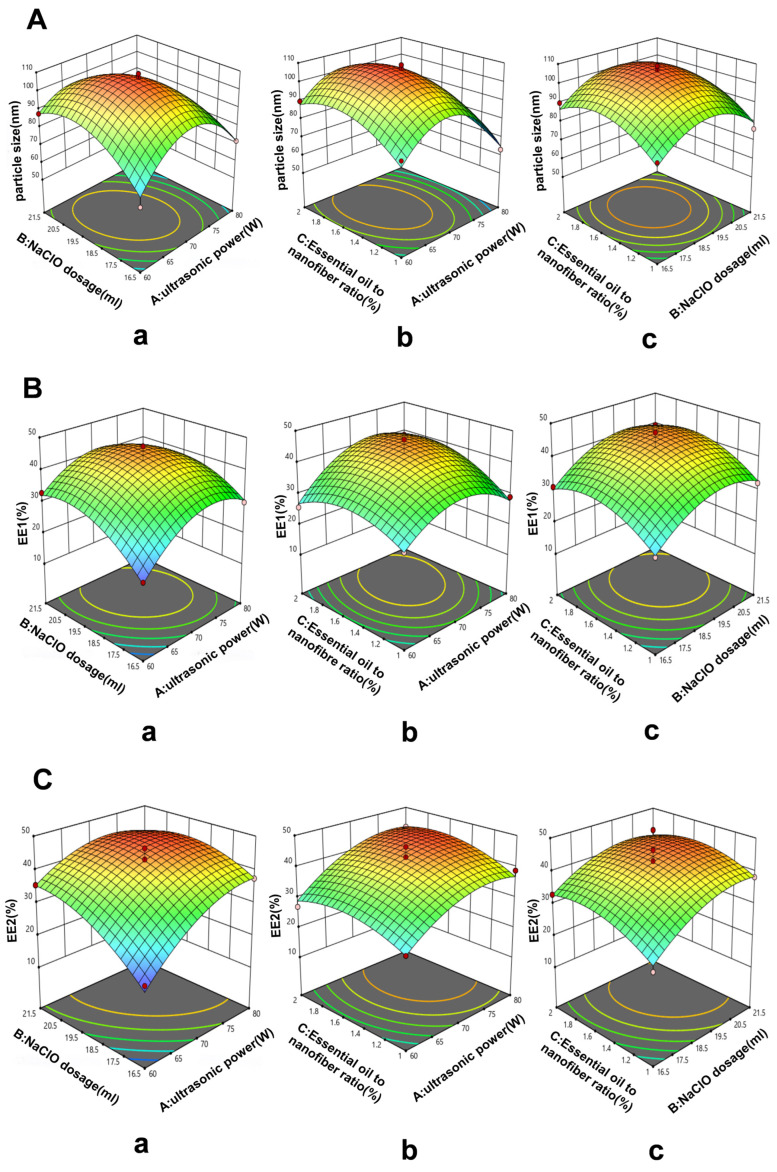
Three-dimensional response surface diagrams. (**A**) Response surface diagrams of particle size. (**B**) Response surface diagrams of EE1. (**C**) Response surface diagrams of EE2. (a) NaClO dosage to ultrasonic power. (b) Essential-oil-to-nanofiber ratio to ultrasonic power. (c) Essential-oil-to-nanofiber ratio to NaClO dosage. The gradual change from the green region part to the red region part means that the particle size and encapsulation efficiency gradually increases, and the fixed point in the red region is the maximum particle size and encapsulation efficiency in the experimental simulation.

The quadratic regression model was assessed using the coefficient of determination (R^2^) and the *p*-value of the ANOVA test. The R^2^ for the particle size regression model was 0.9629, for the EE1 regression model, it was 0.9633, and for the EE2 regression model, it was 0.9580. These high R^2^ values indicate that the models were very significant and predictable within the range of experimental variables. The ANOVA analysis revealed that the numerical models for particle size, EE1, and EE2 had a statistically significant relationship. Hence, the model can be utilized to forecast the most advantageous parameters for the formulation of CXEO-FCEO-CNF. Figure 3 displays the 3D response surface plots for several interaction types and their respective variables. Ultimately, the preparation parameters that yielded the best results were an ultrasonic power of 70 W, a NaClO dosage of 19.87 mL, and an essential-oil-to-nanofiber ratio of 1.7%. The accuracy of the projected model was confirmed by producing three sets of nanofibers using the optimal prescription procedure. The measured particle size of CXEO-FCEO-CNF was 103.19 nm, with an EE1 value of 44.50% and an EE2 value of 46.15%. The deviations between the predicted and experimental values were 3.19%, 0.45%, and 1.41% (Table 2), respectively, suggesting that the binomial equation was well fitted with high confidence.

### 3.2. Characterization of CXEO-FCEO-CNF

**Figure 4:**
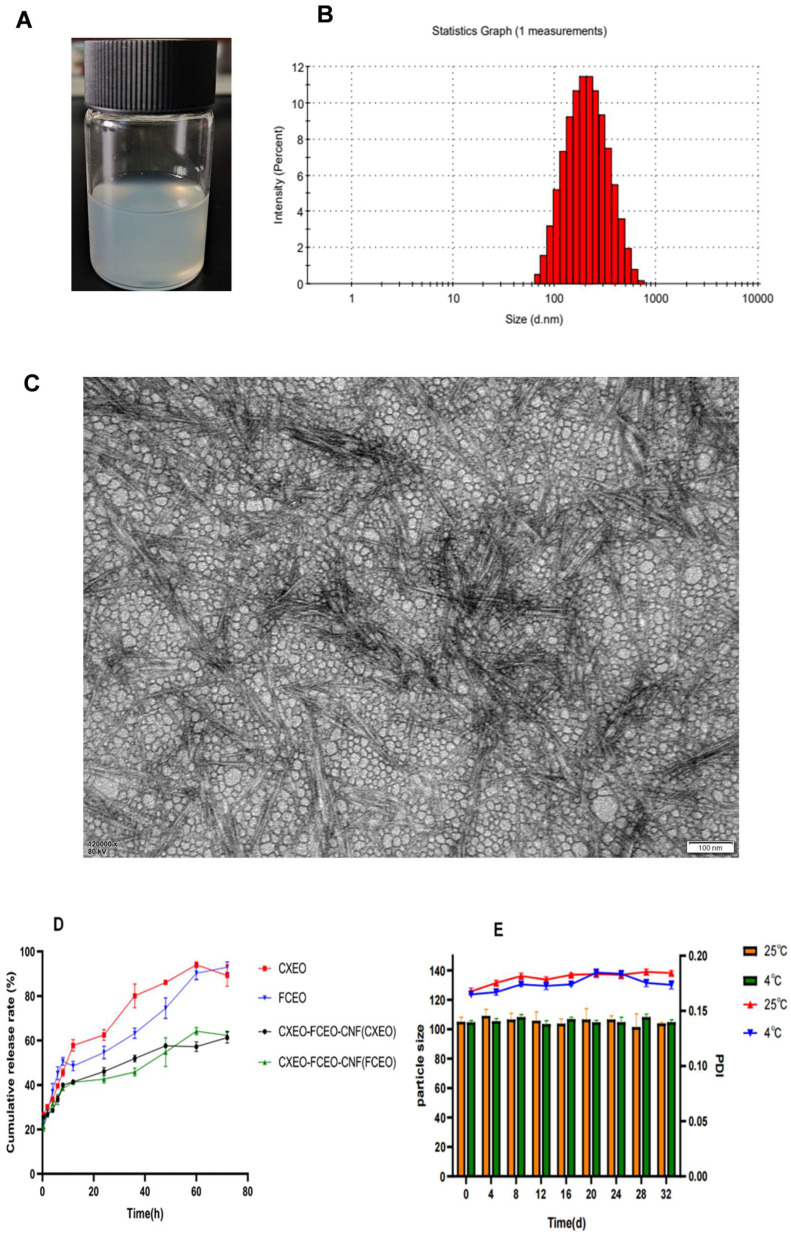
Characterization of CXEO-FCEO-CNF. (**A**) Appearance of CXEO-FCEO-CNF. (**B**) Size distribution of CXEO-FCEO-CNF. (**C**) TEM photograph of CXEO-FCEO-CNF. (**D**) In vitro release plots of CXEO, FCEO, and CXEO-FCEO-CNF. (**E**) Graph of the results of the stability examination of CXEO-FCEO-CNF.

Figure 4A–C display the findings of the analysis of the appearance, particle size, and morphology of CXEO-FCEO-CNF. The nanofibers had an average particle size of 103.19 ± 1.64 nm (*n* = 3), with a PDI of 0.192. They exhibited a narrow particle size distribution and excellent dispersion. The results of the transmission electron microscopy revealed that the nanofibers had a rod-like morphology, making them suitable for drug delivery within the brain.

**Figure 5:**
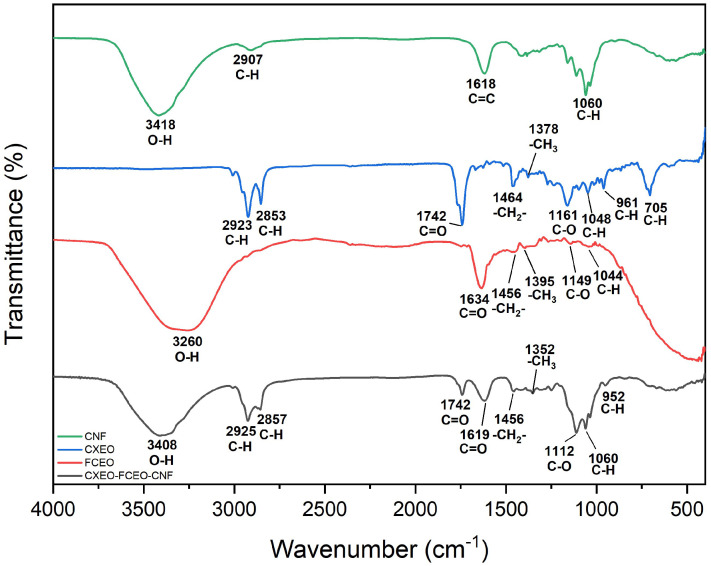
FT-IR result graph.

Figure 5 displays the findings of the infrared spectroscopy. The CNF infrared spectrogram shows O-H stretching vibration peaks at 3418 cm^−1^, C-H stretching vibration peaks at 2907 cm^−1^, C=C double bond stretching vibration peaks at 1618 cm^−1^, and C-H in-plane bending vibration peaks at 1060 cm^−1^; the CXEO infrared spectrograms show C-H stretching vibration peaks at 2923 and 2853 cm^−1^, the C=O at 1742 cm^−1^, -CH_2_- bending vibration at 1464 cm^−1^, -CH_3_ bending vibration at 1378 cm^−1^, C-O bending vibration at 1161 cm^−1^, C-H in-plane bending vibration at 1048 cm^−1^, and C-H out-of-plane bending vibration at 961 and 705 cm^−1^; and the FCEO IR spectra showed an O-H stretching vibration peak at 3260 cm^−1^, an ester group C=O stretching vibration peak at 1634 cm^−1^, a -CH_2_- bending vibration peak at 1456 cm^−1^, a -CH_3_ bending vibration peak at 1395 cm^−1^, a C-O stretching vibration peak at 1149 cm^−1^, and a C-H in-plane bending vibration peak at 1044 cm^−1^. CXEO-FCEO-CNF infrared spectra show that the infrared absorption peaks of all three substances are reflected, indicating the success of the composite. Additionally, 3408 cm^−1^ is the O-H stretching vibration peak on CNF and FCEO, 2923 cm^−1^ and 2853 cm^−1^ are the C-H stretching vibration peaks on CNF and CXEO, 1742 cm^−1^ is the carbonyl C=O stretching vibration peak on CXEO, and 1634 cm^−1^ is the carbonyl C=O stretching vibration peak on CXEO. Additionally, 1634 cm^−1^ is the telescopic vibrational peak of the ester group C=O in FCEO, at 1456 cm^−1^ is the bending vibration absorption peak of -CH2- on CXEO-FCEO, the bending vibrational peak of -CH_3_- on CXEO-FCEO is 1378 cm^−1^, 1112 cm^−1^ is the absorption peak of the stretching vibration of C-O on CXEO-FCEO, 1060 cm^−1^ is the in-plane bending vibrational peak of C-H on CXEO-FCEO-CNF, and 952 cm^−1^ is the out-of-plane bending vibrational peak of C-H.

### 3.3. In Vitro Release and Stability Study

The results of CXEO, FCEO, and CXEO-FCEO-CNF released in vitro within 72 h are shown in Figure 4D. The CXEO solution and FCEO solution exhibited a swift release pattern after 12 h, with release rates of 57.80% and 48.52%, respectively. On the other hand, the CXEO-FCEO-CNF group exhibited a distinct pattern characterized by two phases: a period of quick release followed by a period of delayed release, resulting in a gradual and sustained release effect. The nanofiber formulations exhibited a quick release of CXEO and FCEO within the first 36 h, followed by a subsequent slow release phase characterized by a gradual increase in cumulative release over the period of 48–72 h. The nanofiber compositions exhibited an accelerated release of CXEO and FCEO within the first 36 h, followed by a subsequent period of gradual release. This could be attributed to the dominance of free drug release in the initial stage and the gradual dispersion of the encapsulated dose during the slow release period in the nanofiber formulations.

Figure 4E displays the outcomes of the stability experiments. The CXEO-FCEO-CNF sample exhibited no alteration in color, no formation of solid particles, even distribution, and minimal variations in particle size and PDI over a 30-day storage period at a temperature of 4 °C. The findings indicated that the nanofiber formulation exhibited excellent storage stability.

### 3.4. Effects of CXEO-FCEO-CNF on Body Weight, Body Temperature, Food Intake, and Grip Strength in Insomniac Mice

**Figure 6:**
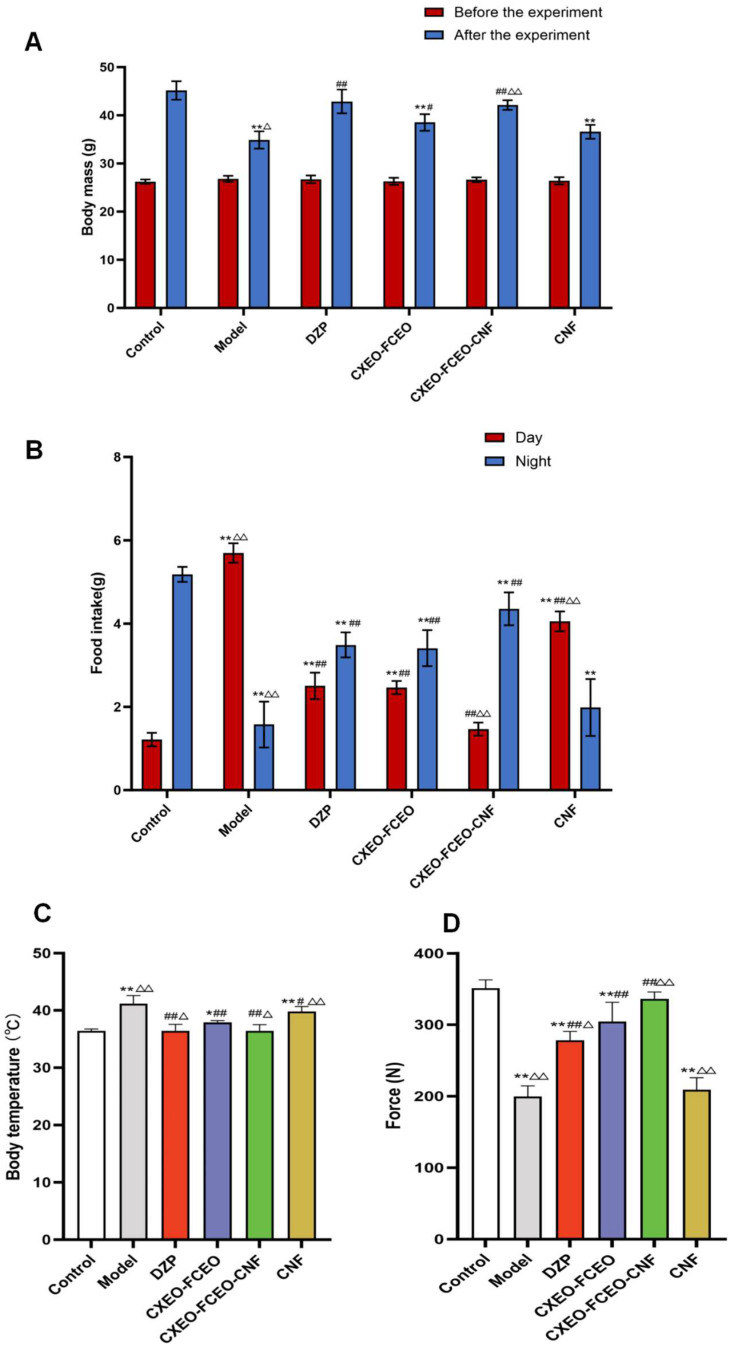
Comparison of body weight, food intake, body temperature, and grip strength of mice in each group. (**A**) Changes in the body weight of mice in each group before and after the experiment. (**B**) Changes in the daytime and nighttime food intake of mice in each group. (**C**) Changes in the body temperature of mice in each group. (**D**) Changes in the grip strength of mice in each group. Data are expressed as the mean ± SD. * refers to a significant difference when compared with the control group, * indicates *p* < 0.05, ** indicates *p* < 0.01, ^#^ refers to a significant difference when compared with the model group, ^#^ indicates *p* < 0.05, ^##^ indicates *p* < 0.01, ^Δ^ refers to a significant difference when compared with the CXEO-FCEO group, ^Δ^ indicates *p* < 0.05, and ^ΔΔ^ indicates *p* < 0.01.

In order to assess the impact of CXEO-FCEO-CNF on the body mass of mice with insomnia, the alterations in the body weight of the mice were measured and documented prior to and following the experiment. As depicted in Figure 6A, there was minimal disparity in the body mass of the mice in each group before the commencement of the investigation. However, after the conclusion of the experiment, the body mass of the model group exhibited a substantial reduction in comparison to that of the blank control group (*p* < 0.01). The DZP, CXEO-FCEO, and CXEO-FCEO-CNF groups exhibited statistically significant increases in body mass compared to the model group (*p* < 0.05, 0.01). Specifically, the positive control and CXEO-FCEO-CNF groups had a more rapid rise in body mass than the CXEO-FCEO group. Notably, the CXEO-FCEO-CNF group exhibited considerably greater progress in weight compared to the CXEO-FCEO group (*p* < 0.01).

In order to assess the impact of CXEO-FCEO-CNF on the eating habits of mice with insomnia, we measured and documented any changes in their food consumption during the day and night in each experimental group. Figure 6B demonstrates that the daily food consumption of normal mice was notably lower during the daytime compared to that during the nighttime. This is due to the mice being in a sleeping state during the day and consuming less food. On the other hand, the model group of mice exhibited a significantly higher intake of food during the daytime compared to the nighttime, indicating a disrupted sleeping pattern in these mice. The DZP, CXEO-FCEO, and CXEO-FCEO-CNF groups exhibited a noteworthy reduction in daytime feeding (*p* < 0.01) and a notable rise in nocturnal feeding (*p* < 0.05, 0.01) when compared to the model group. These findings indicate that the sleep patterns of the mice restored to a normal state.

In order to examine the impact of CXEO-FCEO-CNF on the central body temperature of mice suffering from insomnia, we conducted temperature measurements on the mice. The body temperatures of mice in the model and blank CNF groups were considerably higher (*p* < 0.01) compared to those of the control group. In comparison to the model group, the DZP group, the CXEO-FCEO group, and the CXEO-FCEO-CNF group all exhibited a substantial decrease in the back temperature of mice (*p* < 0.01) (Figure 6C). Through the grip strength test, we observed a substantial decrease in grip strength in the mice in the model group (*p* < 0.01). However, after administering CXEO-FCEO-CNF, the mice in the CXEO-FCEO-CNF group showed a significant restoration of grip strength (*p* < 0.01). These findings demonstrate that CXEO-FCEO-CNF has the potential to alleviate core body temperature and fatigue symptoms in mice with insomnia, as shown in Figure 6D.

### 3.5. Sedative Effect of CXEO-FCEO-CNF on Insomniac Mice

**Figure 7:**
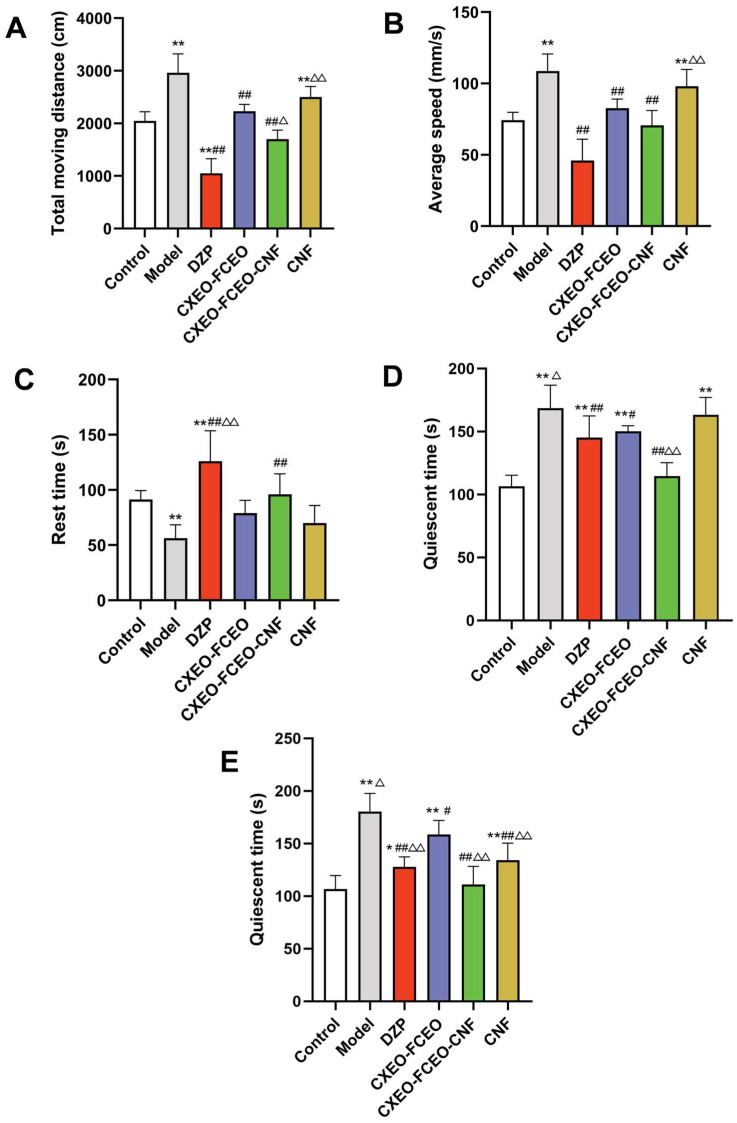
Diagram of behavioral experiments in mice. (**A**) Distance traveled in OFT. (**B**) Average speed of OFT. (**C**) Rest time of OFT. (**D**) Quiescent time in FST. (**E**) Quiescent time in TST. Data are expressed as the mean ± SD. * refers to a significant difference when compared with the control group, * indicates *p* < 0.05, ** indicates *p* < 0.01, ^#^ refers to a significant difference when compared with the model group, ^#^ indicates *p* < 0.05, ^##^ indicates *p* < 0.01, ^Δ^ refers to a significant difference when compared with the CXEO-FCEO group, ^Δ^ indicates *p* < 0.05, and ^ΔΔ^ indicates *p* < 0.01.

OFT was conducted to assess the impact of CXEO-FCEO-CNF on the anxiety levels and exploratory behavior of animals suffering from insomnia. Furthermore, the immobile state of mice in the FST and TST over a certain duration may correlate with the level of hopelessness experienced by the mice. The findings from OFT are displayed in Figure 7. The motor ability of mice with insomnia in the model group was significantly greater than that of the control group (*p* < 0.01). The locomotor activity of mice in the CXEO-FCEO group, CXEO-FCEO-CNF group, and DZP group was considerably reduced (*p* < 0.01) compared to that of the model group, as shown in Figure 7A–C. The CXEO-FCEO-CNF group exhibited a notably superior performance compared to the CXEO-FCEO group in decreasing the overall distance covered by the mice (*p* < 0.05). The impact of CXEO-FCEO-CNF on the FST and TST in mice with insomnia is illustrated in Figure 7D,E. The duration of rest during the FST and TST was considerably longer in the group of mice with insomnia compared to that in the control group (*p* < 0.01). In comparison to the model group, the DZP group, CXEO-FCEO group, and CXEO-FCEO-CNF group were able to significantly reduce the resting time (*p* < 0.05, 0.01). Furthermore, the CXEO-FCEO-CNF group demonstrated a significantly greater effect in reducing the resting time compared to the CXEO-FCEO group (*p* < 0.01). The sedative properties of CXEO-FCEO-CNF are believed to enhance relaxation and reduce tension and anxiety-like behavior in mice with insomnia.

### 3.6. Onset and Duration of Sleep in Pentobarbital-Induced Sleep Test

**Figure 8:**
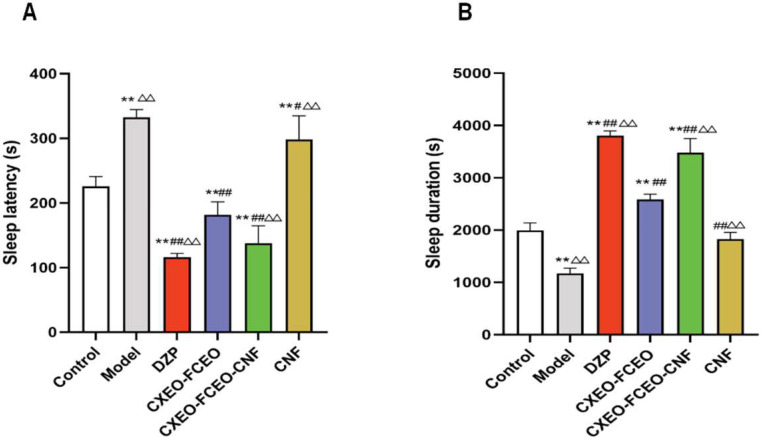
Diagram of sodium pentobarbital-induced sleep experiment. (**A**) Sleep latency. (**B**) Sleep duration. Data are expressed as the mean ± SD. * refers to a significant difference when compared with the control group, ** indicates *p* < 0.01, ^#^ refers to a significant difference when compared with the model group, ^#^ indicates *p* < 0.05, ^##^ indicates *p* < 0.01, ^Δ^ refers to a significant difference when compared with the CXEO-FCEO group, and ^ΔΔ^ indicates *p* < 0.01.

The effects of CXEO-FCEO-CNF on sleep latency and sleep duration in mice induced by a hypnotic dose of sodium pentobarbital (55 mg/kg) are shown in Figure 8. Compared with the control group, the sleep latency of insomniac mice in the model group was significantly lengthened (*p* < 0.01) and the sleep duration was significantly shortened (*p* < 0.01). Compared with the model group, the CXEO-FCEO-CNF group, CXEO-FCEO group, and DZP group all significantly enhanced the hypnotic effect of sodium pentobarbital by lengthening the sleep duration (*p* < 0.01) and shortening the sleep latency (*p* < 0.01) of insomniac mice. In particular, the CXEO-FCEO-CNF group was more able to prolong the sleep duration and shorten the sleep latency compared with the CXEO-FCEO group, suggesting that CXEO-FCEO-CNF has a better sedative-hypnotic effect on insomnia model mice.

### 3.7. The Effect of CXEO-FCEO-CNF on SOD Activity and MDA Content

**Figure 9:**
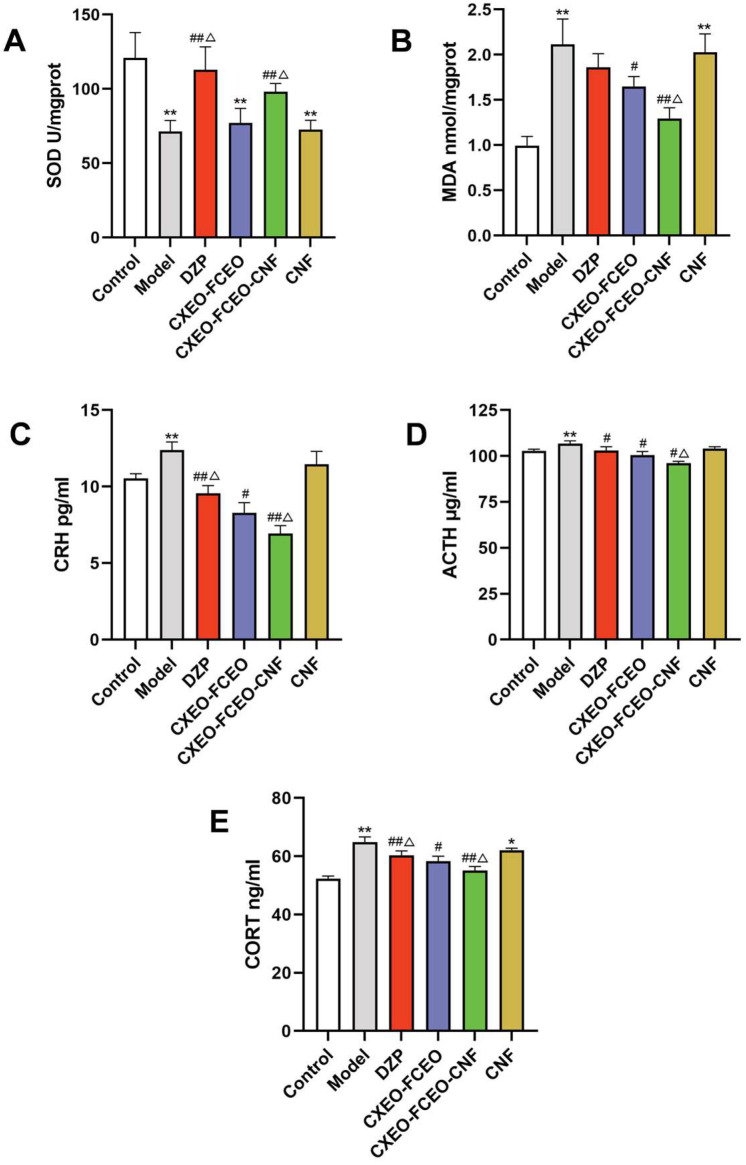
Oxidative stress index test and serum biochemical index test. (**A**) SOD activity. (**B**) MDA level. (**C**) CRH level. (**D**) ACTH level. (**E**) CORT level. Data were expressed as the mean ± SD. * refers to a significant difference when compared with the control group, * indicates *p* < 0.05, ** indicates *p* < 0.01, ^#^ refers to a significant difference when compared with the model group, ^#^ indicates *p* < 0.05, ^##^ indicates *p* < 0.01, ^Δ^ refers to a significant difference when compared with the CXEO-FCEO group, ^Δ^ indicates *p* < 0.05.

Insomnia has been linked to oxidative stress based on available evidence [27]. The current study found that sleeplessness led to reduced activity of SOD, an enzyme that acts as an antioxidant, indicating a compromised antioxidant defense mechanism. Nevertheless, the CXEO-FCEO-CNF group effectively improved the decrease in SOD activity in the brain (*p* < 0.01, Figure 9A). The elevated levels of MDA observed in the brains of mice with insomnia suggest that the membrane has experienced oxidative damage, as shown in Figure 9B. The administration of CXEO-FCEO-CNF and CXEO-FCEO effectively reduced the increase in MDA levels compared to the model group (*p* < 0.05, 0.01). The inhibitory impact of CXEO-FCEO-CNF on the formation of MDA was substantially superior to that of CXEO-FCEO (*p* < 0.05).

### 3.8. The Influence of CXEO-FCEO-CNF on the HPA Axis

To assess the level of HPA axis-related hormone secretion, we used ELISA to detect serum hormone levels. As shown in Figure 9C–E, the levels of CRH, ACTH, and CORT were significantly elevated in mice in the model group compared with the control group (*p* < 0.05). Compared with the model group, CXEO-FCEO-CNF and CXEO-FCEO significantly decreased the levels of CRH, ACTH, and CORT in mice (*p* < 0.05, 0.01). The results suggest that CXEO-FCEO-CNF can effectively regulate the secretion of HPA axis-related hormones, which may be one of the reasons for its alteration of sleep in mice.

### 3.9. The Effect of CXEO-FCEO-CNF on 5-HT, GABA, DA, and NE Neurotransmitters

**Figure 10:**
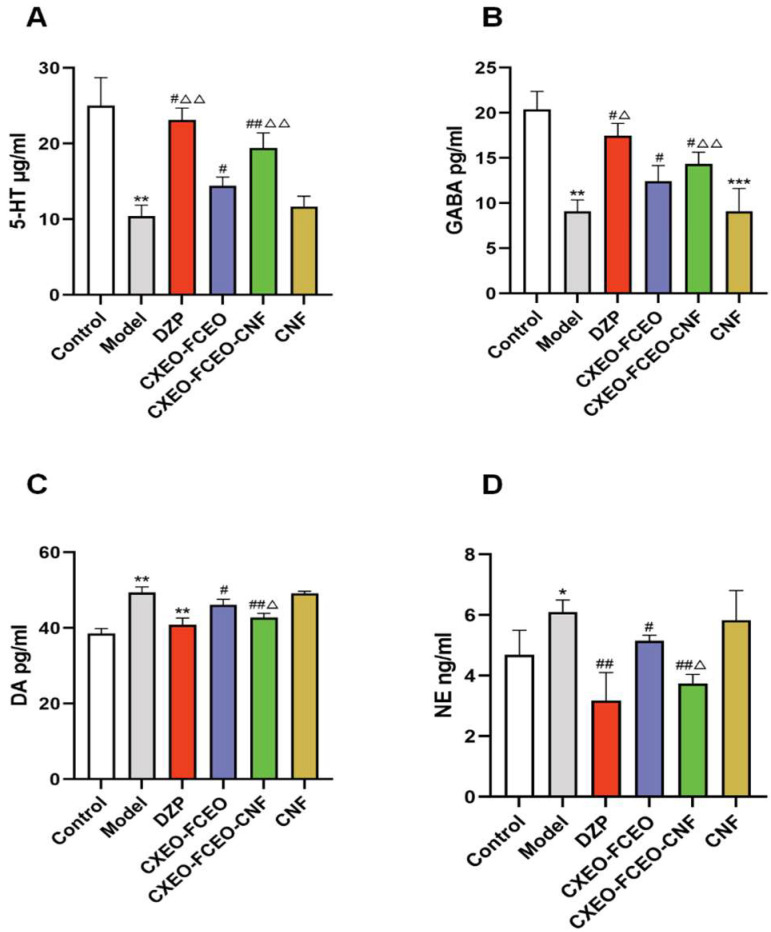
Neurotransmitter content assay. (**A**) 5-HT levels. (**B**) GABA levels. (**C**) DA level. (**D**) NE levels. Data are expressed as the mean ± SD. * refers to a significant difference when compared with the control group, * indicates *p* < 0.05, ** indicates *p* < 0.01, *** indicates *p* < 0.001, ^#^ refers to a significant difference when compared with the model group, ^#^ indicates *p* < 0.05, ^##^ indicates *p* < 0.01, ^Δ^ refers to a significant difference when compared with the CXEO-FCEO group, ^Δ^ indicates *p* < 0.05, and ^ΔΔ^ indicates *p* < 0.01.

Figure 10 demonstrates the detection of the impact of CXEO-FCEO-CNF on neurotransmitters in mice with insomnia by measuring the levels of 5-HT, GABA, DA, and NE. In comparison to the control group, the levels of 5-HT and GABA were dramatically reduced (*p* < 0.01), while the concentrations of DA and NE were significantly elevated (*p* < 0.05, 0.01) in the model group of mice. In comparison to the model group, mice treated with CXEO-FCEO-CNF, CXEO-FCEO, and DZP exhibited significant increases in the synthesis of 5-HT and GABA (*p* < 0.05, 0.01). Furthermore, when compared to the model group, both CXEO-FCEO-CNF and CXEO-FCEO significantly reduced the production of DA (*p* < 0.05, 0.01), and CXEO-FCEO-CNF, CXEO-FCEO, and DZP also decreased NE production (*p* < 0.05, 0.01). Significantly, CXEO-FCEO-CNF demonstrated a greater increase in 5-HT and GABA compared to CXEO-FCEO and a greater drop in DA and NE compared to CXEO-FCEO (*p* < 0.05, 0.01). CXEO-FCEO-CNF is hypothesized to influence the sleep patterns of insomniac mice by modulating the concentrations of neurotransmitters such as 5-HT, GABA, DA, and NE.

### 3.10. Security Assessment

**Figure 11:**
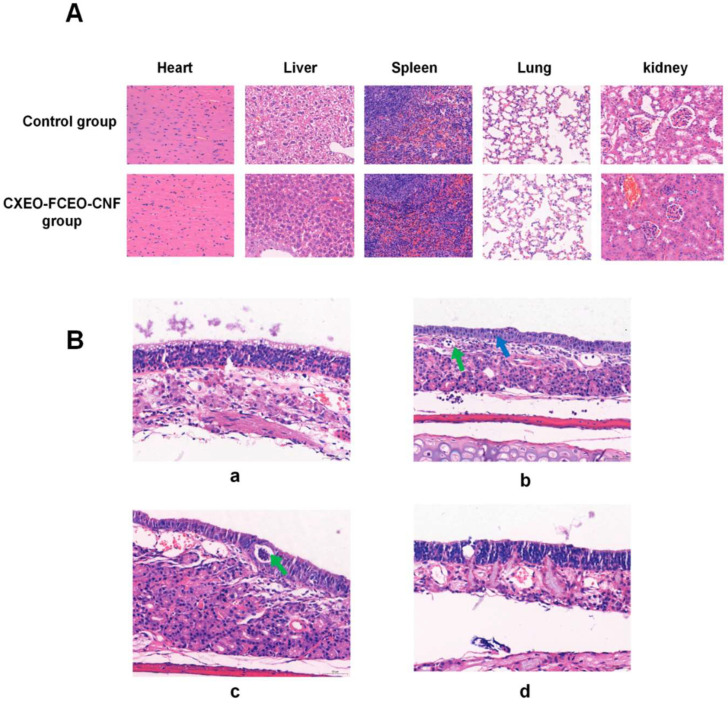
Safety evaluation. (**A**) Tissue safety evaluation after drug inhalation via the nose (×400). (**B**) HE staining images of the nasal mucosa of mice in each group (×400). (**a**) Saline group; (**b**) Sodium deoxycholate group; (**c**) CXEO-FCEO solution group; (**d**) CXEO-FCEO-CNF group. Green arrows represent inflammatory cell infiltration and blue arrows indicate epithelial cell proliferation.

After 28 days of CXEO-FCEO-CNF administration, the HE staining results of the heart, liver, spleen, lungs, and kidneys of mice are shown in Figure 11A, and all the organs did not show any obvious lesions, suggesting that the nasal inhalation of CXEO-FCEO-CNF solution led to no obvious acute toxicity for the tissues of mice. The effect on the morphology of the nasal mucosa is shown in Figure 11B, the tissue structure of the nasal mucosa of mice in the saline group was relatively intact, and the nasal mucosa of mice showed epithelial cell hyperplasia and inflammatory cell infiltration after nasal administration of 1% sodium deoxycholate. The mucosal epithelial cells in the CXEO-FCEO solution group were more neatly arranged, and the epithelial cells of the nasal mucosa of the mice showed a slight proliferation of the mucosal epithelial cells and infiltration of the inflammatory cells, which indicates that the purified CXEO-FCEO solution may have slight toxicity to the nasal mucosa. No obvious pathological damage was seen in the nasal mucosa of mice in the CXEO-FCEO-CNF group, suggesting that CXEO-FCEO-CNF has no obvious toxicity to the nasal mucosa of mice and that CXEO-FCEO encapsulated in nanofibers can effectively alleviate the stimulation of the nasal mucosa by CXEO-FCEO solution.

### 3.11. GC-MS Analysis of CXEO-FCEO

The GC-MS analysis of CXEO-FCEO was performed, and the CXEO-FCEO composition was inferred and examined by querying the NIST14 database for peaks and combining it with relevant graphical analysis. The peak area normalization technique was used to calculate the relative percentages. The results are shown in Table 3. A total of 23 chemical components were identified in the blend of the two essential oils, among which alpha-Asarone, (E)-methyl isoeugenol, and Senkyunolide were found in higher levels and may be the active ingredients in the treatment of insomnia.

### 3.12. Analysis of Network Pharmacology Results

**Figure 12:**
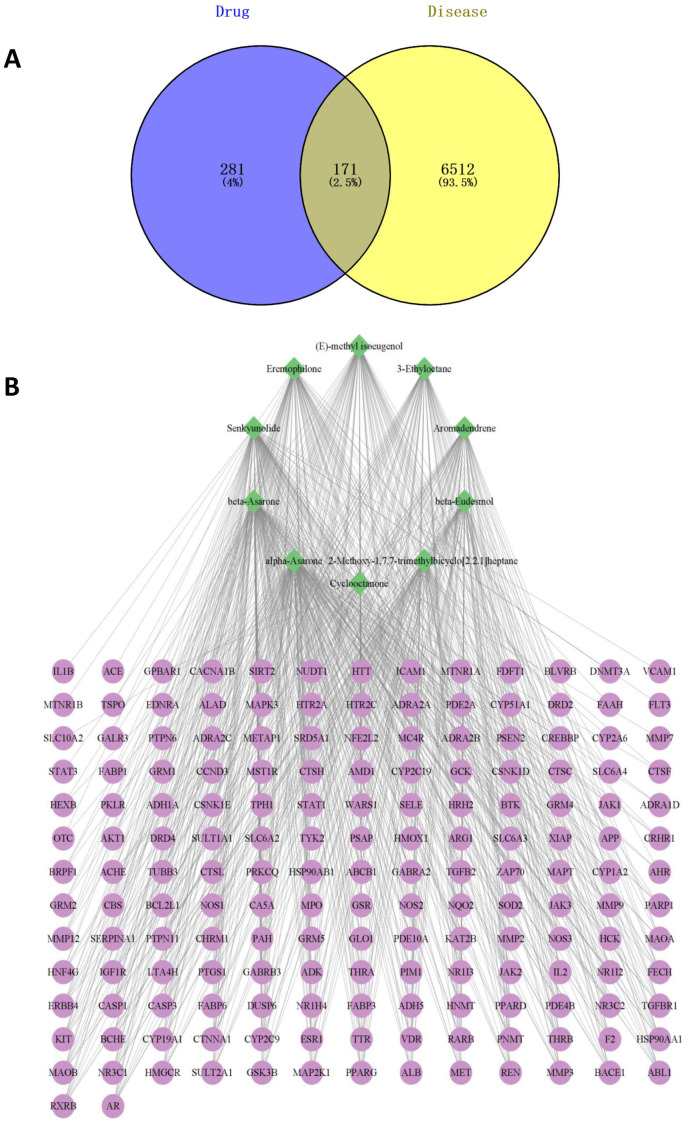
(**A**) Venn diagram of intersecting targets. (**B**) Network diagram of drug component targets.

**Figure 13:**
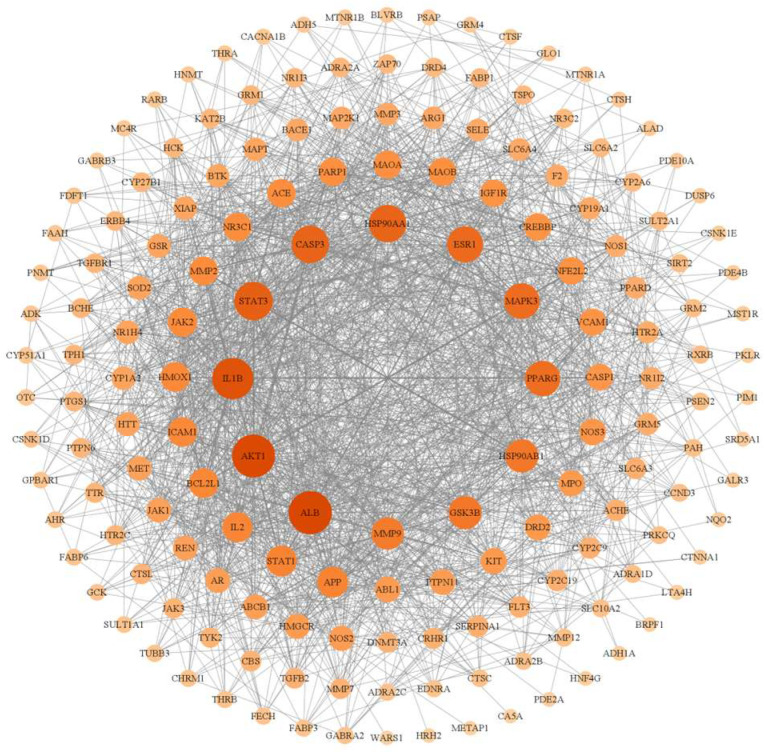
Interaction network of potential targets for the treatment of insomnia.

From the database, we obtained 281 targets corresponding to the ten components of CXEO-FCEO and the 6512 disease targets of insomnia, mapped the component targets with insomnia targets, obtained 171 potential targets for the treatment of insomnia, and used Venny 2.1.0 to draw a Venn diagram to show the results; see Figure 12A. The component-intersection target network diagram is shown in Figure 12B. There were 181 nodes, 10 active ingredients, and 171 target nodes with 680 edges, and the results showed that the first 7 active ingredients with degree values greater than the average (degree value ≥ 4) were mainly alpha-Asarone, (E)-methyl isoeugenol, and so on. Protein interaction analysis of the intersecting targets was carried out using the String database, and Cytoscape 3.9.1 was used to construct the PPI network, as shown in Figure 13. The data related to the network graph were obtained by topology analysis, which showed that there were 65 targets with the degree of freedom, tightness, and centrality of each node of the network exceeding the average value. Taking the node degree value as an evaluation parameter, the larger the node degree value, the more important it is in the PPI network, and it may play an important role in performing biological functions. According to the analysis of the degree of freedom and center tightness, the core targets were obtained as IL-1β, STAT3, MAPK3, etc., among which IL-1β and STAT3 were closely related to the inflammatory response.

**Figure 14:**
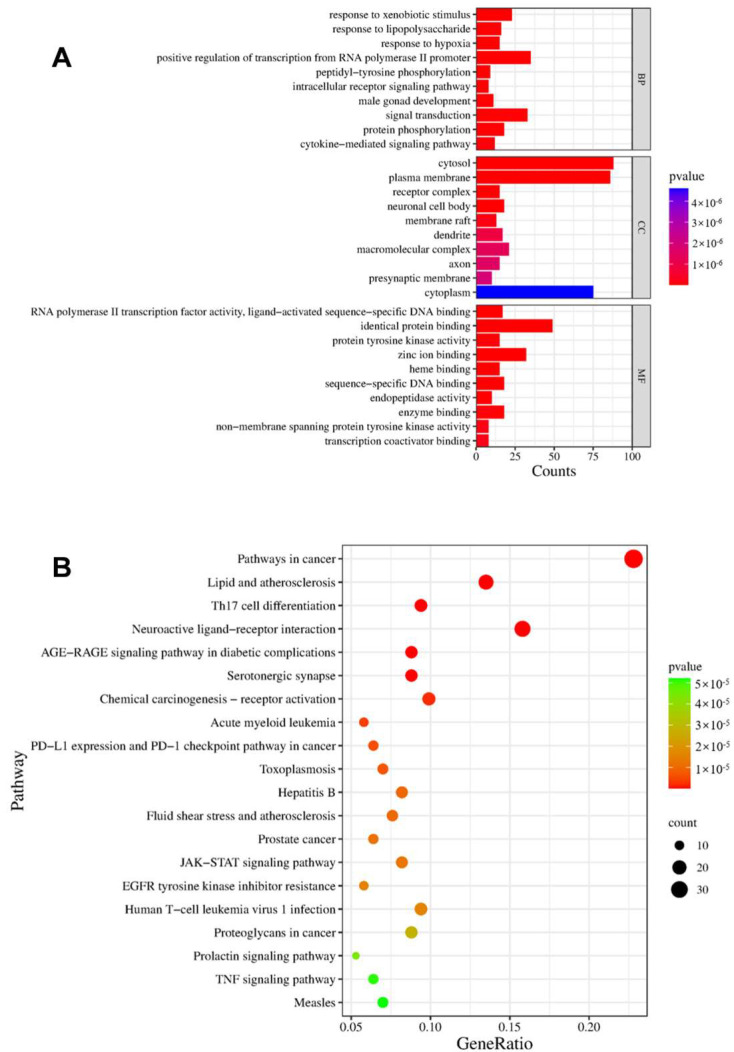
Enrichment analysis. (**A**) GO enrichment analysis. (**B**) KEGG pathway analysis.

The David database platform was used for GO (gene ontology) annotation analysis and Kyoto Encyclopedia of Genes and Genomes (KEGG) pathway enrichment analysis of the obtained intersecting targets, see Figure 14. The main biological processes involved include the response to the xenobiotic stimulus, intracellular receptor, signaling pathway, signal transduction, cytokine-mediated signaling pathway, etc.; they mainly affect cellular components such as the neuronal cell body, receptor complex, axon, presynaptic membrane, etc.; MF annotation mainly involves RNA polymerase II transcription factor activity, ligand-activated sequence-specific DNA binding, protein tyrosine kinase activity, etc.; KEGG analysis involves the pathways shown in Figure 14B, including pathways in cancer, lipid and atherosclerosis, neuroactive ligand–receptor interaction, the JAK-STAT signaling pathway, the TNF signaling pathway, and so on. Therefore, in this study, the JAK-STAT signaling pathway was selected for further validation based on the results of the KEGG analysis of network pharmacology to investigate the potential mechanism of CXEO-FCEO-CNF in the treatment of insomnia.

### 3.13. CXEO-FCEO-CNF Affects P-STAT3, P-JAK2, and SOCS3 Expression

**Figure 15:**
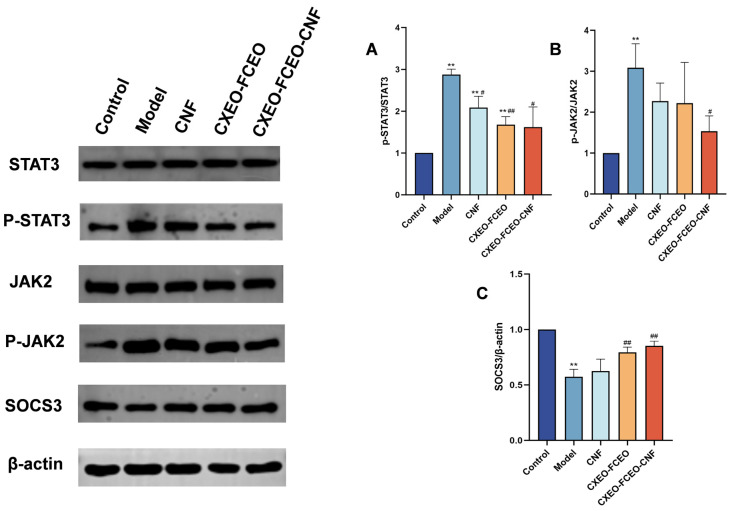
Graph of WB results. (**A**) Expression of P-STAT3 and STAT3. (**B**) Expression of P-JAK2 and JAK2. (**C**) Expression of SOCS3. Data were expressed as the mean ± SD. * refers to a significant difference when compared with the control group, ** indicates *p* < 0.01; ^#^ refers to a significant difference when compared with the model group, ^#^ indicates *p* < 0.05, ^##^ indicates *p* < 0.01 (for the original Western Blot Images, see the supplementary materials).

From the expression levels of hypothalamic P-STAT3 protein, P-JAK2, and SOCS3 protein (Figure 15), compared with the control group, the expression levels of P-STAT3 protein and P-JAK2 protein in the model group of mice were significantly higher (*p* < 0.01), and those of SOCS3 protein were significantly lower; compared with the model group, the CXEO-FCEO-CNF group of mice exhibited considerably reduced levels of P-STAT3 and P-JAK2 proteins compared to the model group. Additionally, the CXEO-FCEO-CNF group showed significantly elevated levels of SOCS3 protein (*p* < 0.05, 0.01).

## 4. Discussion

Insomnia increases the risk of heart disease, high blood pressure, anxiety, and depression [27,28,29]. Ligusticum chuanxiong and Finger citron are commonly used in the treatment of insomnia, and the combination of the extracted essential oils can have synergistic therapeutic effects. However, the essential oils of Ligusticum chuanxiong and Finger citron are poorly stabilized and susceptible to volatilization and oxidative decomposition, resulting in a decrease in the bioactivity of the essential oils or even the production of toxic by-products. Therefore, the selection of appropriate encapsulation materials is essential to improving the stability of essential oils. Currently, the encapsulation of essential oils is mainly achieved by materials such as cyclodextrin inclusion complexes, liposomes, nanoemulsions, and nanofibers [30]. Among them, nanocellulose is a nontoxic, biodegradable, and well-biocompatible nanomaterial; the short rod-like structure of nanocellulose can especially prolong the time of the drug in the nasal cavity. In this study, the CXEO-FCEO-CNF nanofiber system was prepared, and the process was optimized by one-factor investigation and the response surface method to obtain CXEO-FCEO-CNF with a uniform particle size and good stability. Stability experiments found that the particle size and PDI of CXEO-FCEO-CNF did not show any significant change in 30 days. In addition, the nanofiber system has a slow-release effect on the drug and can prolong the duration of action, and the FT-IR results showed that CXEO, FCEO, and CNF were successfully compounded in the same system.

The nasal administration of essential oils has a unique advantage in the treatment of neurological disorders, allowing the drug to bypass the blood–brain barrier and penetrate directly into the olfactory bulb or cerebrospinal fluid. In addition, nasal administration avoids gastrointestinal irritation and hepatic first-pass effects, thus efficiently exerting therapeutic effects. The pathological analysis of nasal mucosa showed that CXEO-FCEO solution had a certain degree of irritation to the nasal mucosa, while the CXEO-FCEO-CNF prepared in this study led to no irritation to the nasal mucosa of mice and did not cause any damage to the heart, liver, spleen, lungs, and kidneys; it could be delivered to the brain by nasal inhalation to exert neuroprotective effects safely.

Clinical studies have shown that insomnia is closely associated with circadian rhythm disruption and altered feeding behavior and that the core body temperature remains low during sleep [31,32,33]. The results of this study found that compared with the model group, the mice in the CXEO-FCEO-CNF group had their circadian rhythms restored to normal, their body weights and food intake increased significantly, and they were able to significantly reduce their body temperatures. In addition, CXEO-FCEO-CNF reduced the resting time of mice in FST and TST, suggesting that it has good sedative, anxiolytic, and depressive effects. On this basis, we investigated the hypnotic effect of CXEO-FCEO-CNF on the experimental behavior of sodium pentobarbital-induced sleep in mice. Interestingly, CXEO-FCEO-CNF significantly shortened sleep latency and increased the total sleep time. Of interest, the sedative-hypnotic effect of CXEO-FCEO-CNF was superior to that of CXEO-FCEO, suggesting that the essential oils of Ligusticum chuanxiong and Finger citron could be better exerted after encapsulation by a nanofiber formulation.

Studies have shown that brain damage is closely linked to levels of oxidative stress [34,35]. Wakefulness due to insomnia requires high neuronal metabolism, which generates large amounts of oxidants and an imbalance in free radical production, and scavenging occurs. Vaccaro et al. found that insomnia can cause the accumulation of reactive oxygen species in the intestinal tract, which can further induce neurological damage or apoptosis or even result in death [27]. Ramanathan et al. found that insomnia for 5–11 days significantly reduced adult rat brain SOD activity, i.e., the body’s antioxidant level is weakened after chronic insomnia [36]. We detected an increase in MDA in sleep-deprived mice, and CXEO-FCEO-CNF significantly reduced MDA levels, attenuated lipid peroxidation, and also increased SOD activity, modulating oxidative stress to improve insomnia, which is consistent with previous findings. In addition, insomnia is triggered by an imbalance between the excitatory and inhibitory functions of the brain, and 5-HT, NE, and DA are in a dynamic equilibrium in vivo; when the brain levels of NE and DA are elevated and the level of 5-HT is reduced, the central nervous system is in an excitatory state [37]. In our study, CXEO-FCEO-CNF was able to reverse the reduction in 5-HT synthesis induced by the tryptophan hydroxylase inhibitor PCPA, significantly decreasing the levels of the excitatory neurotransmitters NE and DA and elevating the level of the inhibitory neurotransmitter GABA. In addition, the perturbation of sleep homeostasis was accompanied by increased HPA axis activity, leading to elevated levels of CRH, ACTH, and CORT, which increase wakefulness [38]. In the present study, we found that HPA axis-related hormone levels were significantly elevated in insomniac mice, but CXEO-FCEO-CNF effectively reversed these changes and exerted a comprehensive intervention on sedative hypnosis.

Sleep deprivation activates microglia and astrocytes to produce IL-6 pro-inflammatory factors, leading to inflammatory cascades and neuronal damage [39]. The JAK/STAT pathway is a key pathway involved in neuroinflammation. The activation of JAK2 by IL-6 promotes the phosphorylation of STAT3, which then induces the inhibitor of cytokine signaling 3 (SOCS3). In this study, the composition of CXEO-FCEO was analyzed by gas chromatography–mass spectrometry, which mainly included alpha-Asarone (21.56%), (E)-methyl isoeugenol (20.17%), and Senkyunolide (4.88%). α-Asarone was reported to have sedative-hypnotic properties, and eugenol and isoeugenol had significant anti-inflammatory, analgesic, and antioxidant properties [40,41]. In addition, It has been shown that Senkyunolide, an active ingredient in Ligusticum chuanxiong, was able to reduce the production of TNF-α, IL-6, IL-1β, and IFN-γ in ROS-induced BV-2 cells [42]. Moreover, Senkyunolide has been studied in the treatment of insomnia. Senkyunolide reduces the excessive release of pro-inflammatory cytokine and decreases the level of phosphorylation of inflammatory signaling proteins [43]. Further, the components in CXEO-FCEO were shown by network pharmacological analysis to act broadly on insomnia-associated STAT3, 1L-1β targets, which may act together on the JAK-STAT pathway. During the validation of the JAK-STAT pathway using the WB approach, it was found that CXEO-FCEO-CNF significantly decreased P-JAK2 levels and P-STAT3 protein expression and increased SOCS3 protein expression. Therefore, our findings suggest that inhibiting neuroinflammation by inhibiting the JAK-STAT pathway may be an effective treatment for insomnia. This study reveals for the first time the mechanism and active substances of CXEO-FCEO -CNF for the treatment of insomnia.

In conclusion, the present study reveals for the first time that the combination of essential oils of Ligusticum chuanxiong and Finger citron can exert sedative-hypnotic effects and that the essential oils of Ligusticum chuanxiong and Finger citron can exert their efficacy better when they are prepared as a composite system of CXEO-FCEO-CNF. Meanwhile, the present study investigated the active substances and pharmacodynamic mechanism associated with the sedative-hypnotic effect of CXEO-FCEO-CNF, which may inhibit the JAK-STAT pathway to suppress neuroinflammation and regulate oxidative stress, neurotransmitter levels, and stress hormone levels in the HPA axis to comprehensively regulate insomnia.

## 5. Conclusions

In the present study, we successfully prepared a new nano-delivery formulation of CXEO-FCEO-CNF for nasal administration, which significantly improved the nasal mucosal irritation and led to the good stability of the essential oils of Ligusticum chuanxiong and Finger citron. Pharmacological studies have shown that CXEO-FCEO-CNF, based on the antioxidant and anti-inflammatory activities of the essential oils, can significantly ameliorate oxidative stress and neuroinflammation in the brain and thus improve sleep disorders and exert sedative-hypnotic effects, especially after encapsulation with nanomaterials, which can be sustained. The potential molecular mechanism is that alpha-Asarone, (E)-methylisoeugenol, and Senkyunolide regulate the JAK-STAT pathway. In summary, the present study promotes the better efficacy of essential oils from herbs by improving the properties of essential oils through nanofiber formulations and facilitates the discovery of novel sedative-hypnotic active substances. Overall, this study is the first to jointly prepare CXEO and FCEO into an essential oil nanofiber composite emulsion system, which may be a promising alternative for the transnasal treatment of insomnia and provide a reference for the development of natural herbal neuroprotective agents.

## Figures and Tables

**Table 1:** Factors and responses in RS-BBD.

Factors	Levels		
	−1	0	1
A (W)	60	70	80
B (mL)	16.5	19	21.5
C (%)	1	1.5	2

**Table 2:** The ratio of predicted values to actual measured values.

Index	Predicted Value	Actual Measured Value	Deviation%
Particle size (nm)	100	103.19	3.19%
EE1 (%)	44.30	44.50	0.45%
EE2 (%)	45.51	46.15	1.41%

**Table 3:** Results of chemical composition analysis of CXEO-FCEO based on GC-MS.

Number	Compounds	LRItab	Identification	Molecular Formula	CAS	Relative (%)	Structural Formula
1	Cineole	1031.8	M, L	C_10_H_18_O	470-82-6	1.38	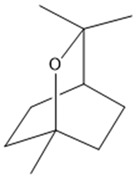
2	p-cymene	1024.3	M, L	C_10_H_14_	99-87-6	0.81	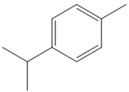
3	Linalool	1099.0	M, L	C_10_H_18_O	78-70-6	1.82	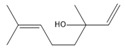
4	Terpinine-4-ol	1177.1	M, L	C_10_H_18_O	562-74-3	0.91	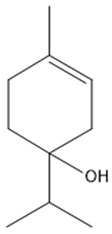
5	Estragole	1195.8	M, L	C_10_H_12_O	140-67-0	2.51	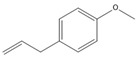
6	Alpha-Terpineol	1189.7	M, L	C_10_H_18_O	98-55-5	1.66	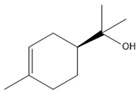
7	Aromadendrene	1440.6	M, L	C_15_H_24_	489-39-4	3.44	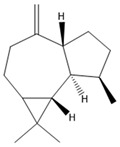
8	Methyleugenol	1401.8	M, L	C_11_H_14_O_2_	93-15-2	2.1	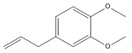
9	Cyclooctanone	985.9	M, L	C_8_H_14_O	502-49-8	4.05	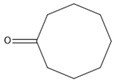
10	(E)-methyl isoeugenol	1401.8	M, L	C_11_H_14_O_2_	93-16-3	20.17	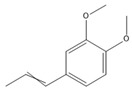
11	4-Methoxyphenylacetone	1278.6	M, L	C_10_H_12_O_2_	122-84-9	2.24	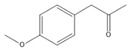
12	(E)-methyl isoeugenol	1401.8	M, L	C_11_H_14_O_2_	93-16-3	2.62	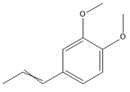
13	4-Hydroxy-3-methoxystyrene	1317.4	M, L	C_9_H_10_O_2_	7786-61-0	1.67	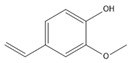
14	α-eudesmol	1651.7	M, L	C_15_H_26_O	473-16-5	0.77	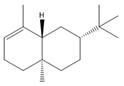
15	beta-Eudesmol	1650.1	M, L	C_15_H_26_O	473-15-4	2.76	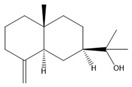
16	limonene glycol	1470.6	M, L	C_10_H_18_O_2_	1946-00-5	1.13	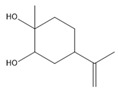
17	2,3-dihydrothiophene	596.6	M, L	C_4_H_6_S	1120-59-8	1.32	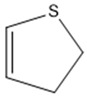
18	(3S)-3,4,4a,5,6,7-Hexahydro-4aβ,5β-dimethyl-3-isopropenylnaphthalen-1(2H)-one	1576.5	M, L	C_15_H_22_O	562-23-2	2.59	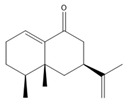
19	alpha-Asarone	1553.6	M, L	C_12_H_16_O_3_	2883-98-9	21.56	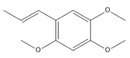
20	beta-Asarone	1521.4	M, L	C_12_H_16_O_3_	5273-86-9	3.7	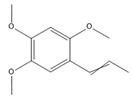
21	Senkyunolide	1356.4	M, L	C_12_H_16_O_2_	63038-10-8	4.88	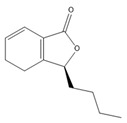
22	3-Ethyloctane	1272.1	M, L	C_10_H_22_	5881-17-4	3.78	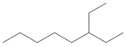
23	Palmitic acid	1968.4	M, L	C_16_H_32_O_2_	57-10-3	2.4	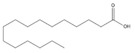

Note: M (contrast of the experimental MS with that available in libraries NIS). L (calculation of linear retention indexes).

## Data Availability

The datasets presented in the current study are available from the corresponding author on reasonable request.

## Supplementary Materials

The following supporting information can be downloaded at: https://www.mdpi.com/article/10.3390/biom14091102/s1, Table S1: CXEO component prediction with GC-MS; Table S2: FCEO component prediction with GC-MS. File S1: Western Blotting Figures.
