# Supplementary material for: Sedative-Hypnotic Effect and Mechanism of Carbon Nanofiber Loaded with Essential Oils of Ligusticum chuanxiong (Ligusticum chuanxiong Hort.) and Finger Citron (Citrus medica L. var. sarcodactylis) on Mice Models of Insomnia
Source: Biomolecules. 2024 Sep 2;14(9):1102. doi: 10.3390/biom14091102 (PMC11430208; doi:10.3390/biom14091102)
Supplement: Supplementary file 1 [file biomolecules-14-01102-s001.zip › biomolecules-3128217-supplementary.pdf]

GC-MS analysis of CXEO and FCEO

Table S1 . Results of chemical composition analysis of CXEO based on GC-MS

Number	Compounds	LRItab	Identification	Molecular formula	CAS	Relative (%)	Structural formula
1	(+)-Camphor	1143.4	M,L	$C_{10}H_{16}O$	464-49-3	2.74	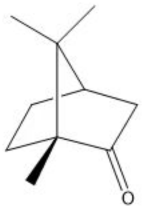
2	Linalool	1099.0	M,L	$C_{10}H_{18}O$	78-70-6	0.98	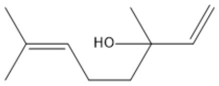
3	Terpinen-4-ol	1177.1	M,L	$C_{10}H_{18}O$	562-74-3	13.95	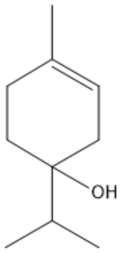
4	alpha-Longipinene	1352.1	M,L	$C_{15}H_{24}$	5989-08-2	2.01	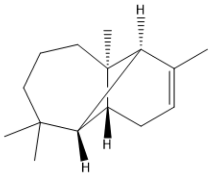
5	(-)-Alpha-Cubebene	1351.4	M,L	$C_{15}H_{24}$	17699-14-8	0.47	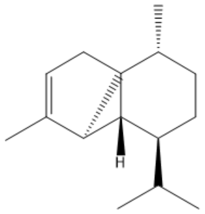
6	Naphthalene, 1,2,4a,5,8,8a-hexahydro-4,7-dimethyl-1-(1-methylethyl)-, (1S,4aR,8aS)-	1465.5	M,L	$C_{15}H_{24}$	523-47-7	1.11	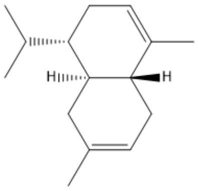

7	bornyl formate	1222.6	M,L	$C_{11}H_{18}O_2$	7492-41-3	2.81	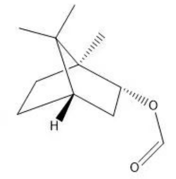
8	(+)-DELTA-CADINENE	1523.2	M,L	$C_{15}H_{24}$	483-76-1	3.52	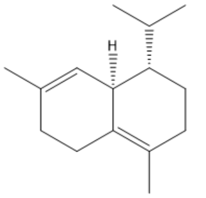
9	calamenene	1522.9	M,L	$C_{15}H_{22}$	483-77-2	0.2	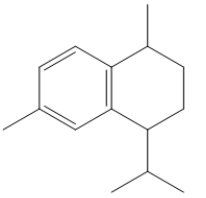
10	(R)-gamma-cadinene	1513.1	M,L	$C_{15}H_{24}$	39029-41-9	0.46	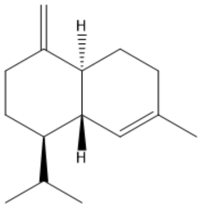
11	8-Methylenedispiro[2.1.2.4]undecane	1362.0	M,L	$C_{12}H_{18}$	51567-08-9	1.01	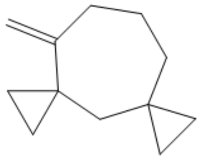
12	(-)-isoledene	1482.4	M,L	$C_{15}H_{24}$	95910-36-4	0.64	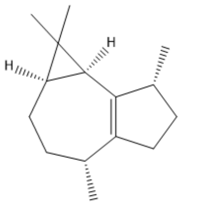
13	1,2,3,4,6,8alpha-Hexahydro-1-isopropyl-4,7-dimethylnaphthalene	1440.6	M,L	$C_{15}H_{24}$	16728-99-7	3.69	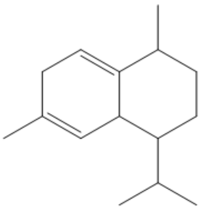

14	alpha-elemol	1547.5	M,L	$C_{15}H_{26}O$	639-99-6	15.3	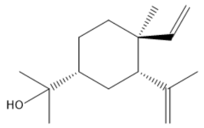
15	isocaryophyllene	1406.5	M,L	$C_{15}H_{24}$	118-65-0	1.21	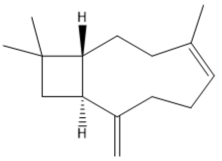
16	gamma-eudesmol	1630.9	M,L	$C_{15}H_{26}O$	1209-71-8	4.39	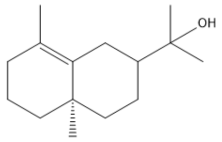
17	Naphthalene, 1,2,4a,5,8,8a-hexahydro-4,7-dimethyl-1-(1-methylethyl)-, (1R,4aS,8aR)-rel-	1434.5	M,L	$C_{15}H_{24}$	5951-61-1	0.78	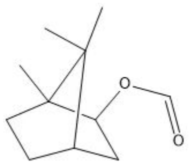
18	T-muurolol	1640.8	M,L	$C_{15}H_{26}O$	19912-62-0	1.82	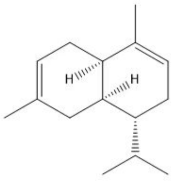
19	α -eudesmol	1651.7	M,L	$C_{15}H_{26}O$	473-16-5	4.55	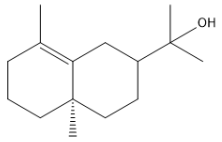
20	beta-Eudesmol	1650.1	M,L	$C_{15}H_{26}O$	473-15-4	4.88	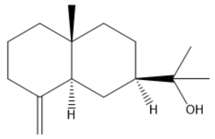
21	2,4-Di-t-butylphenol	1612.3	M,L	$C_{14}H_{22}O$	96-76-4	0.5	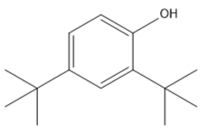

22	abieta-7,13-diene	2080.5	M,L	$C_{20}H_{32}$	35241-40-8	1.59	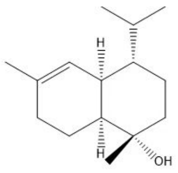
23	alpha-bulnesene	1414.5	M,L	$C_{15}H_{24}$	3691-11-0	9.75	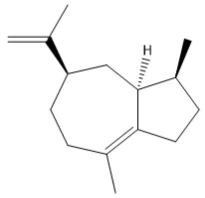
24	2-amino-6-butyl-5-methyl-1H-[1,2,4]triazolo[1,5-a]pyrimidin-7-one	1205.4	M,L	$C_{10}H_{15}N_5O$	873408-42-5	1.68	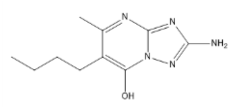
25	Z-Ligustilide	1523.7	M,L	$C_{12}H_{14}O_2$	81944-09-4	1.83	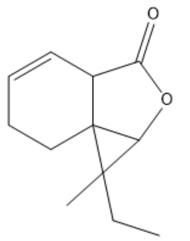

Note: M (contrast of the experimental MS with that available in libraries NIS). L (calculation of linear retention indexes)

Table S2 . Results of chemical composition analysis of FCEO based on GC-MS

Number	Compounds	LRItab	Identification	Molecular formula	CAS	Relative (%)	Structural formula
1	n-Ethyl propanoate	861.9	M,L	$C_5H_{10}O_2$	105-37-3	33.93	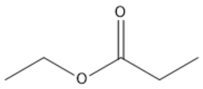
2	Ethyl isobutyrate	799.0	M,L	$C_6H_{12}O_2$	97-62-1	0.26	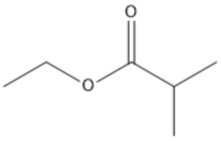
3	Propyl acetate	860.4	M,L	$C_5H_{10}O_2$	109-60-4	4.27	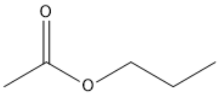
4	SEC-BUTYLACETATE	996.4	M,L	$C_6H_{12}O_2$	105-46-4	9.71	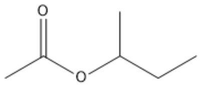
5	(±)-2-Butanol	660.2	M,L	$C_4H_{10}O$	78-92-2	0.81	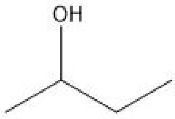
6	butyl acetate	785.3	M,L	$C_6H_{12}O_2$	123-86-4	24.61	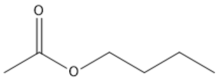
7	ether	814.9	M,L	C_8H_{10}	100-41-4	0.41	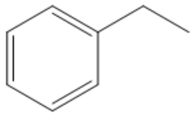
8	m-Xylene	823.8	M,L	C_8H_{10}	108-38-3	0.51	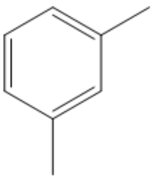
9	p-Xylene	848.1	M,L	C_8H_{10}	106-42-3	1.14	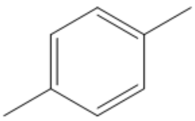

10	Butanol	660.2	M,L	$C_4H_{10}O$	71-36-3	0.58	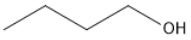
11	o-xylene	848.1	M,L	C_8H_{10}	95-47-6	0.61	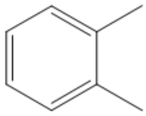
12	(+)-Limonene	1029.5	M,L	$C_{10}H_{16}$	5989-27-5	6.88	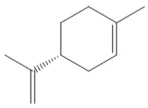
13	γ -terpinene	1059.7	M,L	$C_{10}H_{16}$	99-85-4	2.5	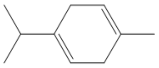
14	o-Cymene	1041.0	M,L	$C_{10}H_{14}$	527-84-4	2.27	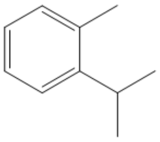
15	1,2,4,5-Tetramethylbenzene	1024.3	M,L	$C_{10}H_{14}$	95-93-2	0.26	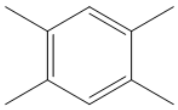
16	acetic acid	622.3	M,L	$C_2H_4O_2$	64-19-7	1.01	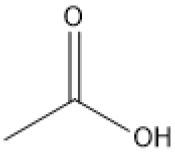
17	N-Methylpyrrolidone	737.5	M,L	C_5H_9NO	872-50-4	0.38	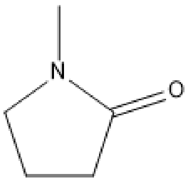
18	Naphthalene	1186.2	M,L	$C_{10}H_8$	91-20-3	0.78	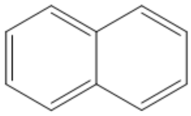
19	Aniline	913.2	M,L	C_6H_7N	62-53-3	0.28	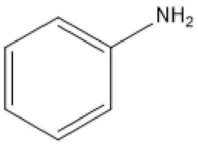

20	3,4-Dimethylbenzaldehyde	1253.9	M,L	$C_9H_{10}O$	5973-71-7	2.4	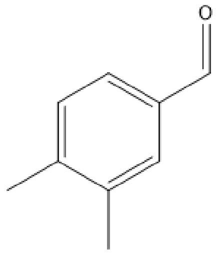
21	1-Methylnaphthalene	1394.6	M,L	$C_{11}H_{10}$	90-12-0	0.77	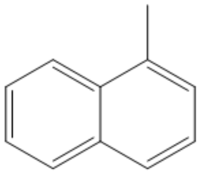
22	2-Methylnaphthalene	1243.0	M,L	$C_{11}H_{10}$	91-57-6	0.42	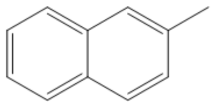
23	2,4-Di-t-butylphenol	1612.3	M,L	$C_{14}H_{22}O$	96-76-4	0.38	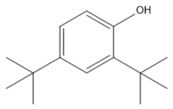
24	1,2-Benzenedicarboxylic acid	1951.1	M,L	$C_{16}H_{22}O_4$	84-74-2	1.15	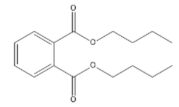
25	Tridecylic acid	1668.9	M,L	$C_{13}H_{26}O_2$	638-53-9	0.84	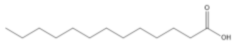

Note: M (contrast of the experimental MS with that available in libraries NIS). L (calculation of linear retention indexes)
