# Supplementary figures and images for: Sedative-Hypnotic Effect and Mechanism of Carbon Nanofiber Loaded with Essential Oils of Ligusticum chuanxiong (Ligusticum chuanxiong Hort.) and Finger Citron (Citrus medica L. var. sarcodactylis) on Mice Models of Insomnia
Source: Biomolecules. 2024 Sep 2;14(9):1102. doi: 10.3390/biom14091102 (PMC11430208; doi:10.3390/biom14091102)

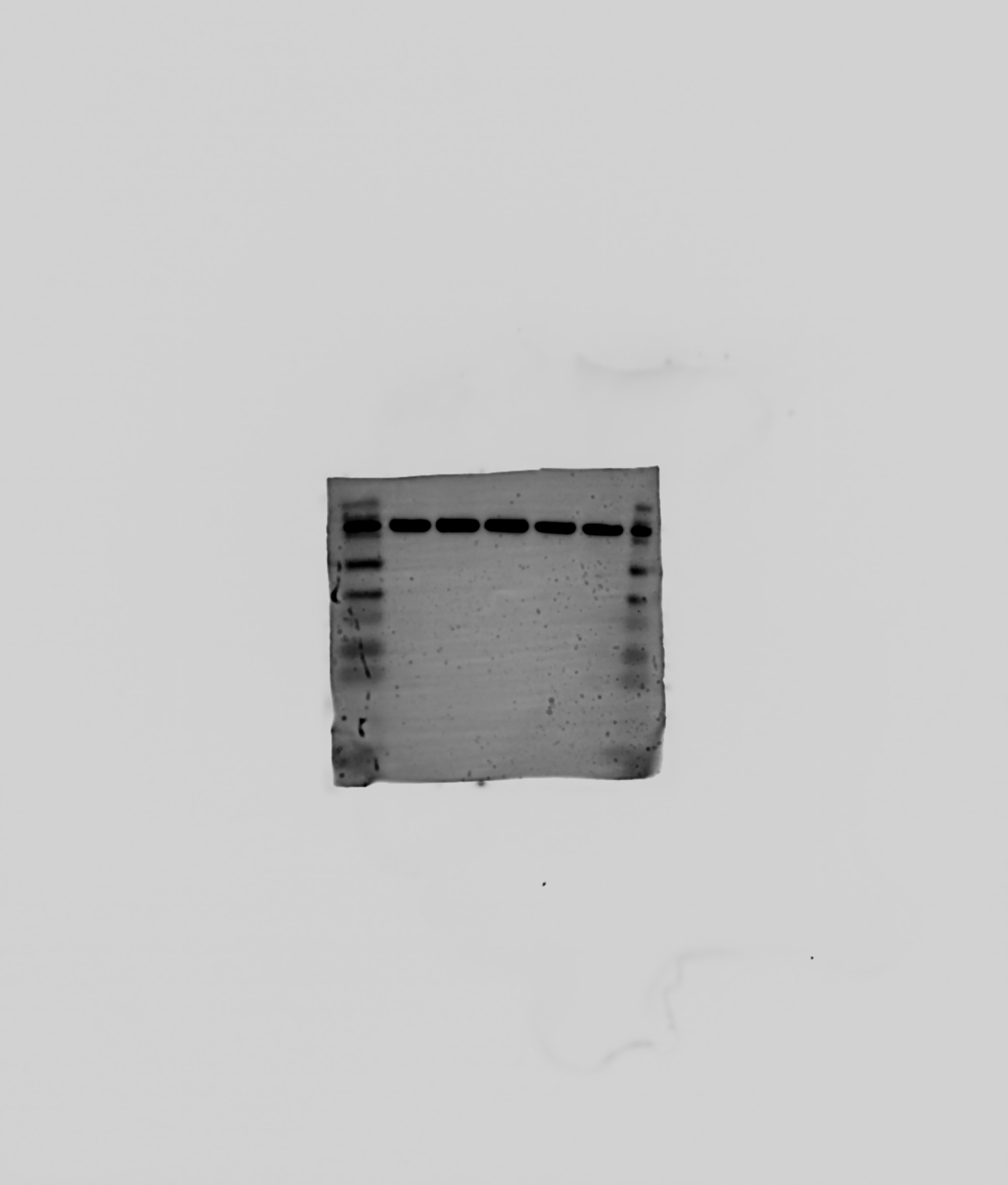

Supplement: Supplementary file 1 [file biomolecules-14-01102-s001.zip › Western Blot original images/JAK2/JAK2-1/JAK2.tif]

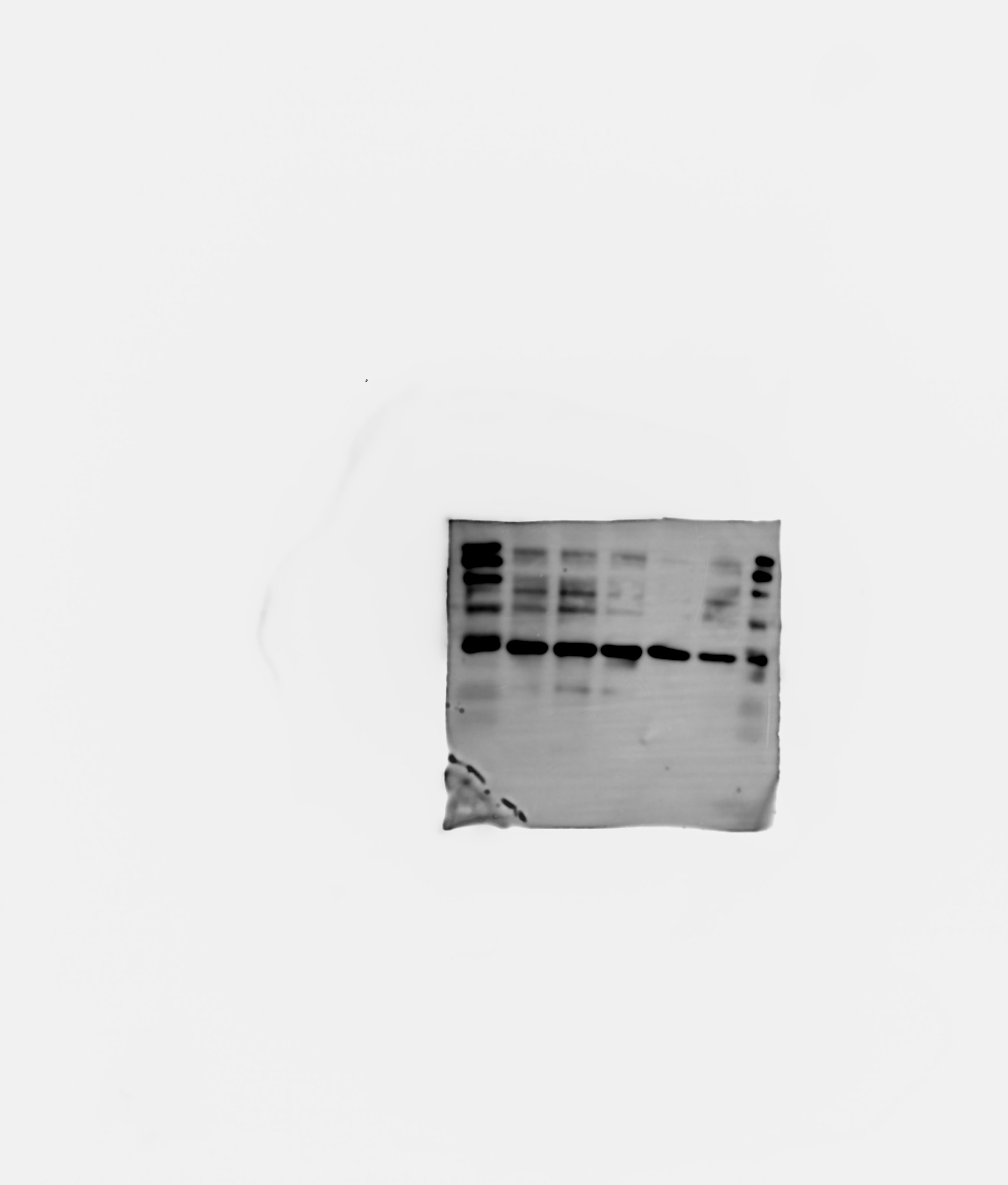

Supplement: Supplementary file 1 [file biomolecules-14-01102-s001.zip › Western Blot original images/JAK2/JAK2-1/β-actin.tif]

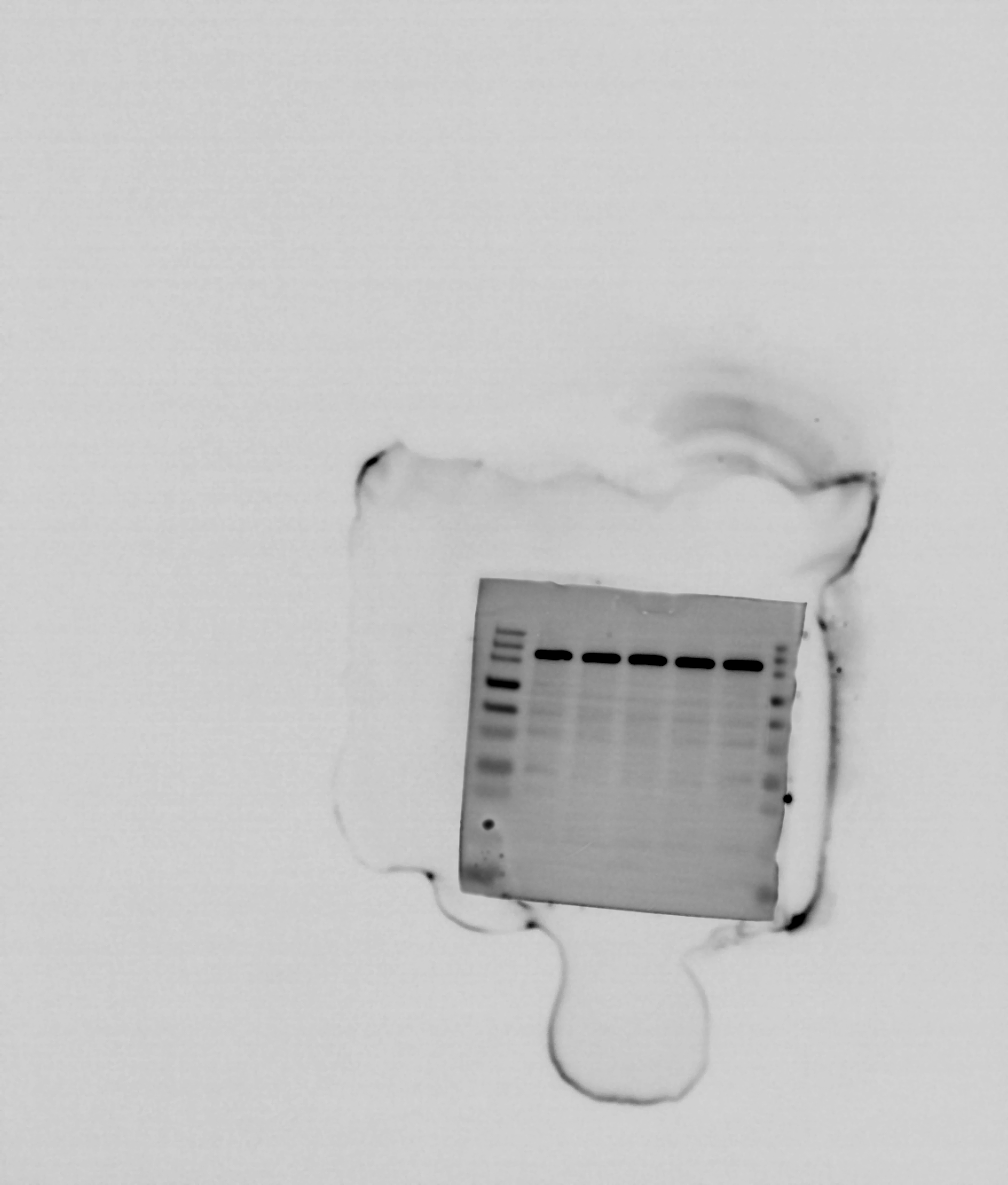

Supplement: Supplementary file 1 [file biomolecules-14-01102-s001.zip › Western Blot original images/JAK2/JAK2-2/JAK2.tif]

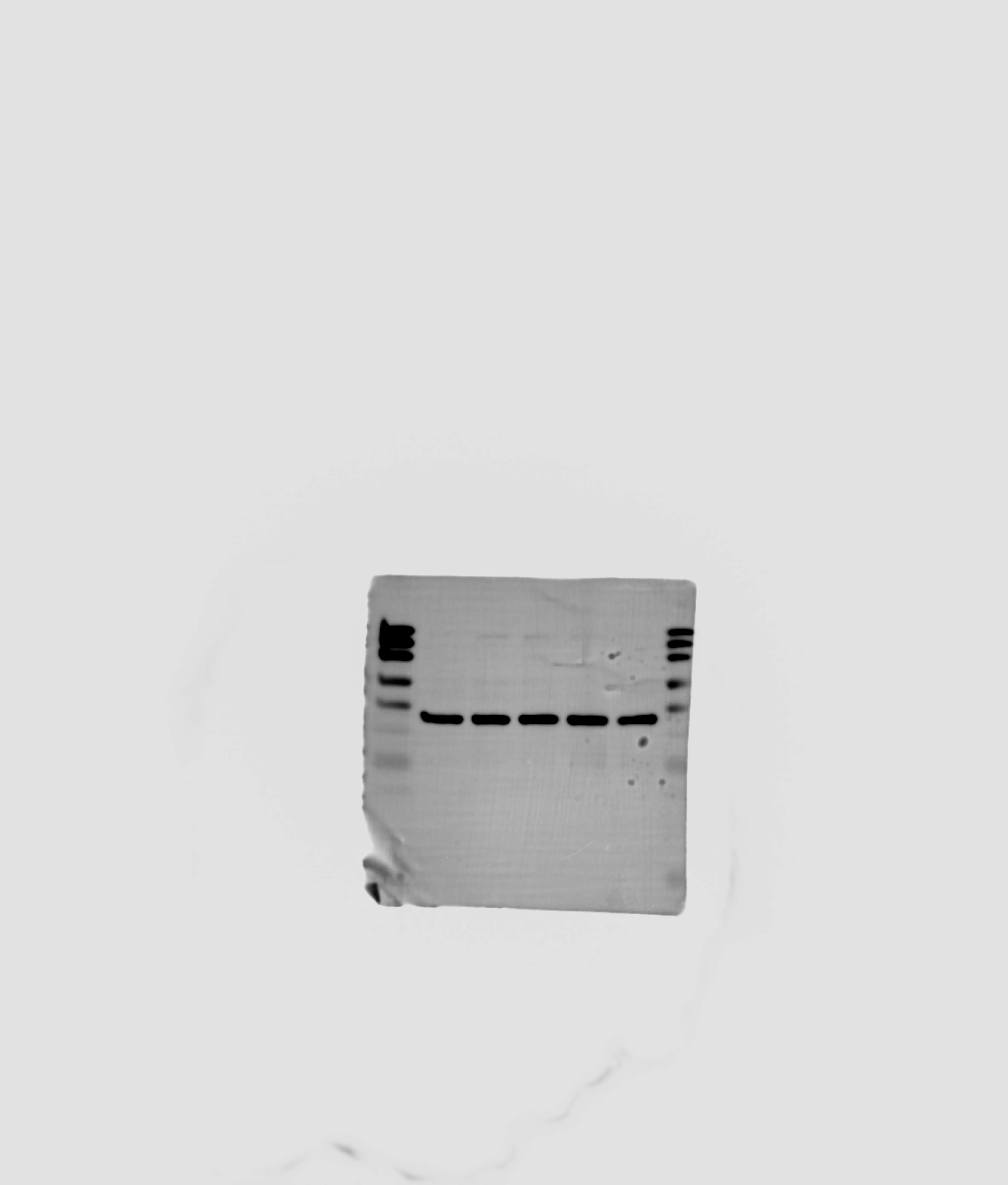

Supplement: Supplementary file 1 [file biomolecules-14-01102-s001.zip › Western Blot original images/JAK2/JAK2-2/β-actin.tif]

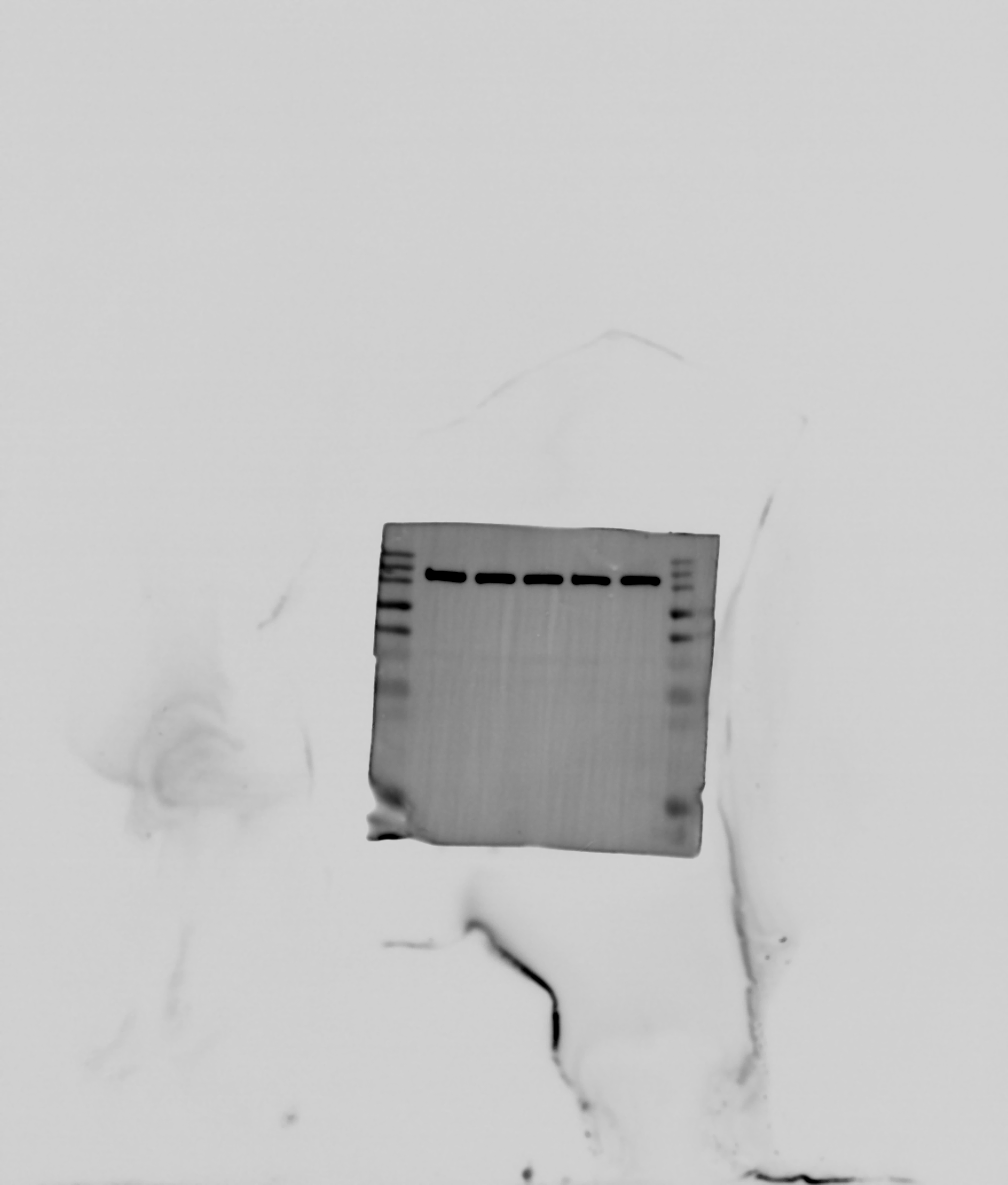

Supplement: Supplementary file 1 [file biomolecules-14-01102-s001.zip › Western Blot original images/JAK2/JAK2-3/JAK2.tif]

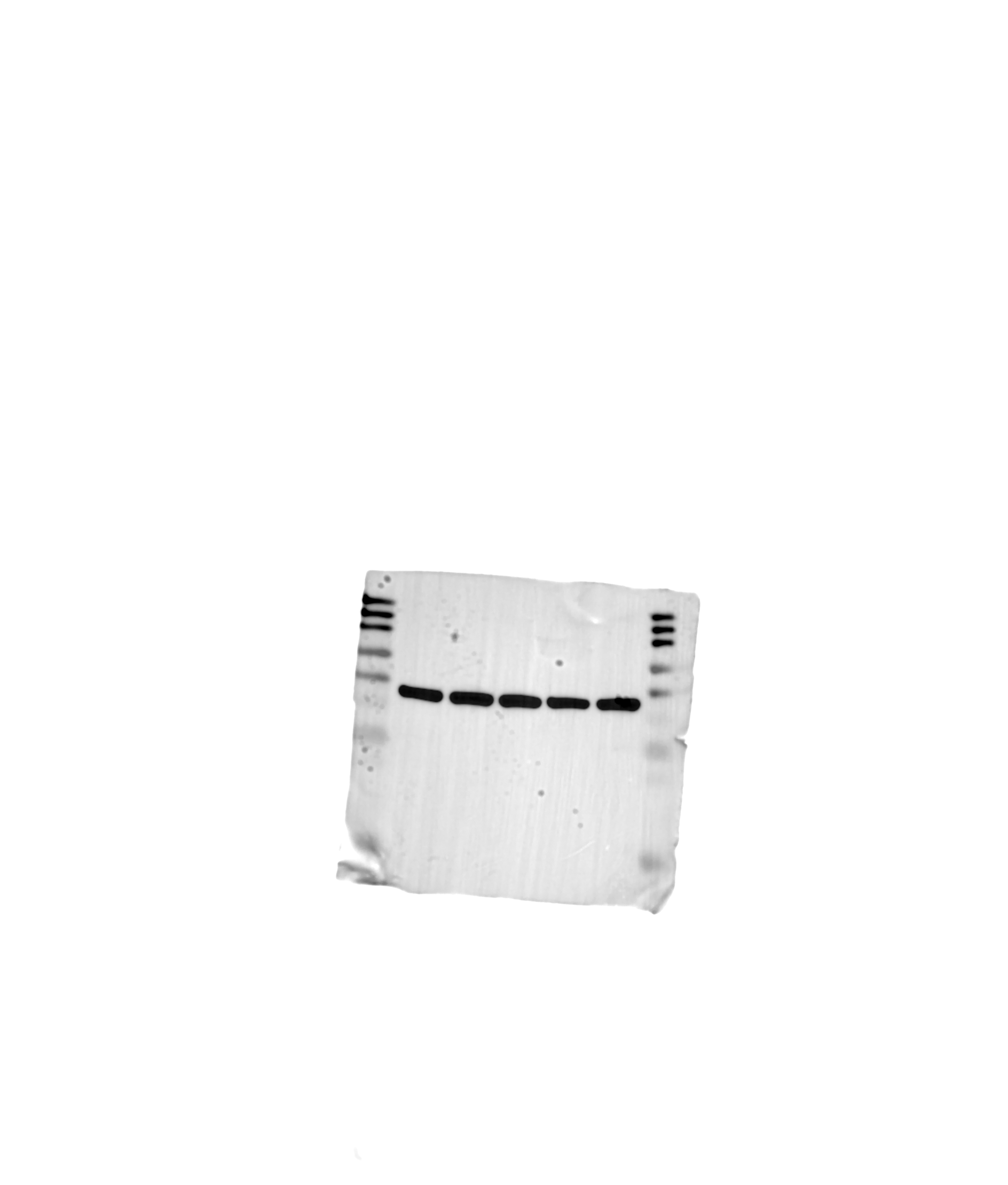

Supplement: Supplementary file 1 [file biomolecules-14-01102-s001.zip › Western Blot original images/JAK2/JAK2-3/β-actin.tif]

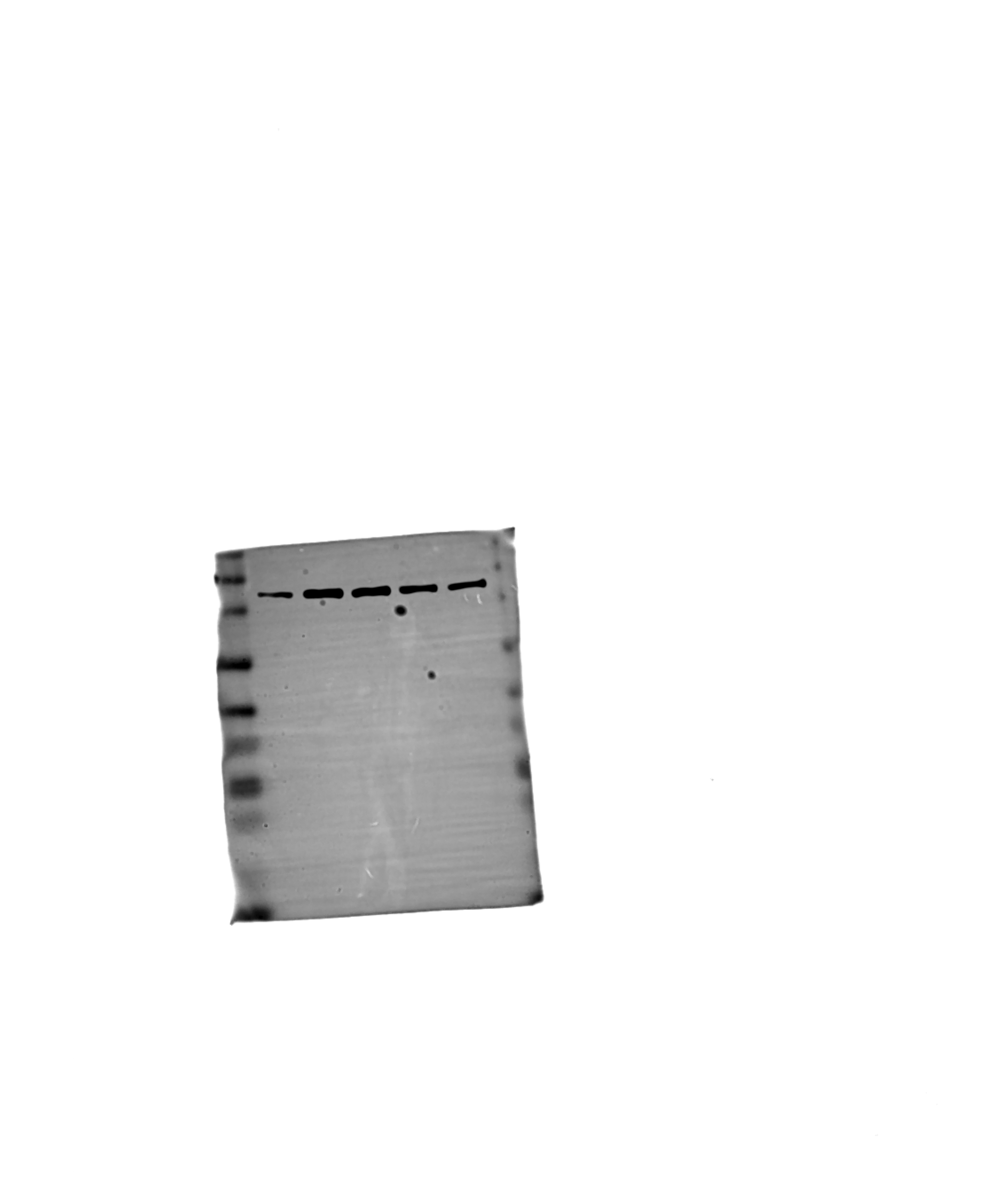

Supplement: Supplementary file 1 [file biomolecules-14-01102-s001.zip › Western Blot original images/P-JAK2/P-JAK2-1/P-JAK2.tif]

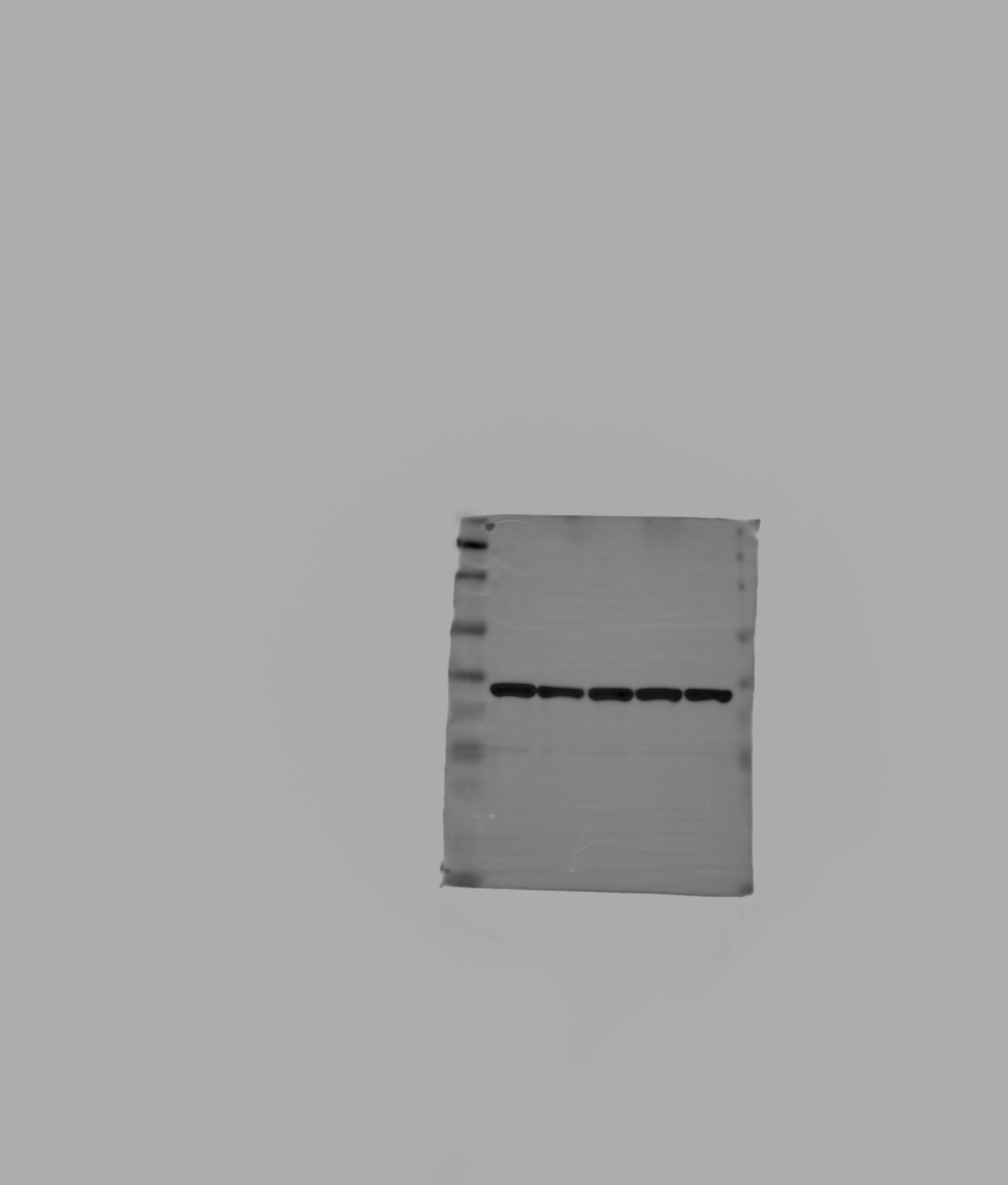

Supplement: Supplementary file 1 [file biomolecules-14-01102-s001.zip › Western Blot original images/P-JAK2/P-JAK2-1/β-actin.tif]

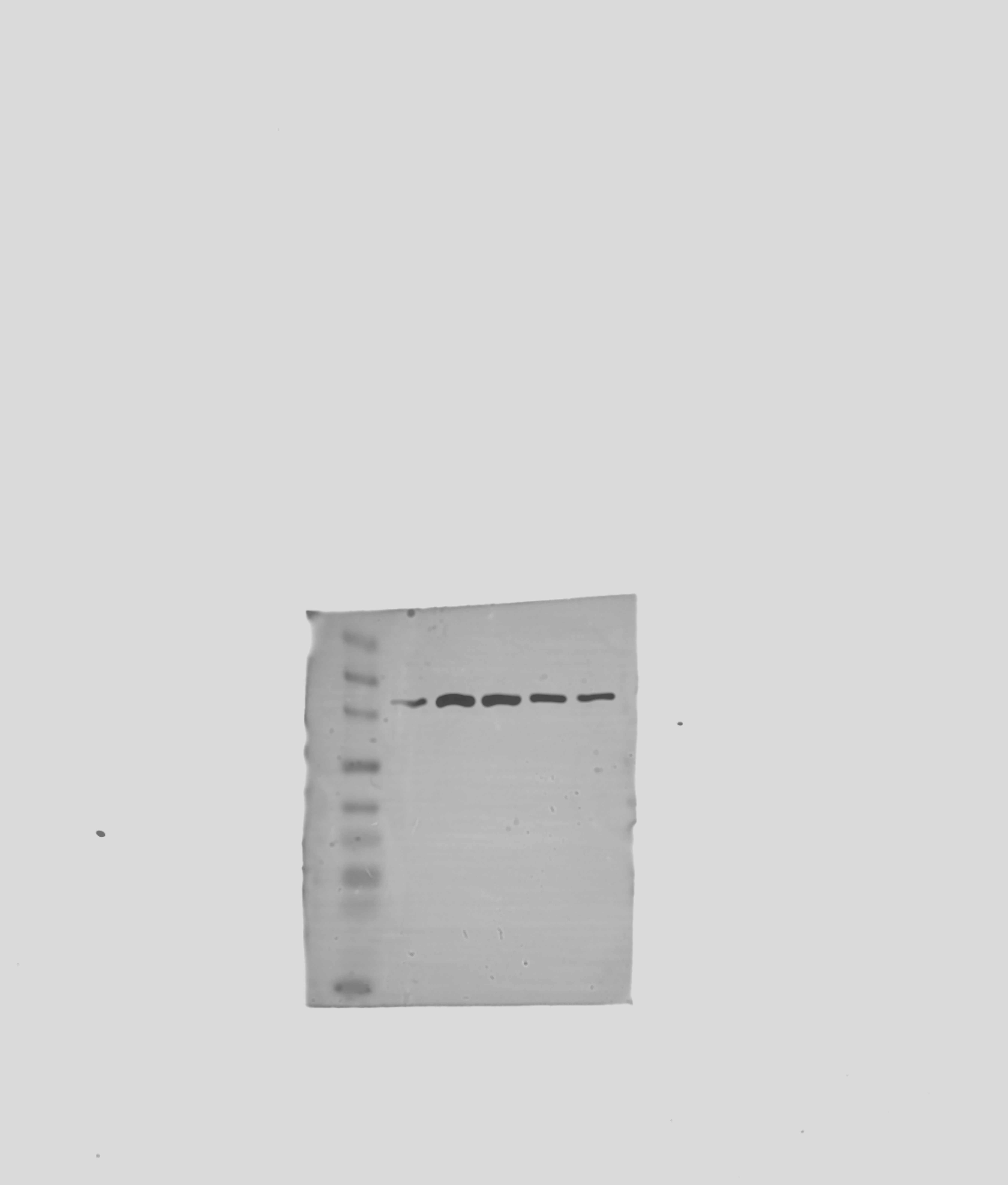

Supplement: Supplementary file 1 [file biomolecules-14-01102-s001.zip › Western Blot original images/P-JAK2/P-JAK2-2/P-JAK2.tif]

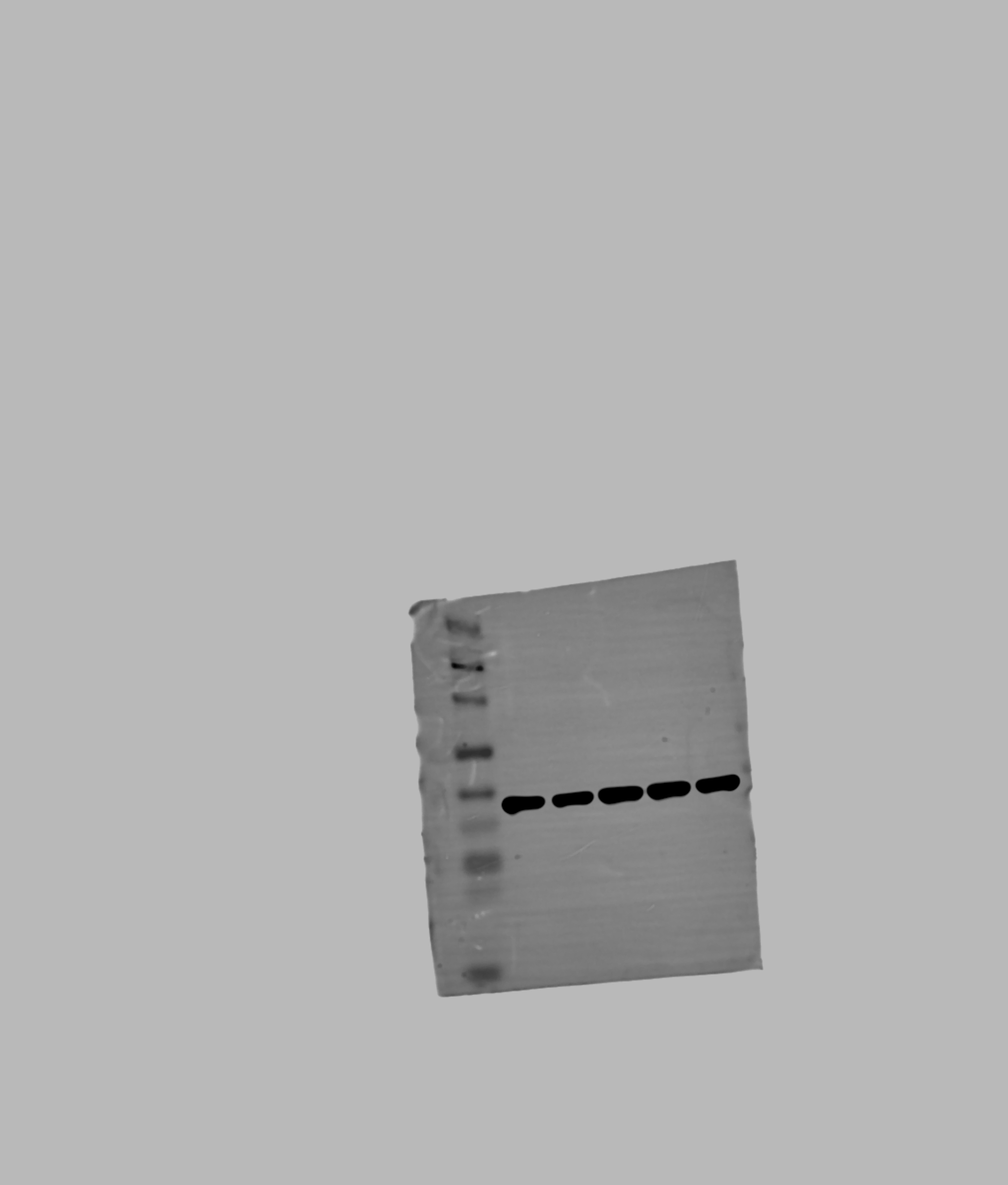

Supplement: Supplementary file 1 [file biomolecules-14-01102-s001.zip › Western Blot original images/P-JAK2/P-JAK2-2/β-actin.tif]

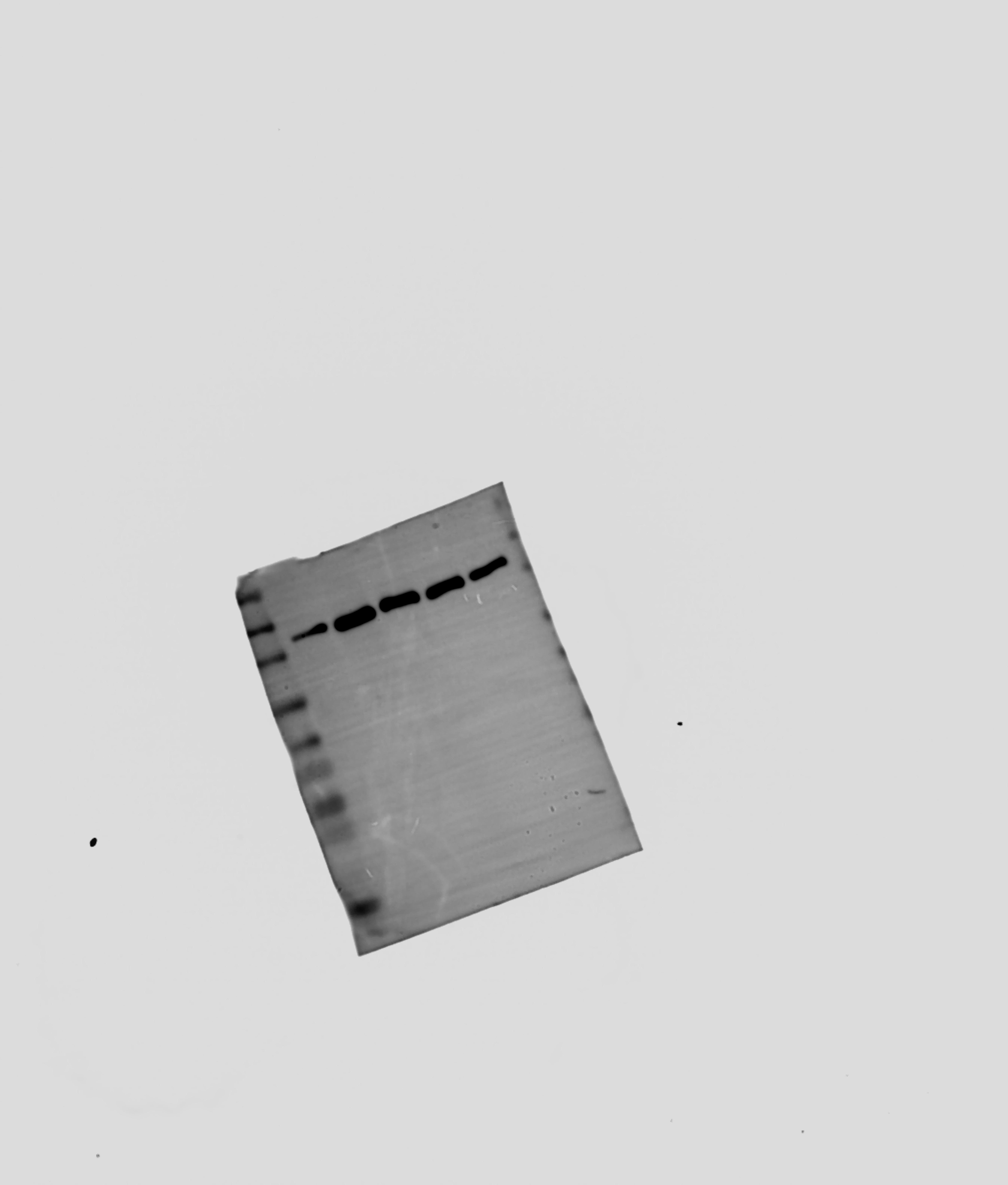

Supplement: Supplementary file 1 [file biomolecules-14-01102-s001.zip › Western Blot original images/P-JAK2/P-JAK2-3/P-JAK2.tif]

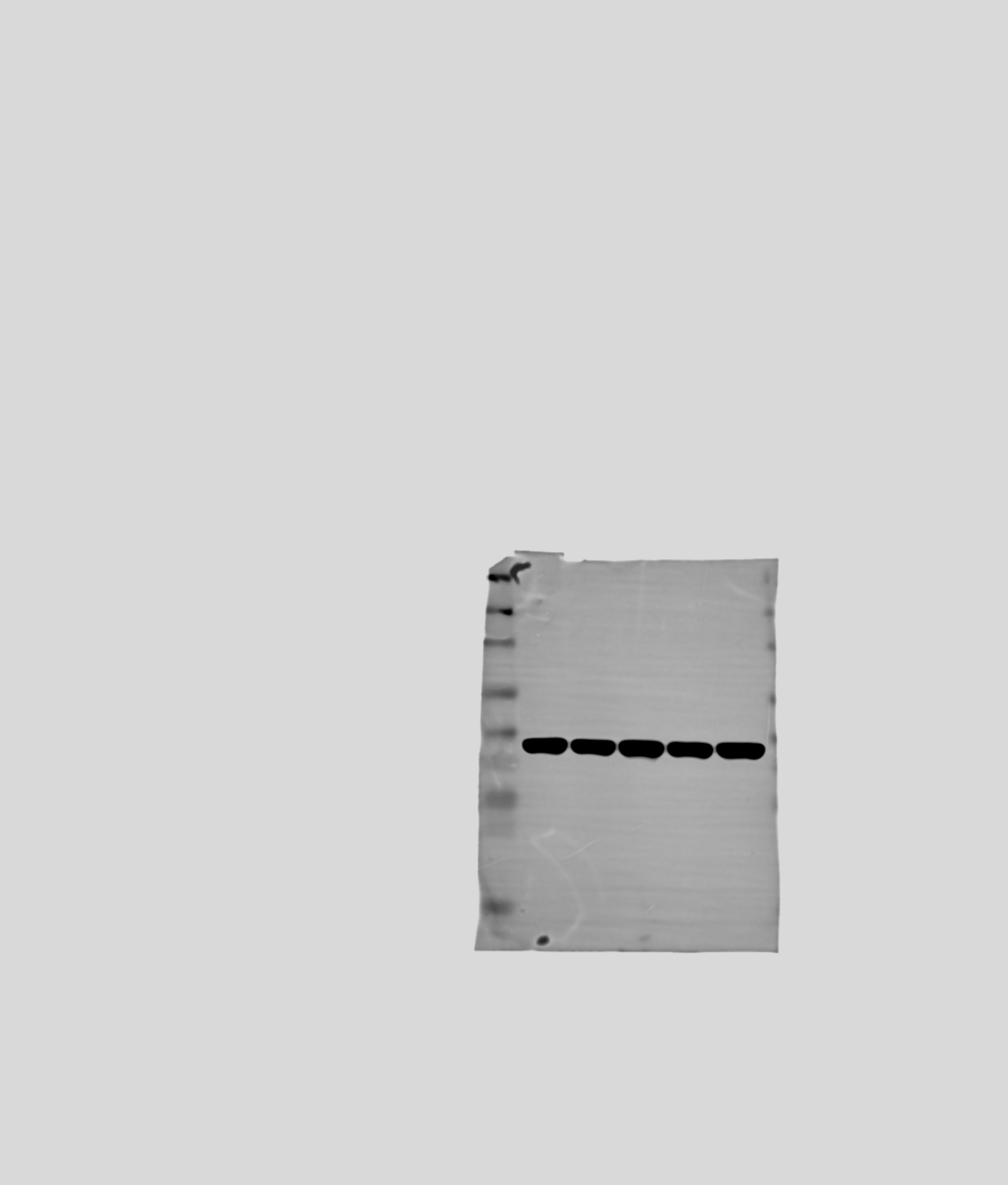

Supplement: Supplementary file 1 [file biomolecules-14-01102-s001.zip › Western Blot original images/P-JAK2/P-JAK2-3/β-actin.tif]

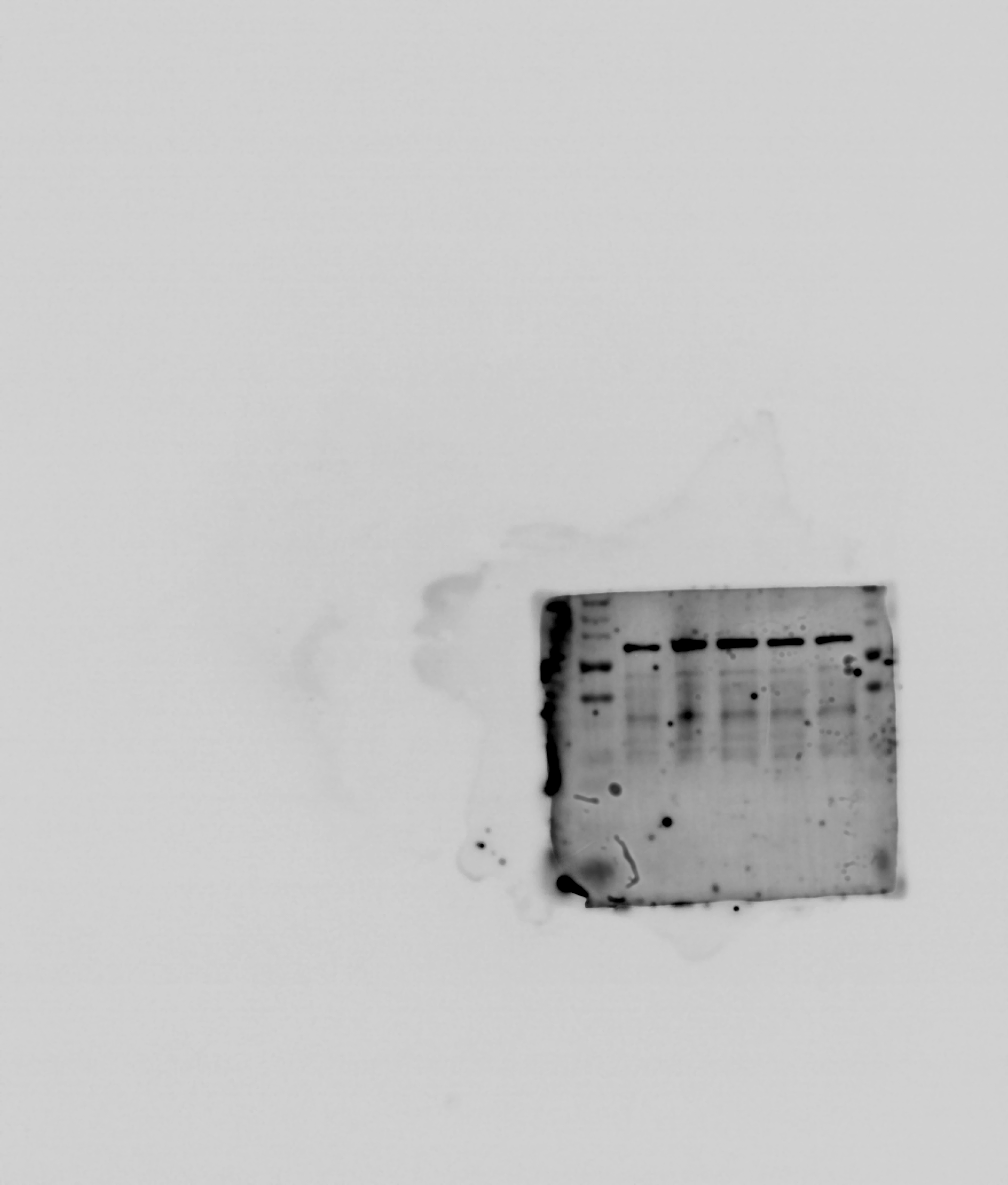

Supplement: Supplementary file 1 [file biomolecules-14-01102-s001.zip › Western Blot original images/P-STAT3/P-STAT3-1/P-STAT3.tif]

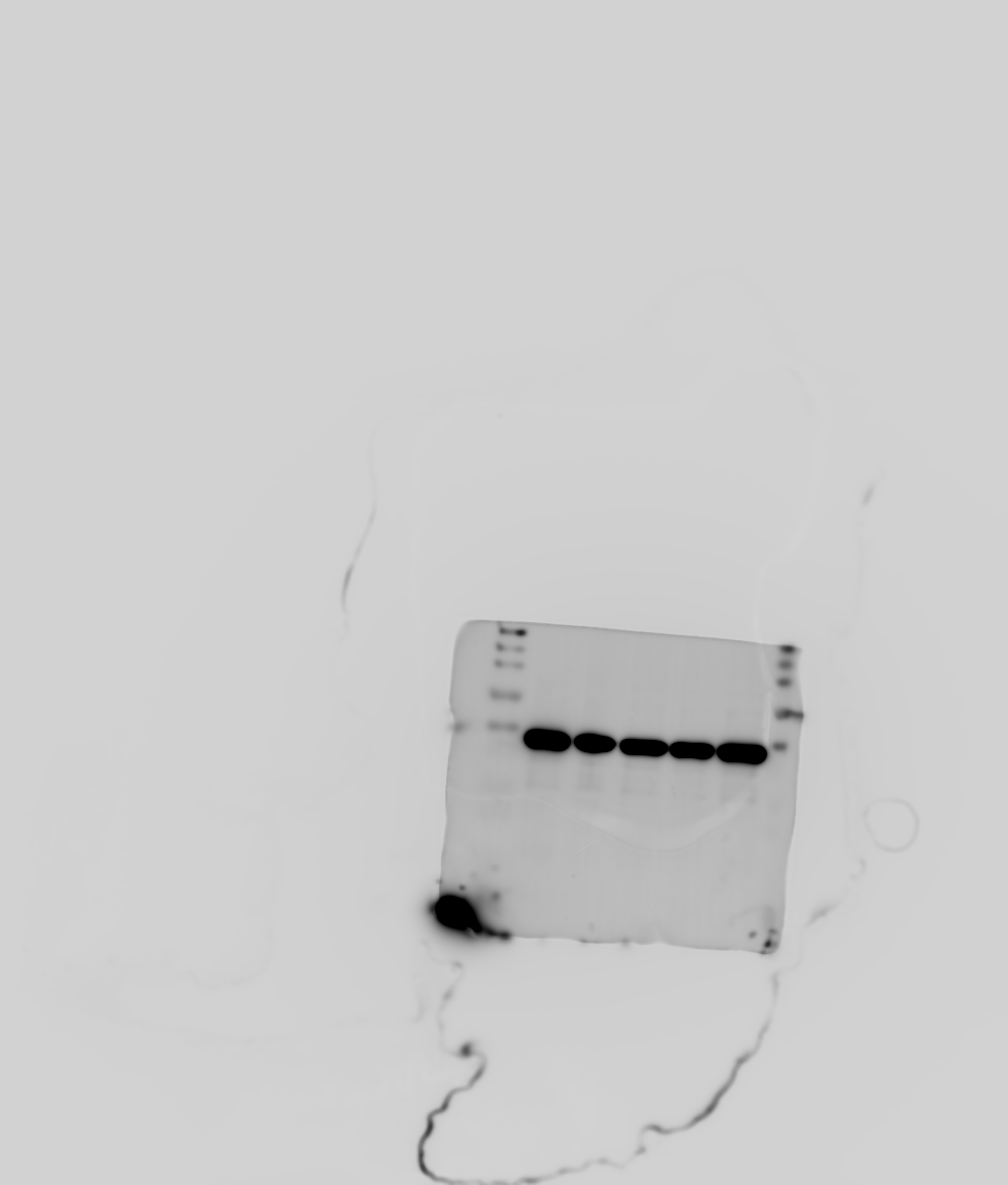

Supplement: Supplementary file 1 [file biomolecules-14-01102-s001.zip › Western Blot original images/P-STAT3/P-STAT3-1/β-actin.tif]

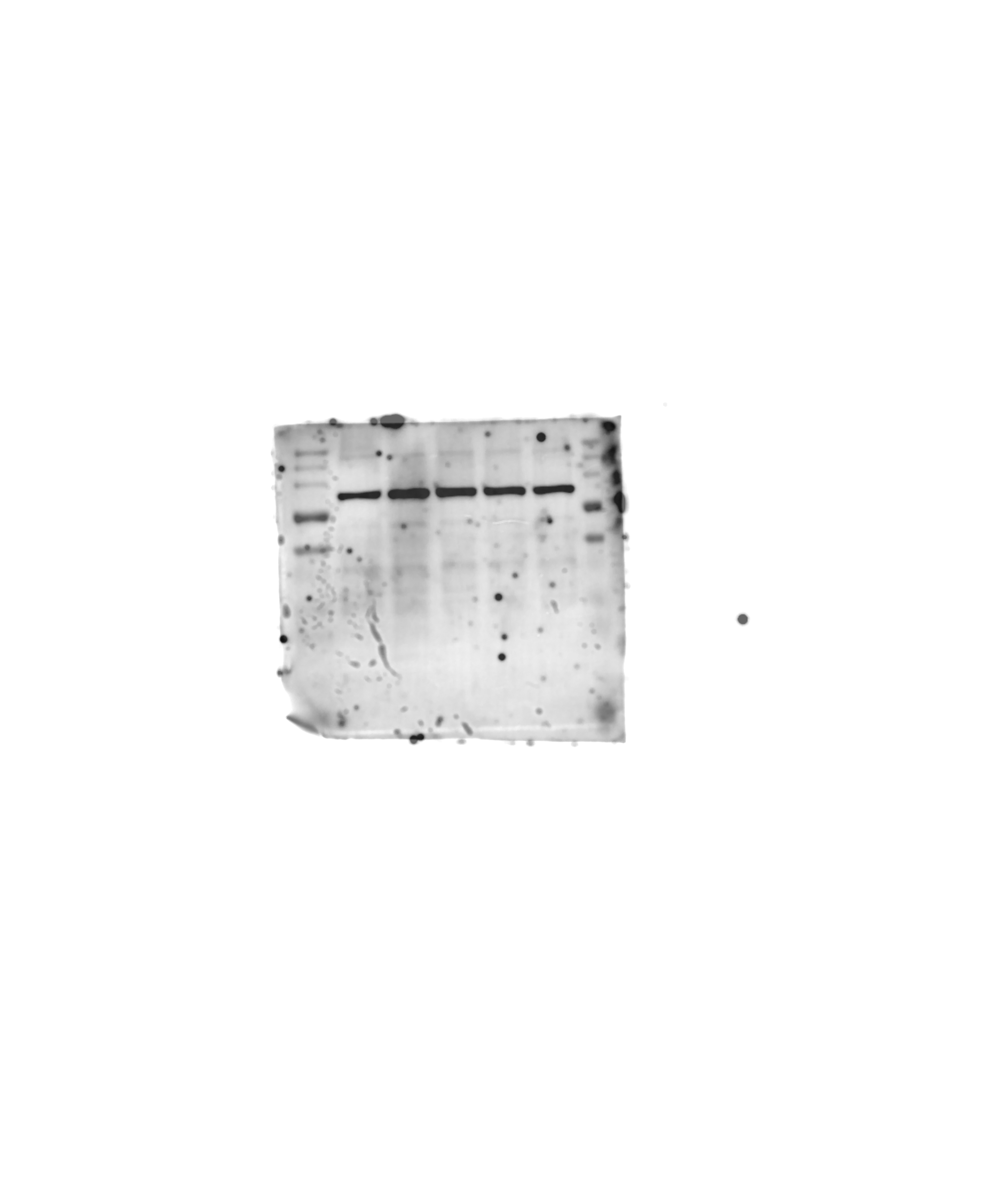

Supplement: Supplementary file 1 [file biomolecules-14-01102-s001.zip › Western Blot original images/P-STAT3/P-STAT3-2/P-STAT3.tif]

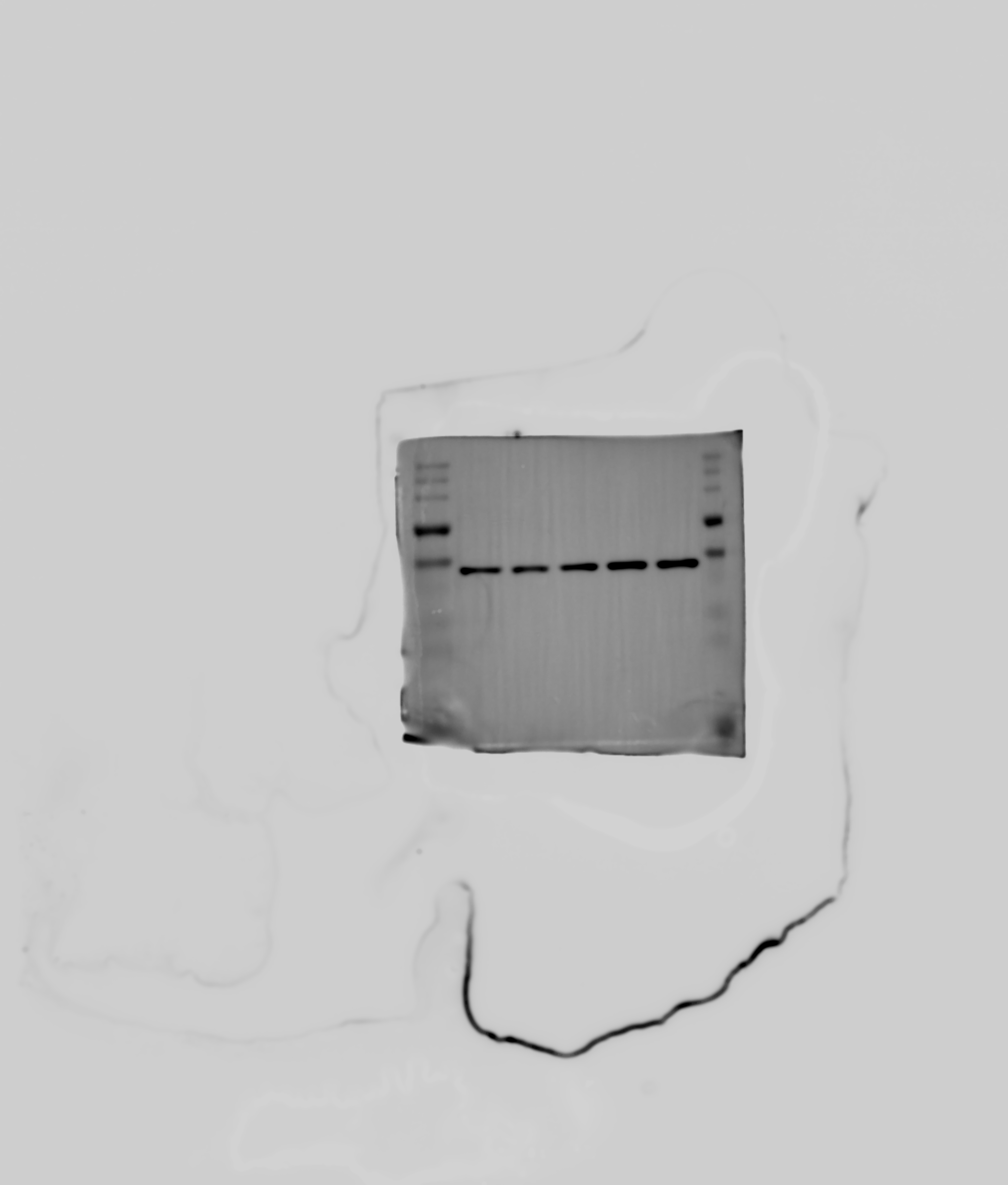

Supplement: Supplementary file 1 [file biomolecules-14-01102-s001.zip › Western Blot original images/P-STAT3/P-STAT3-2/β-actin.tif]

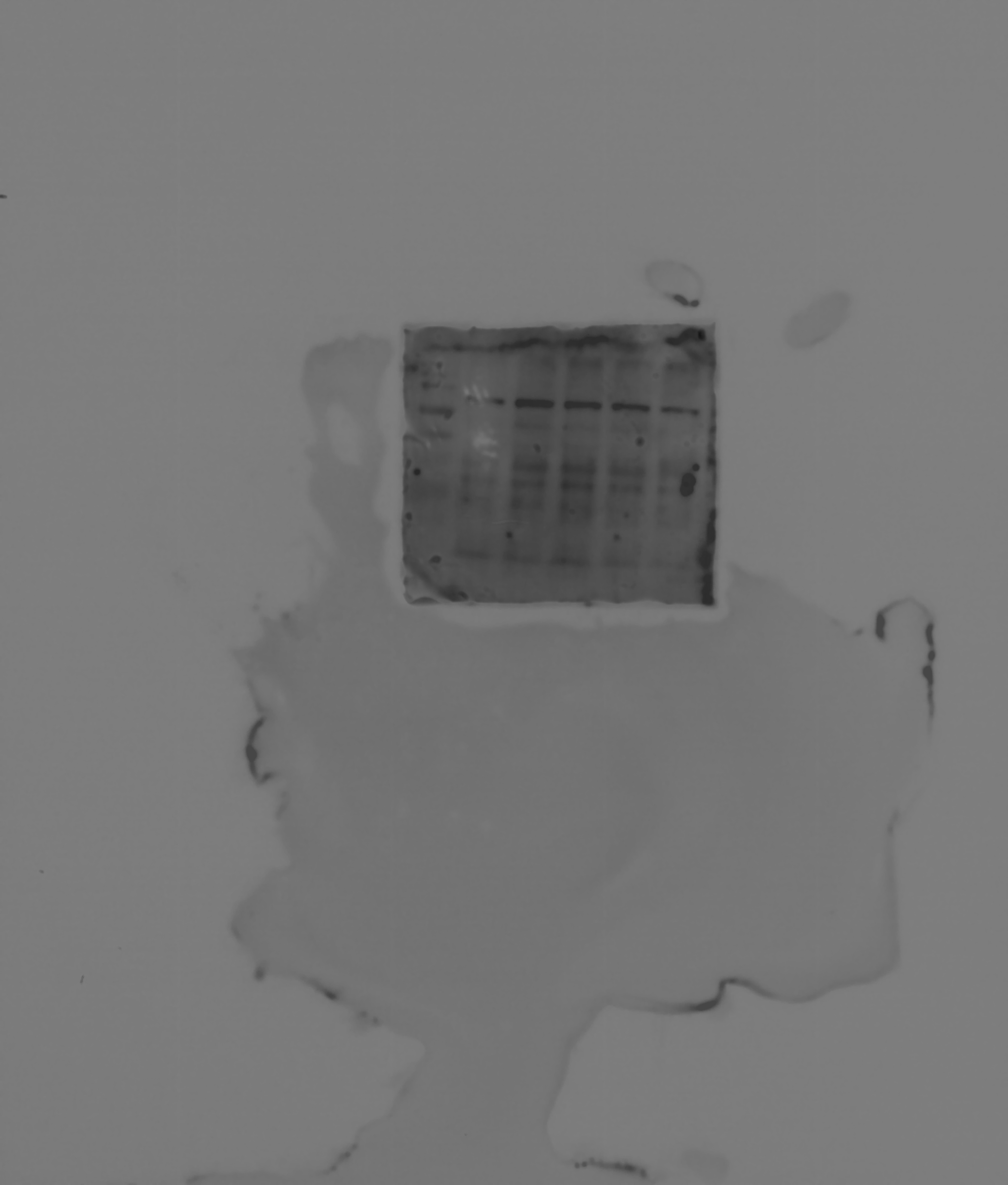

Supplement: Supplementary file 1 [file biomolecules-14-01102-s001.zip › Western Blot original images/P-STAT3/P-STAT3-3/P-STAT3.tif]

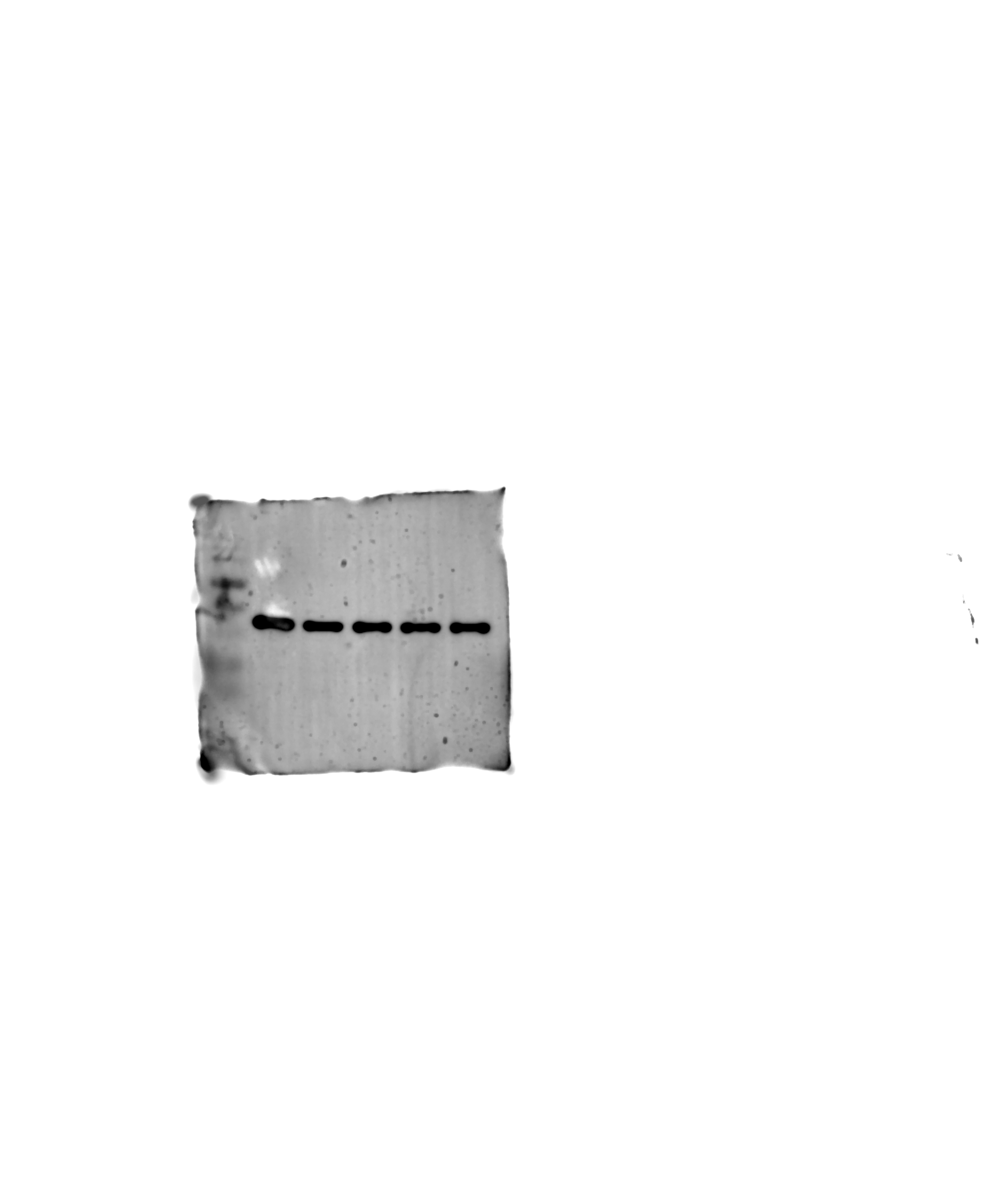

Supplement: Supplementary file 1 [file biomolecules-14-01102-s001.zip › Western Blot original images/P-STAT3/P-STAT3-3/β-actin.tif]

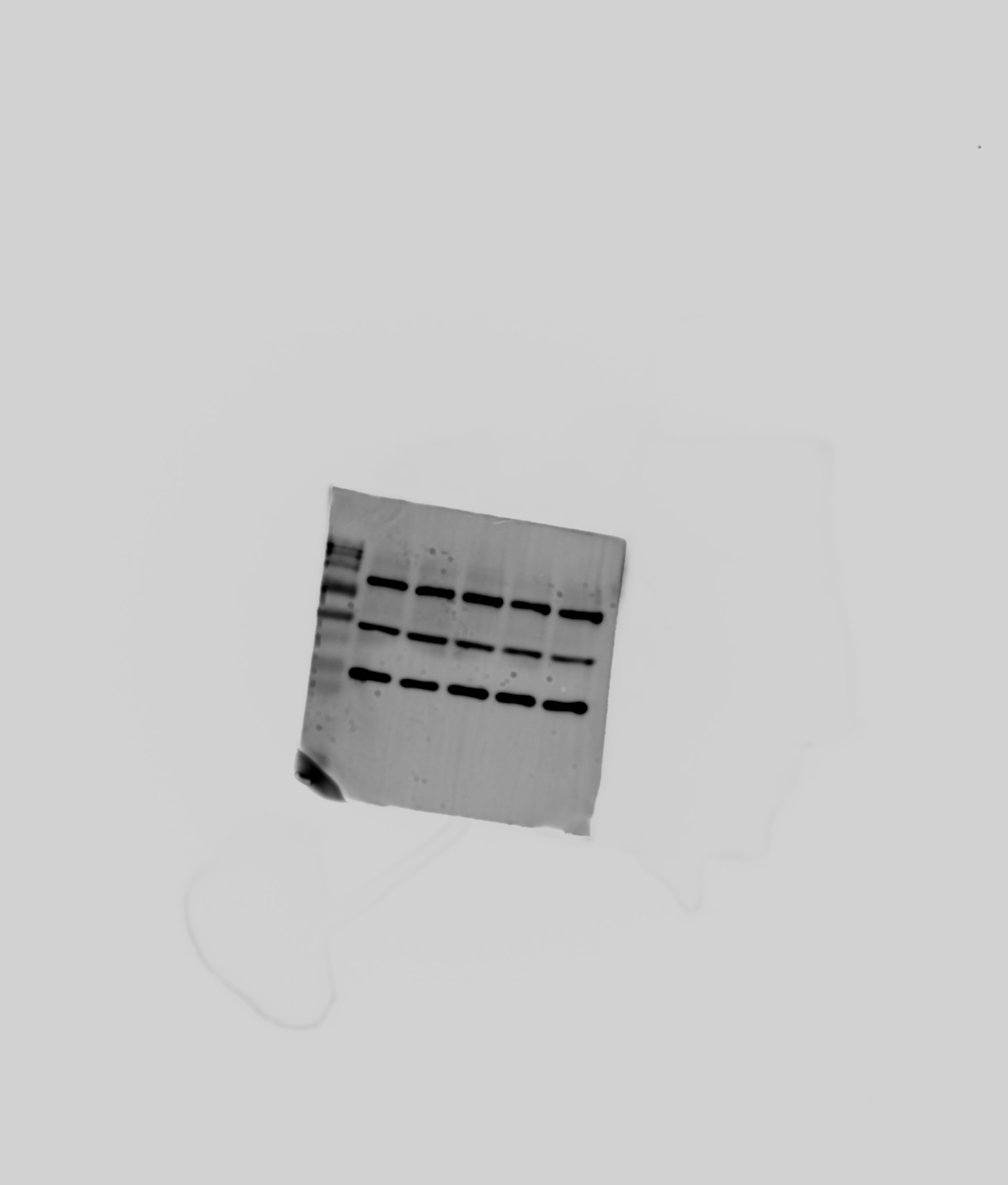

Supplement: Supplementary file 1 [file biomolecules-14-01102-s001.zip › Western Blot original images/SOCS3/SOCS3-1/SOCS3.tif]

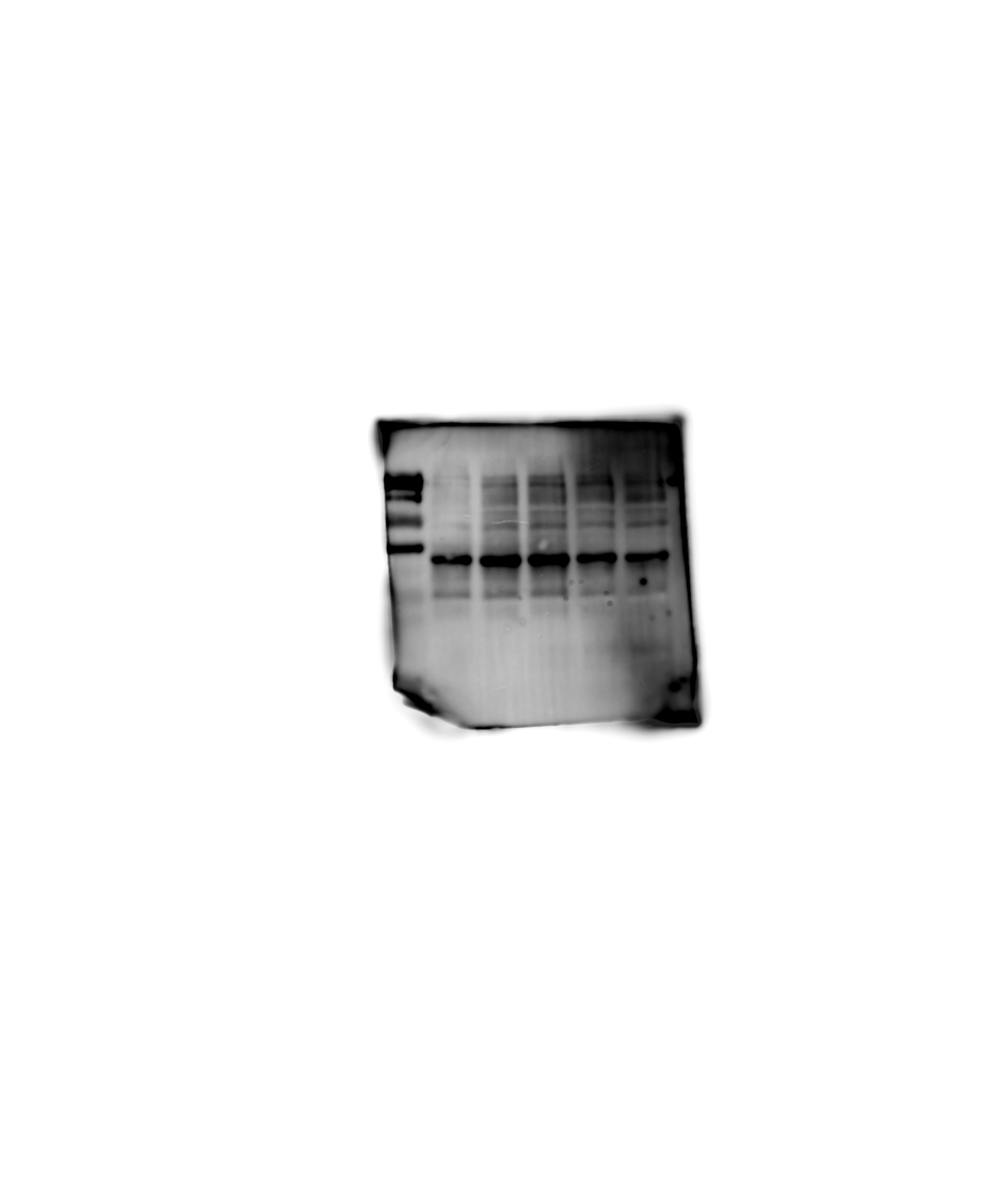

Supplement: Supplementary file 1 [file biomolecules-14-01102-s001.zip › Western Blot original images/SOCS3/SOCS3-1/β-actin.tif]

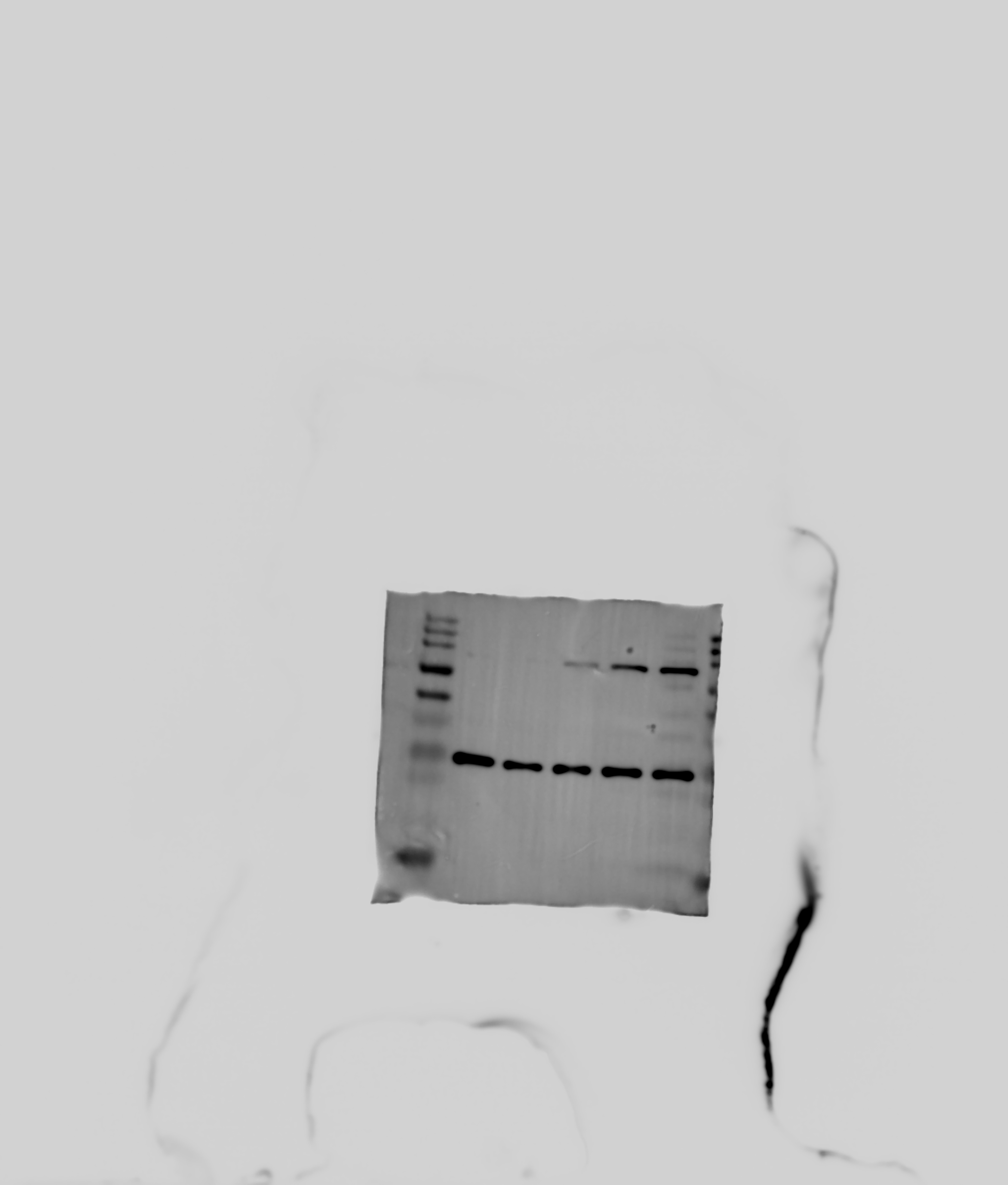

Supplement: Supplementary file 1 [file biomolecules-14-01102-s001.zip › Western Blot original images/SOCS3/SOCS3-2/SOCS3.tif]

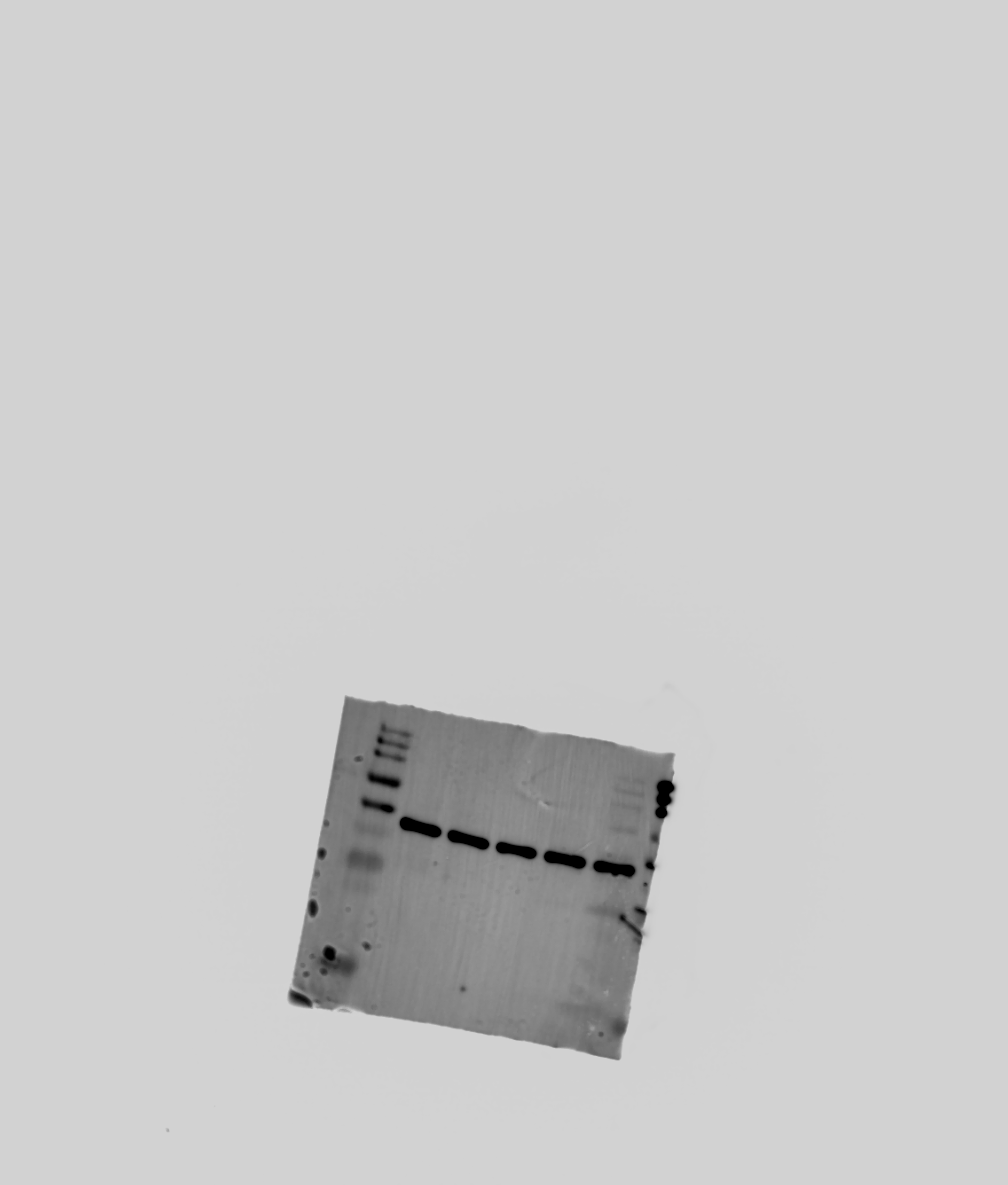

Supplement: Supplementary file 1 [file biomolecules-14-01102-s001.zip › Western Blot original images/SOCS3/SOCS3-2/β-actin.tif]

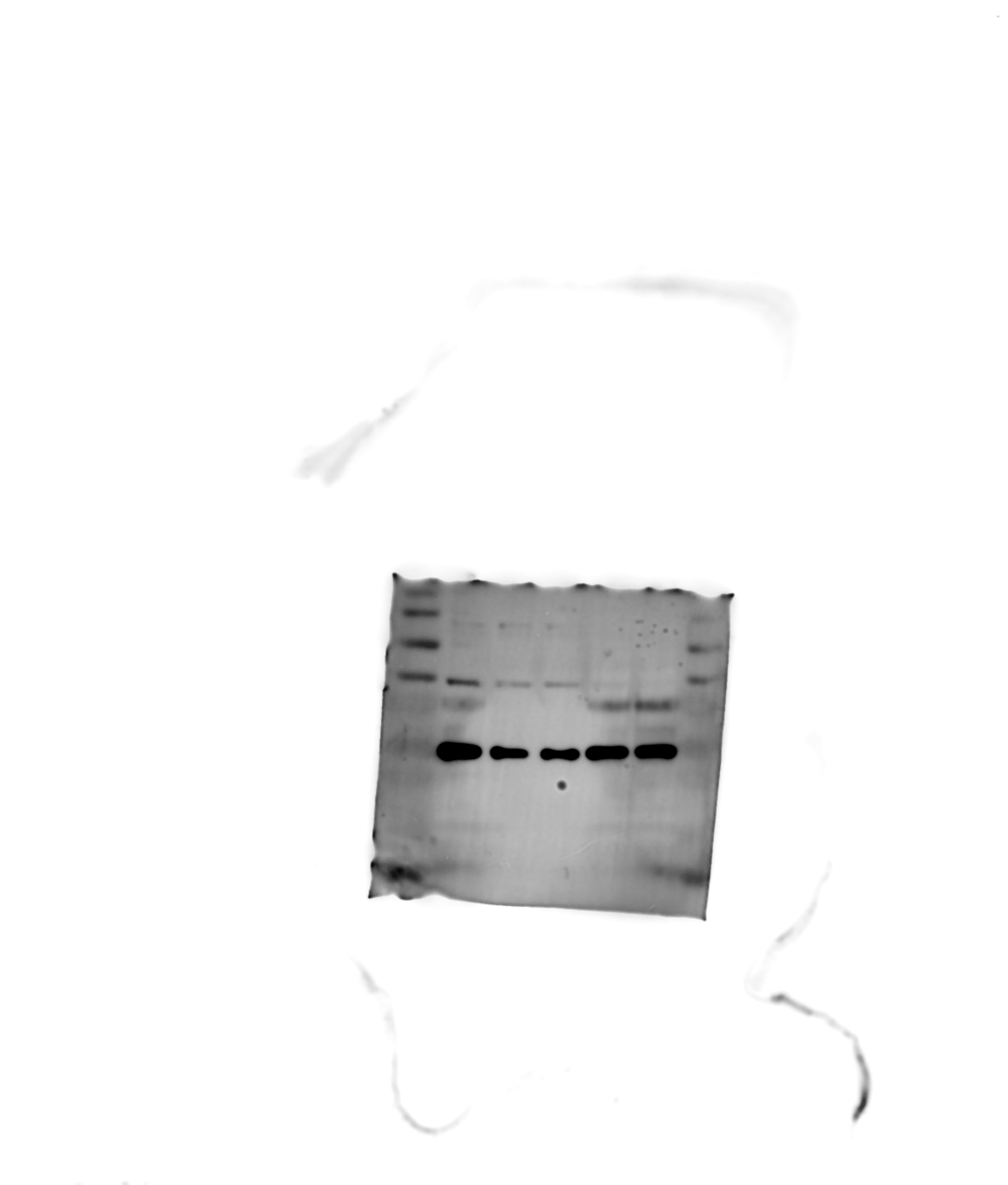

Supplement: Supplementary file 1 [file biomolecules-14-01102-s001.zip › Western Blot original images/SOCS3/SOCS3-3/SOCS3.tif]

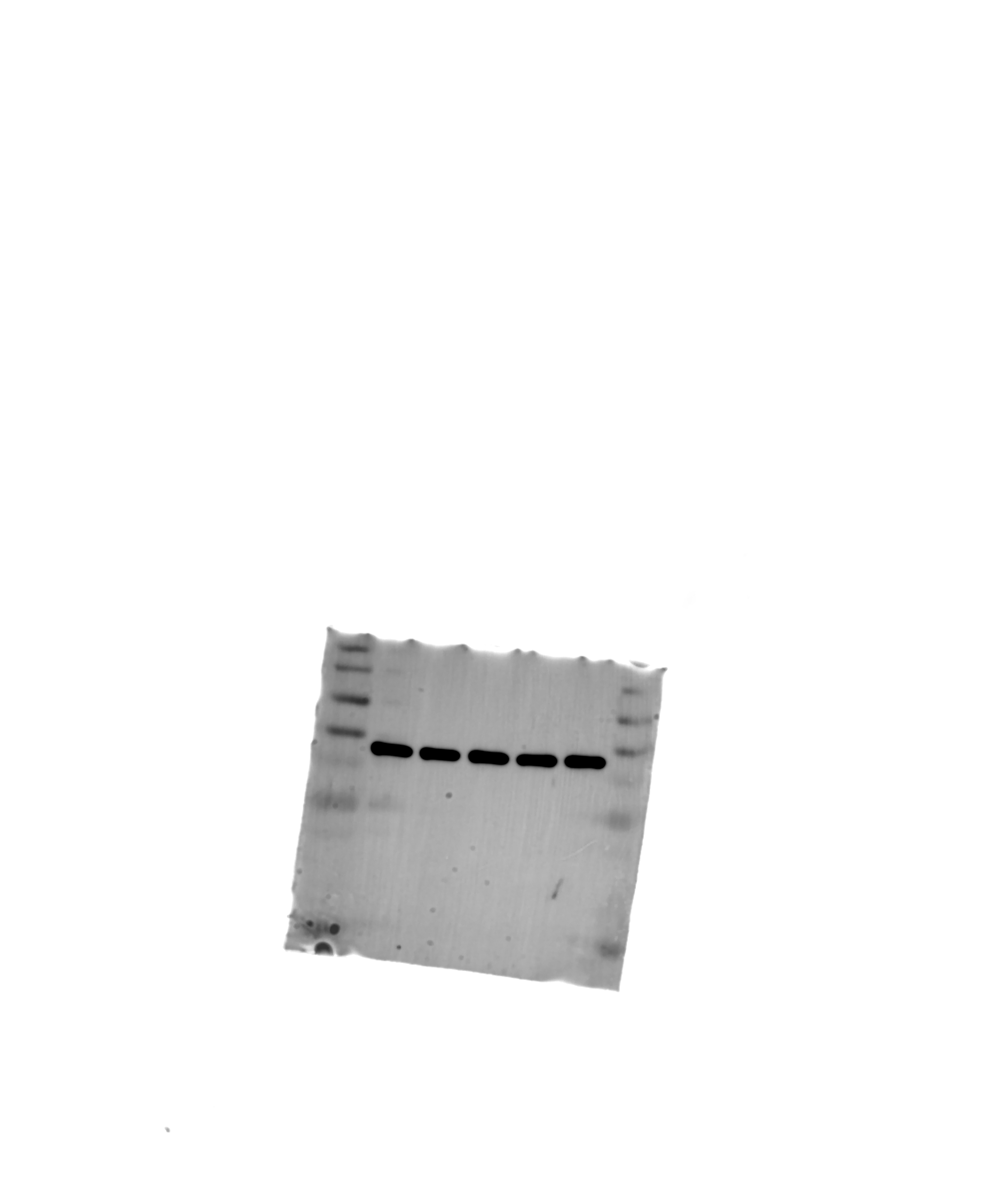

Supplement: Supplementary file 1 [file biomolecules-14-01102-s001.zip › Western Blot original images/SOCS3/SOCS3-3/β-actin.tif]

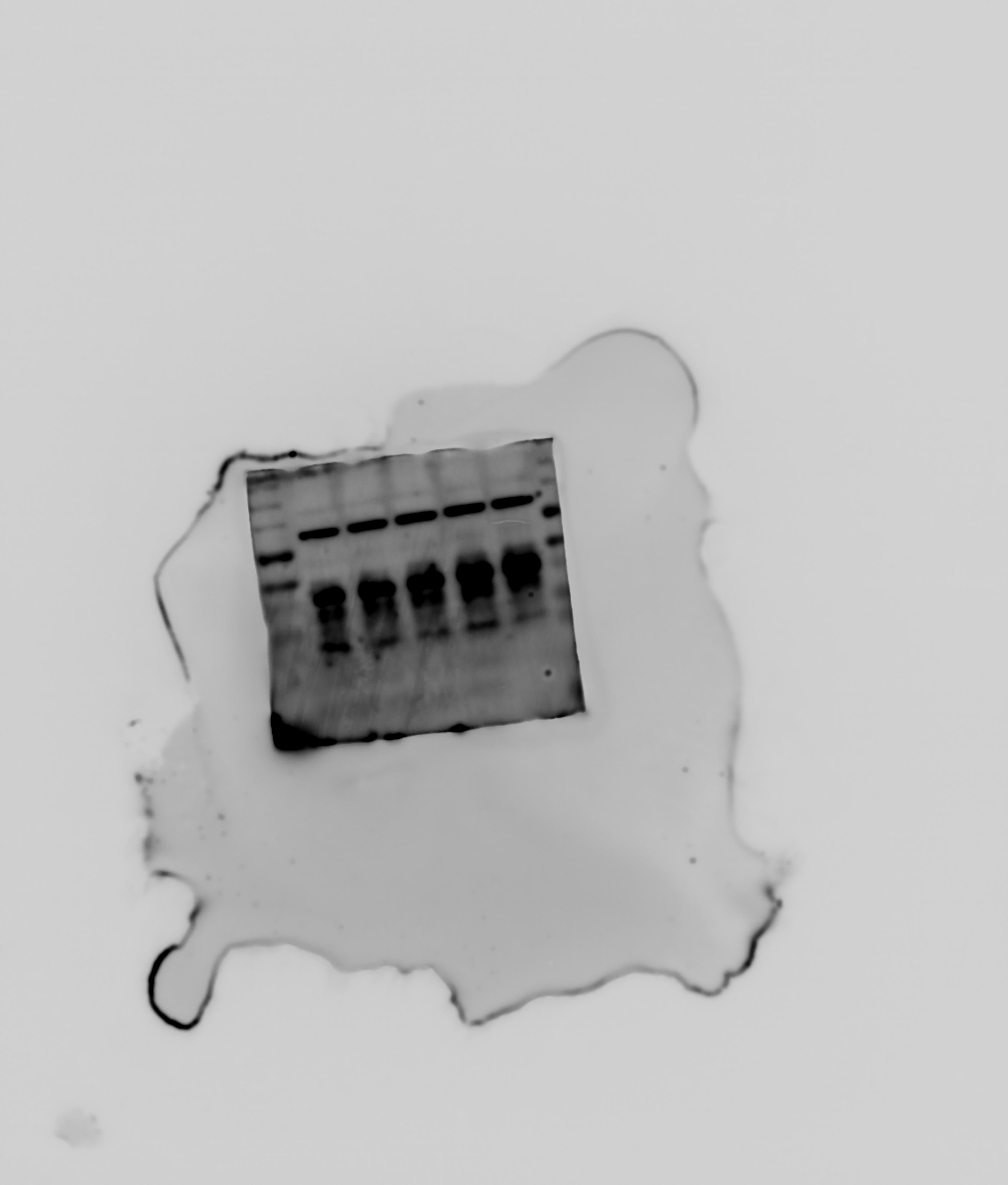

Supplement: Supplementary file 1 [file biomolecules-14-01102-s001.zip › Western Blot original images/STAT3/STAT3-1/STAT3.tif]

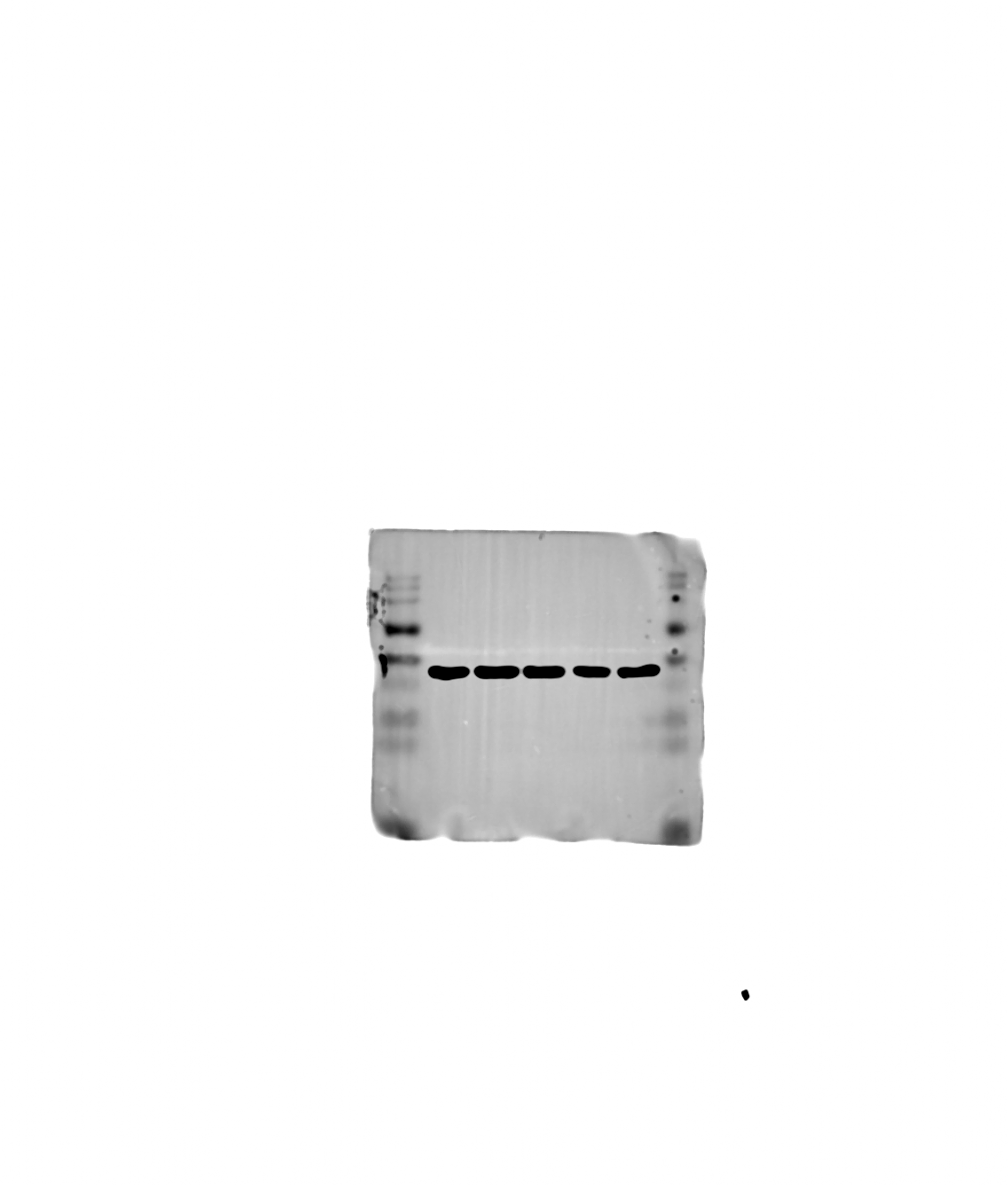

Supplement: Supplementary file 1 [file biomolecules-14-01102-s001.zip › Western Blot original images/STAT3/STAT3-1/β-actin.tif]

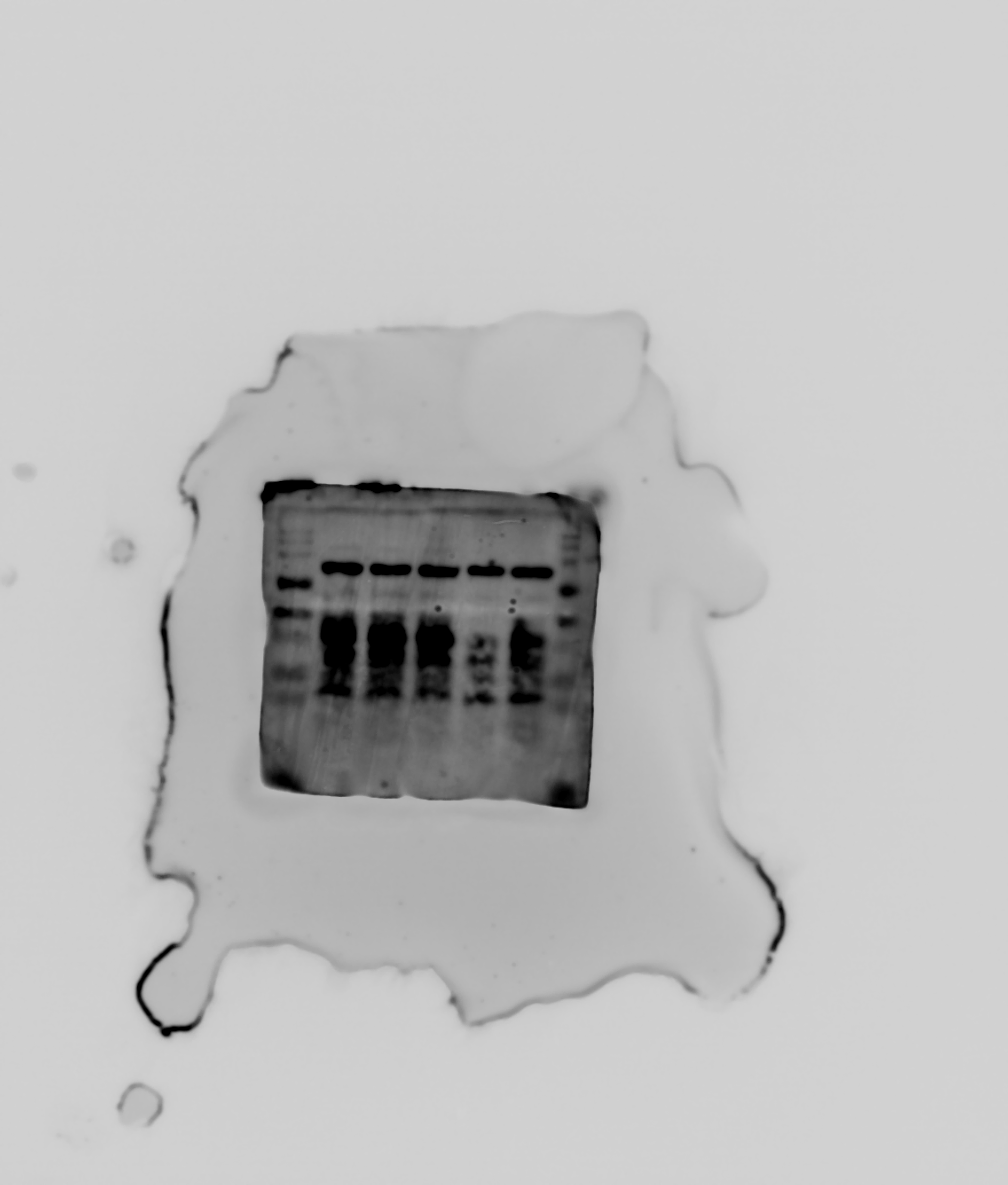

Supplement: Supplementary file 1 [file biomolecules-14-01102-s001.zip › Western Blot original images/STAT3/STAT3-2/STAT3.tif]

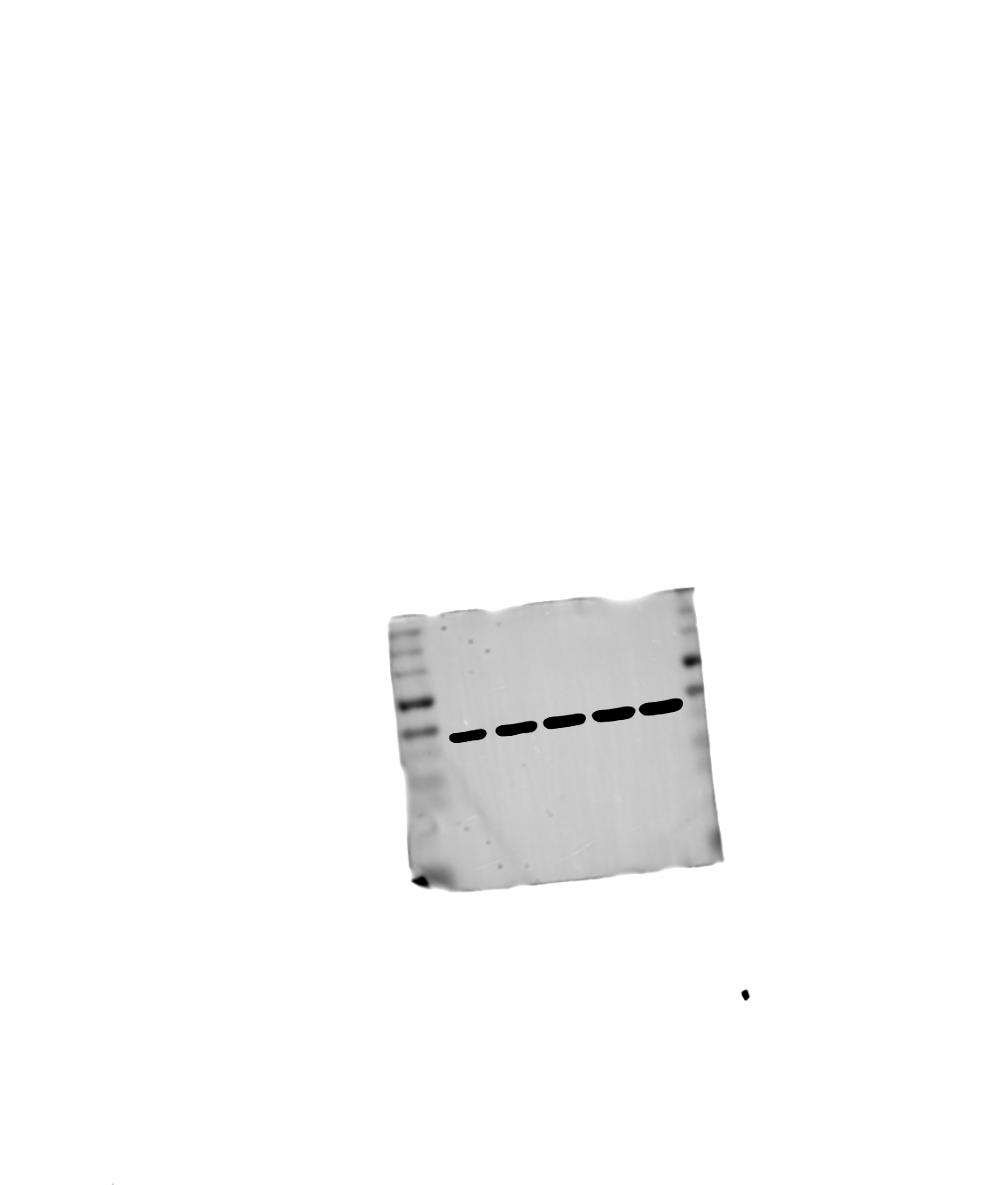

Supplement: Supplementary file 1 [file biomolecules-14-01102-s001.zip › Western Blot original images/STAT3/STAT3-2/β-actin.tif]

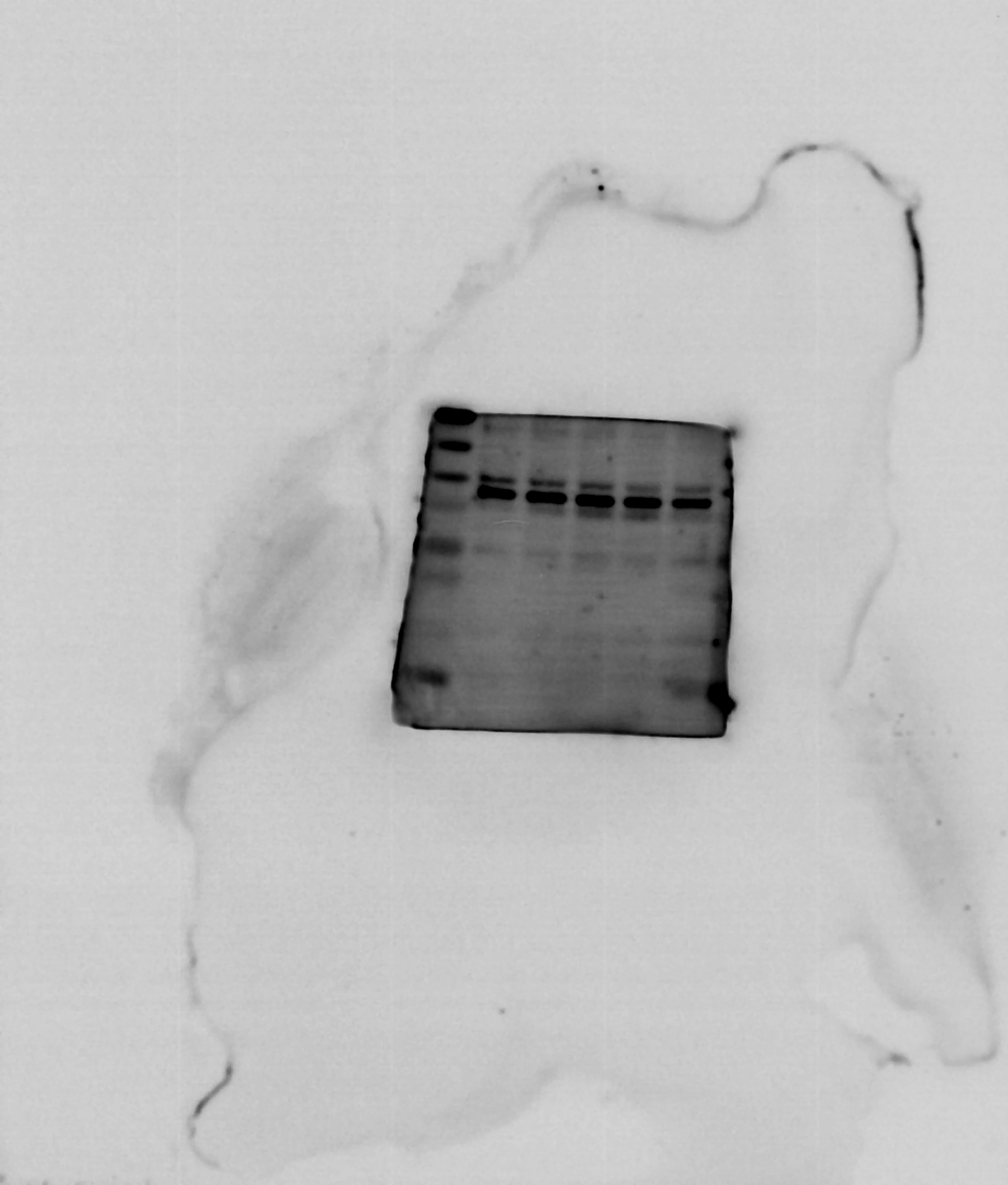

Supplement: Supplementary file 1 [file biomolecules-14-01102-s001.zip › Western Blot original images/STAT3/STAT3-3/STAT3.tif]

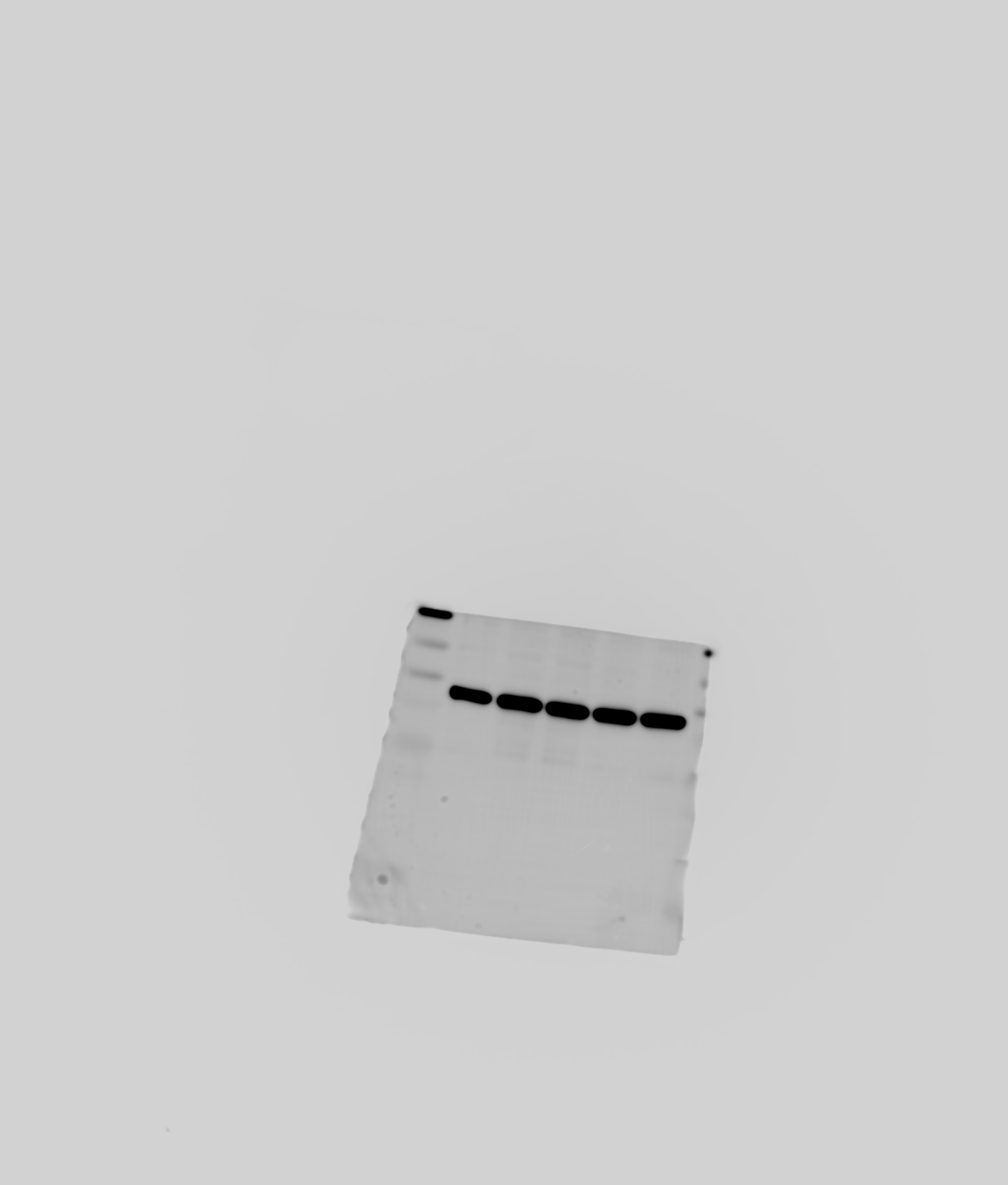

Supplement: Supplementary file 1 [file biomolecules-14-01102-s001.zip › Western Blot original images/STAT3/STAT3-3/β-actin.tif]
